# PbrNAC34a- PbrMYB3/65- PbrACO2 cascade plays a role in citrate difference between the pericarp and cortex tissues of pear (*P. bretschneideri* Rehd.) fruit

**DOI:** 10.1186/s43897-025-00177-9

**Published:** 2025-10-10

**Authors:** Xu Zhang, Luting Jia, Suling Zhang, Lijuan Zhu, Weilin Wei, Bing Yang, Weiqi Luo, Savithri U. Nambeesan, Xin Qiao, Li Jiang, Christopher Ference, Min Ma, Libin Wang, Shaoling Zhang

**Affiliations:** 1 Sanya Institute of Nanjing Agricultural University, National Key Laboratory of Crop Genetics & Germplasm Enhancement and Utilization, College of Horticulture, Nanjing Agricultural University, Nanjing, Jiangsu 210095 China; 2 College of Horticulture, Shanxi Agricultural University, Taigu, Jinzhong, Shanxi 030801 China; 3 Xinjiang Alar Gather Red Fruit Industry Co., Ltd., Alar, Xinjiang 843300 China; 4 Fruit Research Institute, Fujian Academy of Agricultural Sciences, Fuzhou, Fujian 350013 China; 5 Center for Integrated Pest Management, North Carolina State University, Raleigh, NC 27606 USA; 6 Department of Horticulture, University of Georgia, Athens, GA 30602 USA; 7 Department of Plant Pathology, University of Florida, Gainesville, FL 32611 USA; 8 School of Food and Biological Engineering, Hezhou University, Hezhou, Guangxi 542899 China

**Keywords:** Citrate isomerization, Citrate difference, Fruit development, PbrNAC34a-PbrMYB3/65-*PbrACO2* cascade, Pericarp and cortex tissues, *Pyrus bretschneideri* Rehd

## Abstract

Citrate is critical to the flavor of horticultural fruit and governed by ACO. However, the specific ACO and its upstream regulators involved in citrate metabolism during pear (*Pyrus* spp.) fruit development remained uncharacterized. This study identified and characterized six *PbrACOs* from the *Pyrus bretschneideri* Rehd. genome. Comprehensive analyses of citrate levels, cyt/mitACO activities, and *PbrACOs* expression profiles in the pericarp and cortex tissues of developing 'Yali' and 'Dangshansuli' fruits revealed *PbrACO2* as a candidate gene. Subsequently, *PbrACO2* was confirmed as a mitochondrial aconitase catalyzing citrate-to-isocitrate conversion in vitro and in vivo. Analysis of differentially expressed transcription factors (TFs) and *cis*-acting elements in the *PbrACO2* promoter identified nuclear PbrMYB3 and PbrMYB65, derived from whole genome duplication/segmental duplication, as candidate upstream regulators. These MYB TFs, without direct relationship, bound, as monomers, to the same two MYB-binding sites in the *PbrACO2* promoter to activate its transcription, thereby promoting citrate isomerization in pear and tomato. Further investigation revealed that *PbrMYB3* and *PbrMYB65* are transcriptionally regulated by PbrNAC34a. Given their tissue-dependent expression profiles, the PbrNAC34a-PbrMYB3/65-*PbrACO2* cascade partially accounts for citrate differences between pear fruit pericarp and cortex tissues. These findings enhance understanding of citrate accumulation in *Rosaceae* fruit and provide genetic resources for pear breeding.

**Supplementary Information:**

The online version contains supplementary material available at 10.1186/s43897-025-00177-9.

## Core

*PbrMYB3* and *PbrMYB65 *could bind to the same two MYB-binding sites in *PbrACO2 *promoter as monomer to activate its transcription, and thus promote citrate isomerization in pear and tomato. Further study revealed that the expression of these two MYB TFswas under the control of PbrNAC34a. Considering their tissue-dependent expression profiles, the PbrNAC34a-PbrMYB3/65-*PbrACO2* cascade was partly responsible for citrate difference between the pericarp and cortex tissues of pear fruit.

## Gene and accession numbers

Sequence data in this article could be retrieved from the pear genome database (http://peargenome.njau.edu.cn/) under the accession numbers: *PbrNAC34a *(Pbr026635.1), *PbrMYB3* (Pbr003370.1), *PbrMYB65* (Pbr000749.2), *PbrACO2 *(Pbr039718.1), and *PbrWRKY72 *(Pbr009294.1).

## Introduction

Pear (*Pyrus* spp.), an economically significant temperate fruit originating from southwestern China, is cultivated globally and valued for its distinctive organoleptic properties (Li et al*.*, 2022b; Niu et al., 2024; Qian et al. 2021). Together with soluble sugars, malate and citrate serve as the primary organic acids contributing to pear fruit flavor formation (Wu et al., 2022). During fruit development of *P. bretschneideri* Rehd. cv. 'Dangshansuli', malate levels exhibited a general declining trend, while citrate demonstrated an inverse pattern (Zhang et al., 2021). Similar patterns were observed during the maturation of *P. pyrifolia* cv. 'Yandangxueli' and 'Gengtouqing' fruits (Lu et al. 2011).

Citrate biosynthesis in horticultural fruit begins with the β-carboxylation of phosphoenolpyruvate (PEP) to oxaloacetate (OAA), catalyzed by cytosolic phosphoenolpyruvate carboxylase (PEPC) (Fig. S1) (Etienne et al. 2013; Tahjib-Ul-Arif et al. 2021). Subsequently, mitochondrial citrate synthase (CS), the first enzyme in the tricarboxylic acid (TCA) cycle, catalyzes the addition of an acetyl group from acetyl-CoA to OAA, generating citrate (Fig. S1) (Etienne et al. 2013; Tahjib-Ul-Arif et al. 2021). However, studies have not identified correlations between CS activity (or *PEPC* mRNA abundance) and citrate content during fruit maturation or among low-/high-citrate cultivars (Hussain et al. 2017; Roongruangsri et al. 2012). Consequently, citrate levels in developing fruit may be regulated by the downstream steps of citrate biosynthesis (Hussain et al. 2017).

Following synthesis, citrate undergoes sequential conversion by aconitase (ACO) and isocitrate dehydrogenase (IDH) into α-ketoglutarate, with isocitrate as an intermediate (Fig. S1) (Etienne et al. 2013; Tahjib-Ul-Arif et al. 2021). Plant ACOs, present in cytosol (cytACOs) and/or mitochondria (mitACOs) (Fig. S1) (Etienne et al. 2013; Tahjib-Ul-Arif et al. 2021; Wang et al. 2016), are classified into five subfamilies (Terol et al. 2010). For IDH, two types of isozymes have been identified in higher plants: the mitochondrial NAD^+^-dependent isozymes (NAD^+^-IDHs) and the NADP^+^-dependent isozymes (NADP^+^-IDHs). These isozymes are distributed across the cytosol, chloroplast, peroxisome, and mitochondrion (Fig. S1) (Gálvez and Gadal 1995; Gálvez et al. 1999; Hussain et al. 2017); with cytosolic NADP^+^-IDHs reportedly accounting for the majority of IDH activity in angiosperm and gymnosperm species (Pascual et al., 2018).

During the early developmental stages of *Citrus limon* cv. 'Eureka' fruit and *C. reticulata* cv. 'Sai Num Phueng' and 'See Thong' fruits, citrate accumulation corresponded with reduced mitACO and NAD^+^-IDH activities (Sadka et al. 2000a, b); conversely, increased activities of cytACO and NADP^+^-IDH appeared responsible for decreased citrate levels in later growth stages of these varieties (Roongruangsri et al. 2012; Sadka et al. 2000a). Additional research demonstrated that *CitACO3* expression levels were inversely correlated with citrate abundance in late developmental stages of *C. reticulata* Blanco cv. 'Ponkan' fruit (Li et al. 2017). This pattern was also observed during the maturation of *C. sinensis* cv. 'Newhall' and 'Skaggs Bonanza' fruits: citrate content progressively decreased, showing negative correlations with *CitACO3* and *CitIDH1* mRNA levels (Chen et al. 2013). Furthermore, compared to *C. sinensis* cv. 'Anliu' fruit, lower citrate levels in its bud mutant (cv. 'Hong Anliu') fruit development may be attributed to enhanced cytACO and NAD^+^/NADP^+^-IDH activities and upregulated expression of *ACO1-3*, *NAD*^+^*-IDH1-3,* and *NADP*^+^*-IDH1/3* (Guo et al. 2016). These findings suggest ACO and IDH are involved in citrate metabolism during fruit development. However, minimal citrate level changes were observed in plant tissues following antisense inhibition of a mitochondrial NAD^+^-IDH 1 gene (*SlIDH1*) or a cytosolic NADP^+^-IDH 1 gene (*SlICDH1*) from *Solanum lycopersicum* (Sienkiewicz-Porzucek et al. 2010; Sulpice et al. 2010) or overexpression of a cytosolic NADP^+^-IDH gene from *Pinus pinaster* (Pascual et al., 2018). Consequently, researchers have paid more attention to ACO's role in citrate metabolism during fruit maturation.

ACO, an iron-sulfur enzyme containing a 4 Fe-4S cluster, catalyzes the reversible isomerization of citrate to isocitrate with *cis*-aconitate as an intermediate (Wang et al. 2016). Plant ACOs typically exhibit a negative regulatory effect on citrate accumulation (Hussain et al. 2017). Overexpression of *CitACO3* reduced citrate accumulation in 'Ponkan' fruit (Li et al. 2017). Similar results were observed in *OsACO1*-overexpressing *Oryza sativa* (Senoura et al. 2020). Conversely, antisense suppression of *SlACO1* from *S. lycopersicum* or knockdown of *OsACO1* in rice decreased carbon flux through the TCA cycle, thereby enhancing citrate accumulation in these plants (Carrari et al. 2003; Senoura et al. 2020). Beyond regulating carbon status and flux, ACOs contribute to other physiological and developmental processes, including oxidative stress response and cell death (Wang et al. 2016).

The formation of horticultural fruit quality is regulated by transcription factors (TFs) through their binding to corresponding *cis*-acting elements in structural gene promoters (Jia et al. 2023). For example, PuWRKY31 from *P. ussuriensis* cv. 'Nanguo' binds to the W-box element (core motif, TTGACC/T) in the promoter of Sugars Will Eventually be Exported Transporter 15 gene (*PuSWEET15*) to induce its expression, resulting in soluble sugar accumulation in fruit (Li et al. 2020)*.* Similarly, PyHY5, a bZIP TF, enhances anthocyanin accumulation in *P. pyrifolia* cv. 'Yunhongli No. 1' fruit by directly interacting with the G-box element (core motif, CACGTG) in the promoters of anthocyanin-biosynthesis-related genes (*PyMYB10* and *PyWD40*), thereby initiating their transcription (Wang et al., 2020). Recent research has identified several TFs involved in citrate metabolism regulation in horticultural plants. AcNAC1 interacts with the core binding element in aluminum-activated malate transporter 1 gene (*AcALMT1*) promoter to activate its expression, leading to citrate accumulation during fruit maturation in *Actinidia chinensis* cv. 'Hongyang' and *A. deliciosa* cv 'Hayward' (Fu et al. 2023). Conversely, CitNAC62 collaborates with CitWRKY1 in citrate degradation through upregulation of *CitACO3* transcription (Li et al. 2017). However, the molecular mechanism of citrate metabolism during pear fruit development remains incompletely understood.

This study examined the function of *PbrACO2* and its upstream regulators, including PbrMYB3, PbrMYB65, and PbrNAC34a, in citrate metabolism within the pericarp and cortex tissues during fruit maturation of *P. bretschneideri* Rehd. The findings revealed that the tissue-specific expression pattern of the PbrNAC34a-PbrMYB3/65-*PbrACO2* cascade likely accounts for the differential citrate concentrations between pear fruit pericarp and cortex tissues.

## Results

### Evolution and characteristics of plant ACOs

A total of 66 ACOs, which emerged in the plant kingdom approximately 1,160 million years ago (MYA) (Fig. S2A), were identified from 18 plant species, and could be categorized into five subgroups (A, B, C, D & E) (Fig. S2B; Table S2). Five horticultural plant species were subsequently selected for detailed analyses.

As shown in Fig. S2B and Table S3, *P. bretschneideri*, *Prunus persica*, *Musa acuminata*, *Vitis vinifera*, and *A. chinensis* contained six, three, six, five, and five *ACOs*, respectively. These genes originated from whole genome duplication (WGD)/segmental and dispersed duplications, and were distributed across five, three, five, four, and four chromosomes in pear, peach, banana, grape, and kiwifruit genomes, respectively, exhibiting diverse physio-biochemical characteristics (Fig. S3A-B; Table S3-S4). 88 *cis*-acting elements were identified from their promoters, which were classified into eight functional categories (Fig. S3C). Comparative analyses of upstream regions of the paralogous gene pairs revealed promoter divergence (Fig. S4). This variation might lead to distinct regulatory mechanisms, thereby enhancing plant adaptation to various developmental processes and (a)biotic stresses (Qiao et al. 2015). Furthermore, at least nine exons were present in their genomic sequences, except for *PbrACO6*, which contained only one exon (Fig. S3D). Additionally, five (Motif 1, 3, 7, 8, and 11) out of thirteen motifs were conserved among the 'Aconitase' domains in 25 ACOs from five horticultural plant species (note: at least one motif was detected in each 'Aconitase' domain) (Fig. S3E; Table S5).

Subsequently, we analyzed the expression profiles of *PbrACOs* in several different tissues and their responses to light and temperature treatments. As shown in Fig. S5 and Table S6, except for *PbrACO6*, all other members exhibited expression across six tissues of 'Yali' pear, including 15-DAFB fruit, petal, stem, leaf, ovary, and stigma, with tissue-specific expression patterns. Furthermore, *PbrACO1-5* demonstrated varying transcriptional responses to light exposure and temperature treatment. As shown in Fig. S6A and Table S7, light exposure enhanced *PbrACO2* expression while suppressing *PbrACO3* expression (Fig. S6A; Table S7); the other three members showed no significant changes after light treatment (Fig. S6A; Table S7). Similarly, *PbrACO5* mRNA levels progressively decreased with temperature elevation (from 0 ℃ to 25 ℃ to 53 ℃), while low temperature (0 ℃) inhibited *PbrACO3* transcription (Fig. S6B; Table S8); meanwhile, *PbrACO1*, *PbrACO2*, and *PbrACO4* transcripts remained stable after high/low temperature treatment (Fig. S6B; Table S8).

### Characterization of *PbrACO2* as the candidate gene involved in citrate isomerization in the developing pear fruit

**Fig. 1:**
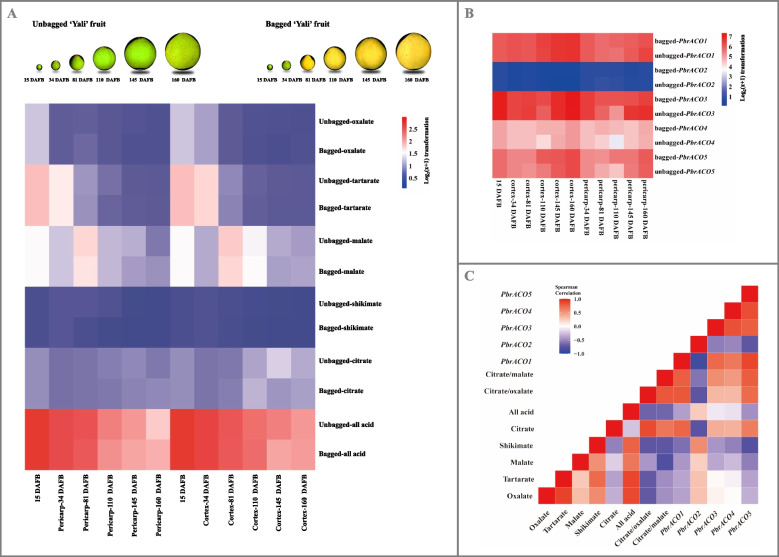
Dynamic change of citrate metabolism during the (un)bagged ‘Yali’ fruit development. **A** Organic acid content. Color scale represents normalized log2-transformed (mean value of three biological replicates + 1), where red, blue, and white colors indicate high, low, and medium expression levels, respectively. **B** *PbrACOs* expression profiles. Data, adapted from transcriptome assay, represent the mean value of three biological replicates; and color scale represents normalized log2-transformed (mean FPKM + 1), where red, blue, and white colors indicate high, low, and medium expression levels, respectively. **C** Correlations among attributes. Spearman correlations among different attributes are visualized as a heatmap, in which negative correlations are represented in blue color and positive in red color. ‘Yali’ pear were bagged with triple-layer paper bags at 34 DAFB, while the unbagged fruit at the same positions were labelled as well. Pericarp and cortex tissues were sampled at six developmental stages, including 15 DAFB, 34 DAFB, 81 DAFB, 110 DAFB, 145 DAFB, and 160 DAFB

To explore the molecular mechanism of citrate metabolism, we initially analyzed the accumulation patterns of organic acids in the pericarp and cortex tissues during the (un)bagged 'Yali' fruit development. As illustrated in Fig. 1A, Fig. S7-S8, and Table S9, several organic acids, including oxalate, tartarate, malate, and shikimate, showed decreasing trends in both tissues concurrent with increasing citrate percentage and citrate-to-malate/oxalate ratio; conversely, citrate demonstrated an increasing accumulation trend (Fig. 1A; Table S9). Bagging treatment had minimal impact on the compositions and accumulation patterns of organic acids throughout fruit maturation (Fig. 1A; Table S9). However, the cortex tissue of the (un)bagged developing fruit contained higher citrate levels than the pericarp tissue, accompanied by an increased citrate-to-malate ratio (Fig. 1A; Fig. S8; Table S9).

Subsequently, we sought to identify the candidate *PbrACO* responsible for the aforementioned phenomenon. As shown in Fig. 1B and Table S10, five *PbrACOs* were expressed during fruit maturation, exhibiting distinct expression patterns. Among these, *PbrACO2* expression levels were consistently higher in the pericarp tissue of the (un)bagged developing fruit compared to the cortex tissue (fold change ≥ 2.0 and FRD < 0.01; Fig. 1B; Table S10). Conversely, *PbrACO1* and *PbrACO5* showed opposite patterns (Fig. 1B; Table S10). Further analyses revealed strong positive correlations between citrate content and mRNA abundances of *PbrACO1* (correlation coefficients = 0.75) and *PbrACO5* (correlation coefficients = 0.64), but a negative correlation with *PbrACO2* expression level (correlation coefficient = -0.76) (Fig. 1C). Similar to the developing 'Yali' fruit, *PbrACO2* was the only member whose cortex tissue expression demonstrated positive correlations with cytACO and mitACO activities (coefficient = 0.46 and 0.08) but negative association with citrate content (coefficient = -0.41) during 'Dangshansuli' fruit development (Fig. S9 and Table S9-S10). Real-time quantitative polymerase chain reaction (RT-qPCR) assay results confirmed the accuracy of transcriptome findings regarding *PbrACOs* expression patterns during *P. bretschneideri* Rehd. fruit maturation (Fig. S10).

Collectively, these results suggested that *PbrACO2*, whose CDS and protein sequences showed high identity between the two cultivars (Fig. S11A and S12A), might be the candidate gene involved in citrate isomerization, resulting in differential citrate contents in the pericarp and cortex tissues of developing *P. bretschneideri* Rehd. fruit.

### Functional validation of *PbrACO2*

**Fig. 2:**
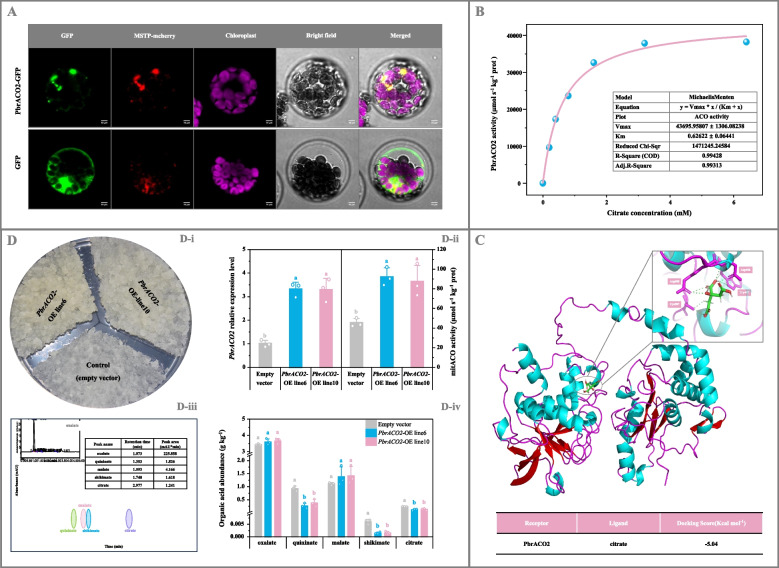
Functional validation of *PbrACO2*. **A** Subcellular localization of PbrACO2. MSTP-mcherry was used as the mitochondrial marker (Sun et al. 2022). Bar, 10 μm. **B** Michaelis–Menten curve for citrate conversion by PbrACO2 in vitro. **C** Binding model of PbrACO2 with citrate. The green dotted line indicates the hydrogen bond interaction. **D** Functional validation of *PbrACO2* in pear calli*.* (D-i) Growth status of pear calli. (D-ii) *PbrACO2* expression level and mitACO activity. (D-iii) Chromatogram of sample. (D-iv) Organic acid abundance. Calli transformed with the empty vector was used as the control; and *PbrACO2* expression level in the control calli is set as 1.0 for RT-qPCR assay. Data represent mean value ± standard deviation (SD) of three biological replicates, and vertical bars labelled with the same letter are not significantly different between samples (*p* < 0.05)

As shown in Fig. 2A, PbrACO2-GFP exhibited the identical subcellular localization to the mitochondrial marker MSTP-mcherry (Sun et al. 2022) in *Arabidopsis* protoplasts, indicating its mitochondrial localization.

To evaluate its catalytic properties, the recombinant His-PbrACO2 protein was obtained through prokaryotic expression. In vitro experiments demonstrated that PbrACO2 catalyzed the conversion of citrate, with *K*_*m*_ and *V*_*max*_ of 0.63 mM and 43.70 mmol s^-1^ kg^-1^ protein, respectively (Fig. 2B). The Cys^307^, Arg^405^, Asp^406^, and Trp^407^ residues in PbrACO2 potentially interact with citrate through hydrogen bonds, with a docking score of -5.04 kcal mol^-1^, contributing to the catalytic reaction (Fig. 2C; Fig. S13; Table S11).

Subsequently, we examined the role of *PbrACO2* in cellular citrate metabolism. As illustrated in Fig. S14A, transient overexpression of *PbrACO2* in pear fruit significantly increased mitACO activity, inhibiting citrate accumulation; conversely, the opposite effect was observed in *PbrACO2*-silenced fruit (Fig. S14B). To validate these findings, *PbrACO2-*overexpressing pear calli and tomato fruit with stable inheritance were generated. Overexpression of *PbrACO2* in pear calli enhanced mitACO activity, resulting in lower citrate levels compared to control calli; furthermore, quininate and shikimate formation was inhibited in the overexpressing calli, while malate and oxalate content remained unchanged among samples (Fig. 2D). Comparable results were observed in *PbrACO2-*transgenic tomato fruit, which exhibited increased mitACO activity but decreased citrate content (Fig. S15A), without affecting malate metabolism (data not shown). Additionally, color transition was suppressed in the transgenic tomato fruit (Fig. S15A-i).

### Identification of PbrMYB3 and PbrMYB65 as the candidate upstream regulators of *PbrACO2*

The expression of structural genes is transcriptionally regulated by upstream TFs through interaction with related *cis*-acting elements in their promoters (Jia et al. 2023). Based on transcriptome analysis, 50 TFs, whose mRNA abundances in the pericarp tissue were consistently higher (46) or lower (4) than those in the cortex tissue of 'Yali' fruit, were identified from the pear genome (fold change ≥ 2.0 and FRD < 0.01; Fig. S16A-B; Table S12). Among these, the expression levels of *Pbr003370.1* and *Pbr000749.2* showed strong positive correlations with *PbrACO2* transcript abundance during *P. bretschneideri* Rehd. fruit development (correlation coefficient > 0.8; Fig. S16B). Following Cao et al. (2016), *Pbr003370.1* and *Pbr000749.2* were designated as *PbrMYB3* and *PbrMYB65*, respectively. Using the PlantCARE database, two MYB-binding sites (MYBCORE box motifs, CAACCG) were identified in the *PbrACO2* promoter, both predicted to interact with PbrMYB3 and PbrMYB65 according to PlantRegMap database analysis (Fig. S16C). These findings suggest that these two MYB TFs may act as potential upstream regulators of *PbrACO2*. Further analysis revealed high sequence identity in their CDS and protein sequences between 'Yali' and 'Dangshansuli' fruits (Fig. S11B-C and S12B-C).

### PbrMYB65 bound to the *PbrACO2* promoter and then activated its expression

**Fig. 3:**
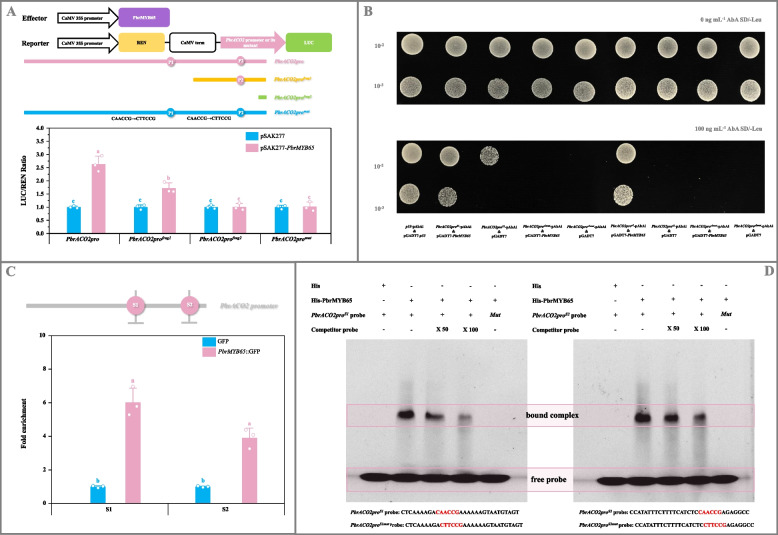
Confirmation of PbrMYB65 as the upstream regulator of *PbrACO2*. **A** Dual-luciferase assay. *PbrMYB65* CDS was introduced into the pSAK277 vector, while *PbrACO2* promoter fragments of different lengths, containing different numbers of the possible PbrMYB65**-**binding sites (*PbrACO2pro*, *PbrACO2pro*^*frag1*^, and *PbrACO2pro*^*frag2*^) or the mutated binding sites (*PbrACO2pro*^*mut*^), into the pGreen 0800-LUC vector. Transformants containing the empty pSAK277 and each reporter were used as the controls. Data represents mean value ± SD of three biological replicates, and vertical bars labelled with the same letter are not significantly different between samples (*p* < 0.05). **B** Y1H assay. *PbrMYB65* CDS was amplified into the pGADT7 vector, while about 200-bp fragments of *PbrACO2* promoter, containing the wide-type (*PbrACO2pro*^*S1*^ and *PbrACO2pro*^*S2*^) or the mutated PbrMYB65-binding sites (*PbrACO2pro*^*S1 mut*^ and *PbrACO2pro*^*S2 mut*^), were inserted into the bait vector pAbAi. Yeast cell co-transformed with pGADT7-*p53* & *p53*-AbAi was used as a positive control, while yeast cells co-transformed with the empty pGADT7 vector and each bait as the negative controls. **C** ChIP-qPCR analyses. Calli overexpressing the empty vector was used as a negative control. Data represents mean value ± SD of three biological replicates, and vertical bars labelled with the same letter are not significantly different between samples (*p* < 0.05). **D** EMSA assay. FAM luciferase-labelled *PbrACO2* promoter fragments, containing the wide-type and the mutated PbrMYB65-binding sites, were named as *PbrACO2pro*^*S1/S2*^ probes and *PbrACO2pro*^*S1 mut/S2 mut*^ probes, respectively, while the unlabeled *PbrACO2* promoter fragments were used as competitor probes. The presence and absence of His protein, His-PbrMYB65 protein, labeled probe, or competitor probe are indicated by “ + ” and “ − ”, respectively. Competitor probe concentrations are 50-fold (50 ×) and 100-fold (100 ×) those of the labeled probe

Based on its highest correlation coefficient with *PbrACO2* as *P. bretschneideri* Rehd. fruit matured (correlation coefficient = 0.84; Fig. S16B), PbrMYB65 was selected for further investigation. As illustrated in Fig. 3A, a 160% increase in Dual-luciferase/Renilla (LUC/REN) ratio was detected in tobacco leaf co-transformed with *PbrMYB65* and reporter containing *PbrACO2* promoter compared to the control, with this increase correlating positively to the number of potential PbrMYB65-binding sites; however, the enhanced LUC/REN ratio was eliminated following mutation of the two potential binding sites (CAACCG → CTTCCG). In the yeast one-hybrid (Y1H) assay, the positive control (pGADT7-*p53* & *p53*-pAbAi), negative controls (pGADT7 & *PbrACO2pro*^*S1*^-pAbAi, pGADT7 & *PbrACO2pro*^*S2*^-pAbAi), bait-prey co-transformants (pGADT7-*PbrMYB65* & *PbrACO2pro*^*S1*^-pAbAi, pGADT7-*PbrMYB65* & *PbrACO2pro*^*S2*^-pAbAi), and co-transformants containing the mutated elements (pGADT7 & *PbrACO2pro*^*S1 mut*^-pAbAi, pGADT7 & *PbrACO2pro*^*S2 mut*^-pAbAi, pGADT7-*PbrMYB65* & *PbrACO2pro*^*S1 mut*^-pAbAi, pGADT7-*PbrMYB65* & *PbrACO2pro*^*S2 mut*^-pAbAi) exhibited normal growth on SD/-Leu medium (Fig. 3B). Upon addition of Aureobasidin A (AbA), growth was inhibited in negative controls and transformants containing mutated binding elements, while the positive control and bait-prey co-transformants remained unaffected (Fig. 3B). Chromatin immunoprecipitation-quantitative PCR (ChIP-qPCR) analysis revealed more than three-fold enrichment in *PbrACO2* promoter fragments containing the PbrMYB65-binding sites compared to controls (Fig. 3C). In vitro electrophoretic mobility shift assay (EMSA) demonstrated formation of protein-DNA complexes when His-PbrMYB65 was incubated with labeled probes, with binding gradually diminishing as unlabeled competitor probe concentrations increased (Fig. 3D); nevertheless, these complexes disappeared following mutation of the PbrMYB65-binding sites (Fig. 3D).

Collectively, these findings indicate that PbrMYB65 interacts with the two predicted PbrMYB65-binding sites in the *PbrACO2* promoter to activate its transcription.

### Functional validation of *PbrMYB65 *in vivo

**Fig. 4:**
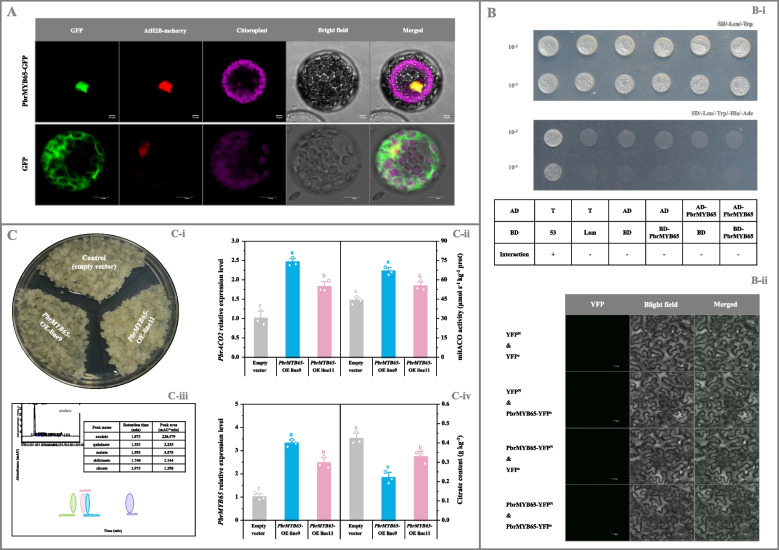
Functional validation of *PbrMYB65*. **A** Subcellular localization of PbrMYB65. AtH2B-mcherry was used as the nuclear marker (Liu et al. 2007). Bar, 10 μm. **B** PbrMYB65 self-interaction determination. (B-i) Y2H assay. Transformants containing AD-*T* & BD-*53*, AD-*T* & BD-*Lam*, AD & BD, AD & BD-*PbrMYB65*, and AD-*PbrMYB65* & BD were used as the controls. (B-ii) BiFC analyses. Transformants containing YFP^N^ & YFP^C^, YFP^N^ & *PbrMYB65*-YFP^C^, and *PbrMYB65*-YFP^N^ & YFP^C^ were used as the controls. Bar, 20 μm. **C** Functional validation of *PbrMYB65* in pear calli*.* (C-i) Growth status of pear calli. (C-ii) *PbrACO2* expression level and mitACO activity. (C-iii) Chromatogram of sample. (C-iv) *PbrMYB65* expression level and citrate abundance. Calli transformed with the empty vector was used as the control; and the expression levels of *PbrACO2* and *PbrMYB65* in the control calli are set as 1.0 for RT-qPCR assay. Data represents mean value ± SD of three biological replicates, and vertical bars labelled with the same letter are not significantly different between samples (*p* < 0.05)

As illustrated in Fig. 4A, PbrMYB65-GFP exhibited the identical subcellular localization to the nuclear marker AtH2B-mcherry (Liu et al. 2007) in *Arabidopsis* protoplasts, indicating nuclear localization. Additionally, the lack of PbrMYB65 self-interaction in yeast two-hybrid (Y2H) assay suggests that it exists in plant cell as a monomer (Fig. 4B).

To elucidate the physiological function of *PbrMYB65 *in vivo, transformations were performed in pear fruit, calli, and tomato. Compared to control fruit, *PbrMYB65*-overexpressing pear fruit exhibited elevated *PbrACO2* mRNA levels but reduced citrate content (Fig. S17A); conversely, silencing of *PbrMYB65* in pear fruit produced opposite results (Fig. S17B). Similar results were observed following *PbrMYB65* overexpression in pear calli and tomato fruit (Fig. 4C; Fig. S15B). Malate content remained unchanged across samples (data not shown). Furthermore, *PbrMYB65*-transgenic tomato fruit displayed reduced color development (Fig. S15B-i).

### PbrMYB3 demonstrated a similar function in citrate metabolism as PbrMYB65

**Fig. 5:**
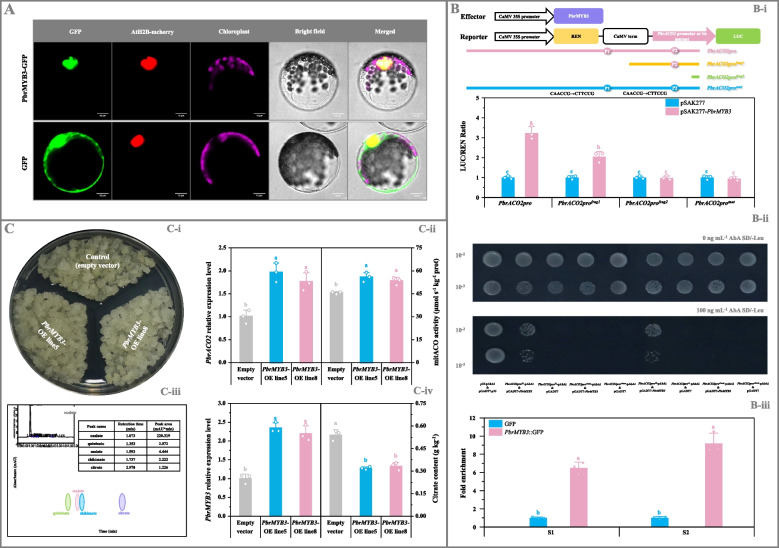
Determination of PbrMYB3’s role in citrate metabolism in pear. **A** Subcellular localization of PbrMYB3. AtH2B-mcherry was used as the nuclear marker (Liu et al. 2007). Bar, 10 μm. **B** Confirmation of PbrMYB3 as the upstream regulator of *PbrACO2*. (B-i) Dual-luciferase assay. *PbrMYB3* CDS was introduced into the pSAK277 vector, while *PbrACO2* promoter fragments of different lengths, containing different numbers of the possible PbrMYB3-binding sites (*PbrACO2pro*, *PbrACO2pro*^*frag1*^, and *PbrACO2pro*^*frag2*^) or the mutated binding sites (*PbrACO2pro*^*mut*^), into the pGreen 0800-LUC vector. Transformants containing the empty pSAK277 and each reporter were used as the controls. (B-ii) Y1H assay. *PbrMYB3* CDS was amplified into the pGADT7 vector, while about 200-bp fragments of *PbrACO2* promoter, containing the wide-type (*PbrACO2pro*^*S1*^ and *PbrACO2pro*^*S2*^) or the mutated PbrMYB3-binding sites (*PbrACO2pro*^*S1 mut*^ and *PbrACO2pro*^*S2 mut*^), were inserted into the bait vector pAbAi. Yeast cell co-transformed with pGADT7-*p53* & *p53*-AbAi was used as a positive control, while yeast cells co-transformed with the empty pGADT7 vector and each bait as the negative controls. (B-iii) ChIP-qPCR analyses. Calli overexpressing the empty vector was used as a negative control. **C** Functional validation of *PbrMYB3* in pear calli. (C-i) Growth status of pear calli. (C-ii) *PbrACO2* expression level and mitACO activity. (C-iii) Chromatogram of sample. (C-iv) *PbrMYB3* expression level and citrate abundance. Calli transformed with the empty vector was used as the control; and the expression levels of *PbrACO2* and *PbrMYB3* in the control calli are set as 1.0 for RT-qPCR assay. Data represents mean value ± SD of three biological replicates, and vertical bars labelled with the same letter are not significantly different between samples (*p* < 0.05).

Subsequently, we investigated the role of PbrMYB3 in citrate metabolism, which was shown to interact with the same *cis*-acting elements as PbrMYB65 (Fig. S16C). As shown in Fig. 5A, PbrMYB3-GFP exhibited the identical subcellular localization to the nuclear marker AtH2B-mcherry (Liu et al. 2007) in *Arabidopsis* protoplasts, indicating its nuclear localization. Additionally, lack of PbrMYB3 self-interaction in the Y2H assay implies that it exists in plant cell as a monomer (Fig. S18).

Further experimentation revealed that the LUC/REN ratio in tobacco leaf co-transformed with *PbrMYB3* and reporter containing *PbrACO2* promoter increased approximately 220% compared to the control, with this increase correlating positively to the number of potential PbrMYB3-binding sites (Fig. 5B-i); however, this increase disappeared following mutation of the two binding sites (Fig. 5B-i). In the Y1H assay, the positive control (pGADT7-*p53* & *p53*-pAbAi), negative controls (pGADT7 & *PbrACO2pro*^*S1*^-pAbAi, pGADT7 & *PbrACO2pro*^*S2*^-pAbAi), bait-prey co-transformants (pGADT7-*PbrMYB3* & *PbrACO2pro*^*S1*^-pAbAi, pGADT7-*PbrMYB3* & *PbrACO2pro*^*S2*^-pAbAi), and co-transformants containing the mutated elements (pGADT7 & *PbrACO2pro*^*S1 mut*^-pAbAi, pGADT7 & *PbrACO2pro*^*S2 mut*^-pAbAi, pGADT7-*PbrMYB3* & *PbrACO2pro*^*S1 mut*^-pAbAi, pGADT7-*PbrMYB3* & *PbrACO2pro*^*S2 mut*^-pAbAi) exhibited normal growth on SD/-Leu medium (Fig. 5B-ii); upon AbA addition, growth was suppressed in negative controls and transformants with mutated binding elements, while the positive control and bait-prey co-transformants remained unaffected (Fig. 5B-ii). ChIP-qPCR analyses confirmed substantial enrichment (> six-fold) of PbrMYB3 in *PbrACO2* promoter fragments containing the PbrMYB3-binding sites (Fig. 5B-iii).

Consistent with observations in *PbrMYB65*-transgenic fruit and calli, *PbrMYB3* mRNA levels in transgenic pear tissues showed a positive correlation with *PbrACO2* expression and/or mitACO activity, while negatively correlating with citrate content (Fig. 5C; Fig. S19). Malate abundance remained unchanged across samples (data not shown). These findings suggest that PbrMYB3 functions as a monomer in binding the two predicted PbrMYB3-binding sites in the *PbrACO2* promoter, transcriptionally activating its expression and consequently reducing citrate accumulation in pear.

Given that *PbrMYB3* and *PbrMYB65* constitute a syntenic gene pair (Cao et al., 2016; Li et al. 2016), their relationship was examined. Despite high protein sequence identity (Fig. S20A), their promoter sequences differed (2000-bp sequences upstream of the transcriptional start sites (ATG) of *PbrMYB3* and *PbrMYB65*; Fig. S20B). Consistent with the absence of PbrMYB3-/PbrMYB65-binding sites (MYBCORE box motif, CAACCG) in their promoters, no significant increase in LUC/REN ratio was observed in tobacco leaf co-transformed with *PbrMYB3* (or *PbrMYB65*) and the reporter containing *PbrMYB65* promoter (or *PbrMYB3* promoter) (Fig. S21A). Furthermore, Y2H and bimolecular fluorescence complementation (BiFC) assays showed no interaction between PbrMYB3 and PbrMYB65 (Fig. S21B).

### PbrNAC34a was an upstream regulator of *PbrMYB3* and *PbrMYB65*

To identify the upstream regulators of these two MYB TFs, correlation analyses were conducted between *PbrMYB3*/*65* and 48 other differentially expressed TFs. As shown in Fig. S22, the expression levels of 18 members exhibited strong correlations with *PbrMYB3*, *PbrMYB65*, and *PbrACO2* mRNA levels during the maturation process of *P. bretschneideri* Rehd. fruit, including *Pbr000398.1*, *Pbr015697.1*, *Pbr020595.1*, *Pbr022437.1*, *Pbr027839.1*, *Pbr030208.1*, *Pbr038280.1*, *Pbr013255.1*, *Pbr000523.1*, *Pbr009294.1*, *Pbr011441.1*, *Pbr019293.1*, *Pbr038434.1*, *Pbr006028.1*, *Pbr013948.1*, *Pbr016310.4*, *Pbr010912.1*, and *Pbr026635.1* (absolute correlation coefficient > 0.6). Among these, *Pbr026635.1*, designated as *PbrNAC34a* by Gong et al. (2019), displayed relatively high coefficients with *PbrMYB3*, *PbrMYB65*, and *PbrACO2* (Fig. S22)*.* Furthermore, utilizing the PlantRegMap database and previous reports on the common sequences of the NAC-binding sites (Bi et al. 2023; Li et al. 2023), several potential PbrNAC34a-binding sites were identified from *PbrMYB3* and *PbrMYB65* promoters (2000-bp sequences upstream of the transcriptional start sites (ATG) (Fig. S23). These findings suggested that PbrNAC34a might function as their upstream regulator.

**Fig. 6:**
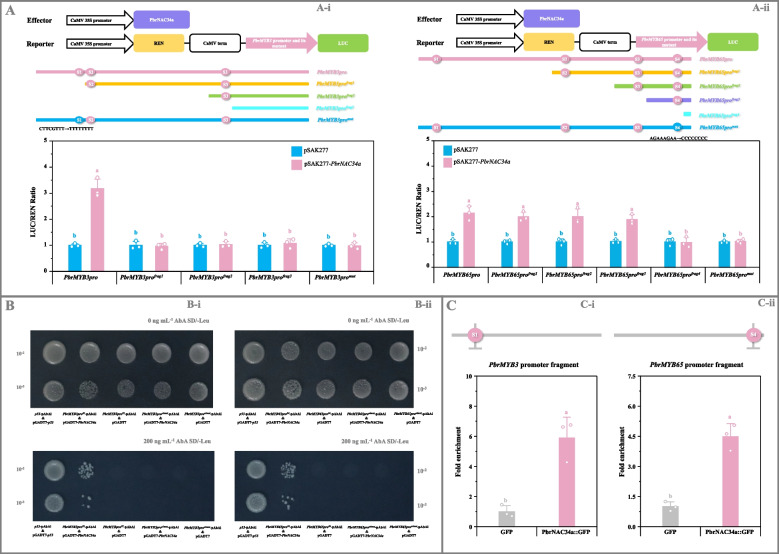
Identification and confirmation of PbrNAC34a as the upstream regulator of *PbrMYB3* and *PbrMYB65*. **A** Dual-luciferase assay for the activation of *PbrMYB3* (A-i) and *PbrMYB65* (A-ii) transcription by PbrNAC34a. *PbrNAC34a* CDS was introduced into the pSAK277 vector, while *PbrMYB3* and *PbrMYB65* promoter fragments of different lengths, containing different numbers of the possible PbrNAC34a-binding sites (*PbrMYB3* promoter fragments included *PbrMYB3pro*, *PbrMYB3pro*^*frag1*^, *PbrMYB3pro*^*frag2*^, and *PbrMYB3pro*^*frag3*^; *PbrMYB65* promoter fragments included *PbrMYB65pro*, *PbrMYB65pro*^*frag1*^, *PbrMYB65pro*^*frag2*^, *PbrMYB65pro*^*frag3*^, and *PbrMYB65pro*^*frag4*^) or the mutated binding sites (*PbrMYB3pro*^*mut*^ and *PbrMYB65pro*^*mut*^), into the pGreen 0800-LUC vector. Transformants containing the empty pSAK277 vector and each reporter were used as the controls. **B** Y1H assay of the interactions between PbrNAC34a and *PbrMYB3* promoter fragment (B-i) and between PbrNAC34a and *PbrMYB65* promoter fragment (B-ii). *PbrNAC34a* CDS was amplified into the prey vector pGADT7, while about 200-bp fragments of *PbrMYB3* and *PbrMYB65 promoters*, containing the wide-type (*PbrMYB3pro*^*S1*^ and *PbrMYB65pro*^*S4*^) or the mutated PbrNAC34a-binding sites (*PbrMYB3pro*^*S1 mut*^ and *PbrMYB65pro*^*S4 mut*^), were inserted into the bait vector pAbAi. Yeast cell co-transformed with pGADT7-*p53* and *p53*-AbAi was used as a positive control, while yeast cells co-transformed with the empty pGADT7 vector and each bait as the negative controls. **C** ChIP-qPCR analyses of the interactions between PbrNAC34a and *PbrMYB3* promoter fragment (C-i) and between PbrNAC34a and *PbrMYB65* promoter fragment (C-ii). Calli overexpressing the empty vector was used as a negative control. Data represent mean value ± SD of three biological replicates, and vertical bars labelled with the same letter are not significantly different between samples (*p* < 0.05)

To investigate the *cis*-acting elements responsible for the PbrNAC34a-induced activation of downstream gene expression, dual-luciferase reporter assays were conducted using *PbrMYB3* and *PbrMYB65* promoter fragments of varying lengths, containing different numbers of potential PbrNAC34a-binding sites. As illustrated in Fig. 6A, only fragments containing the 'CTTCGTTT' (site 1 (S1) in *PbrMYB3* promoter) and 'AGAAAGAA' (site 4 (S4) in *PbrMYB65* promoter) elements interacted with PbrNAC34a to initiate downstream gene transcription. Furthermore, the LUC/REN ratio increase ceased after the mutation of the binding site in *PbrMYB3* (CTTCGTTT → TTTTTTTT) or *PbrMYB65* (AGAAAGAA → CCCCCCCC) promoter (Fig. 6A). These findings confirmed the critical importance of these two elements for PbrNAC34a to activate *PbrMYB3* and *PbrMYB65* expression, prompting further investigation. In the Y1H assay, the positive control (pGADT7-*p53* & *p53*-pAbAi), negative controls (pGADT7 & *PbrMYB3pro*^*S1*^-pAbAi, pGADT7 & *PbrMYB65pro*^*S4*^-pAbAi), bait-prey co-transformants (pGADT7-*PbrNAC34a* & *PbrMYB3pro*^*S1*^-pAbAi, pGADT7-*PbrNAC34a* & *PbrMYB65pro*^*S4*^-pAbAi), and co-transformants containing the mutated elements (pGADT7 & *PbrMYB3pro*^*S1 mut*^-pAbAi, pGADT7 & *PbrMYB65pro*^*S4 mut*^-pAbAi, pGADT7-*PbrNAC34a* & *PbrMYB3pro*^*S1 mut*^-pAbAi, pGADT7-*PbrNAC34a* & *PbrMYB65pro*^*S4 mut*^-pAbAi) exhibited normal growth on SD/-Leu medium (Fig. 6B). Upon AbA addition, growth was suppressed in negative controls and transformants containing mutated binding elements, while the positive control and bait-prey co-transformants remained unaffected (Fig. 6B). ChIP-qPCR analyses revealed greater than four-fold enrichment in *PbrMYB3* and *PbrMYB65* promoter fragments containing the PbrNAC34a-binding sites compared to controls (Fig. 6C).

**Fig. 7:**
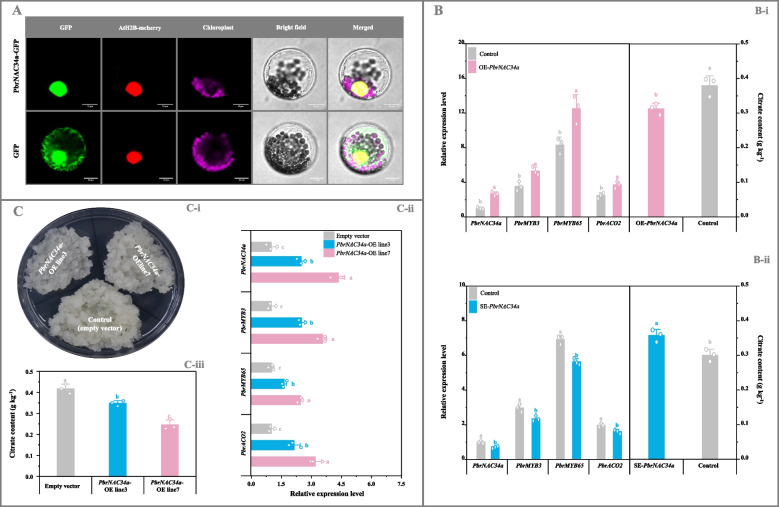
Function validation of PbrNAC34a. **A** Subcellular localization of PbrNAC34a. AtH2B-mcherry was used as the nuclear marker (Liu et al. 2007). Bar, 10 μm. **B** Functional validation of *PbrNAC34a* in pear fruit. (B-i) Transient overexpression of *PbrNAC34a*. ‘Yali’ fruit transformed with the empty pCAMBIA1300 vector containing a GFP tag was used as the control for the *PbrNAC34a*-overexpressing fruit. (B-ii) Transient silence of *PbrNAC34a*. Fruit co-transformed with the empty pTRV2 and pTRV1 vectors was used as the control for the *PbrNAC34a*-silenced fruit. **C** Functional validation of *PbrNAC34a* in pear calli. (C-i) Growth status of pear calli. (C-ii) Gene expression level. (C-iii) Citrate abundance. Calli transformed with the empty vector was used as the control. And the expression level of each gene in the control calli/fruit is set as 1.0 for RT-qPCR assay. Data represents mean value ± SD of three biological replicates, and vertical bars labelled with the same letter are not significantly different between samples (*p* < 0.05)

Subsequently, PbrNAC34a function was examined. As demonstrated in Fig. 7A, PbrNAC34a-GFP exhibited the identical subcellular localization to the nuclear marker AtH2B-mcherry (Liu et al. 2007) in *Arabidopsis* protoplasts, indicating nuclear localization. In vivo analysis revealed that *PbrNAC34a* overexpression in pear fruit enhanced expression levels of *PbrMYB3*, *PbrMYB65*, and *PbrACO2*, leading to reduced citrate levels compared to control fruit (Fig. 7B-i); conversely, *PbrNAC34a*-silenced fruit exhibited opposite results (Fig. 7B-ii). Comparable effects were observed in *PbrNAC34a*-overexpressing pear calli, where *PbrMYB3*, *PbrMYB65*, and *PbrACO2* transcription increased, resulting in decreased citrate content (Fig. 7C). These results confirmed PbrNAC34a's role as an upstream regulator of *PbrMYB3* and *PbrMYB65*.

## Discussion

Pear, belonging to the *Pomoideae* subfamily of *Rosaceae* family, is renowned for its distinctive flavor quality (Qian et al. 2021; Wang et al. 2021a, b). Taste quality encompasses the collective sensory attributes resulting from the stimulation of gustatory receptors on the tongue (Baldwin et al. 1998). As essential metabolites contributing to taste, malate and citrate were identified as the predominant organic acids in mature 'Yali' and 'Dangshansuli' fruits (Fig. 1A; Fig. S7 and S9A; Table S9). In agreement with Zhang et al. (2021), malate and citrate in the cortex of both cultivars exhibited inverse accumulation patterns during fruit development (Fig. 1A; Fig. S9A; Table S9).

With the publication of *P. bretschneideri* Rehd. genome and advancements in plant biotechnology, modification of plant phenotype through genetic engineering has become achievable (Li et al., 2022b). Among > 40,000 protein-coding genes identified from the pear genome (Wu et al., 2013), only a limited number have been functionally validated in organic acid metabolism. A cytosolic NAD^+^-dependent malate dehydrogenase gene (gene ID: *Pbr030554.1*) and a cytosolic NADP^+^-dependent malate enzyme gene (gene ID: *Pbr008772.1*) participated in malate biosynthesis and degradation, respectively (Wang et al. 2018). Additionally, an ALMT from white pear (gene ID: *Pbr020270.1*), situated in the plasma membrane, facilitated malate import into the sink cell (Xu et al. 2018). Nevertheless, understanding of citrate metabolism in developing pear fruit remains limited.

Previous studies suggest that citrate levels in horticultural fruit are regulated by ACOs (Hussain et al. 2017; Pascual et al., 2018; Sienkiewicz-Porzucek et al. 2010; Sulpice et al. 2010), which catalyze the reversible isomerization of citrate to isocitrate (Wang et al. 2016). Consistent with this observation, the mitochondrial PbrACO2, whose expression was positively correlated with cytACO and mitACO activities but negatively with citrate content during pear (*P. bretschneideri* Rehd.) fruit maturation, catalyzed the isomerization of citrate to isocitrate in vitro and in vivo (Fig. 1C; Fig. 2; Fig. S9D; Fig. S14 and S15A). Similar functionality was observed in homologues from other plants, including CitACO3 from citrus (Li et al. 2017), SlACO1 from tomato (Carrari et al. 2003), and OsACO1 from rice (Senoura et al. 2020), suggesting conservation of ACO function throughout plant evolution. However, other *PbrACO* isoforms, whose transcript levels were higher during *P. bretschneideri* Rehd. fruit maturation (Fig. 1B; Fig. S9C and Table S10), may also contribute significantly to citrate metabolism. Beyond transcriptional regulation, ACO activity undergoes posttranslational suppression by H_2_O_2_ and peroxynitrite (a potent oxidant and nitrating agent formed from superoxide anion and nitric oxide generated by mitochondria) (Bulteau et al. 2003; Han et al., 2005; Verniquet et al. 1991). H_2_O_2_ variations during pear development (Wang et al. 2021a, b) suggest that posttranslational modification of PbrACOs might also influence fruit citrate levels.

Plant TFs interact with corresponding *cis*-acting elements in the promoters of downstream genes to regulate their expression, thereby influencing fruit quality (Jia et al. 2023). Through transcription analyses and experimental validation, this study demonstrated that PbrMYB3 and PbrMYB65 bound, as monomers, to two identical MYB-binding sites in the *PbrACO2* promoter to activate its expression, subsequently suppressing citrate accumulation in pear and tomato (Figs. 3, 4, and 5; Fig. S15B and S16-S19). These findings establish these MYB TFs as upstream regulators of *PbrACO2*. This mechanism parallels the collaborative inhibition of citrate accumulation in 'Ponkan' fruit by CitWRKY1 and CitNAC62 through *CitACO3* regulation (Li et al. 2017). Furthermore, consistent with observations in the *AcNAC1*-mutagenetic kiwifruit showing reduced citrate levels (Fu et al. 2023), malate levels remained unchanged after transformation of pear/tomato fruit and/or calli with *PbrACO2* (or *PbrMYB3*, or *PbrMYB65*) gene (Fig. 2D; Fig. 4C-5C; Fig. S15), indicating malate metabolism may operate independently of citrate pathways. However, similar to the *AcNAC1*-mutagenetic kiwifruit, color transition was inhibited in *PbrACO2*/*PbrMYB65*-transgenic tomato fruit (Fig. S15), suggesting an association between citrate metabolism and this phenotype.

Previous research established that *PbrMYB3* and *PbrMYB65* constitute a syntenic gene pair originating from WGD/segmental duplication, with purifying selection serving as the primary evolutionary force (Cao et al., 2016; Li et al. 2016). Despite their highly identical protein sequences (Fig. S20A), no direct interaction was observed between PbrMYB3 and PbrMYB65 (Fig. S21). Gene duplication significantly contributes to gene family expansion, potentially leading to regulatory mechanism divergence that enhances plant adaptation to various developmental processes and (a)biotic stresses (Qiao et al. 2015). Similar to observations in *PbrACOs* (Fig. S3C and S4), the promoter sequences of *PbrMYB3* and *PbrMYB65* showed inconsistencies (Fig. S20B). Additional investigation revealed that PbrNAC34a binds to different *cis*-acting elements at distinct sites in the promoters of these MYB TFs, inducing their expression (Fig. 6; Fig. S22-S23). Analysis of their expression patterns across tissues and responses to abiotic stresses (Fig. S5 and S6; Table S6-S8) suggests distinct regulatory mechanisms for *PbrMYB3* and *PbrMYB65* expression in pear.

The study revealed no significant difference in organic acid accumulation between bagged and unbagged 'Yali' pear fruits (Fig. 1A; Table S9). However, the cortex tissue exhibited higher citrate levels compared to the pericarp tissue, corresponding with lower expression levels of *PbrACO2*, *PbrMYB3*, *PbrMYB65*, and *PbrNAC34a* (Fig. 1A-B; Fig. S16b; Table S9-S10 and S12). Previous research on 'Hongyang' kiwifruit demonstrated that elevated citrate content in the outer pericarp tissue relative to the core tissue correlated with upregulated transcription of *AcALMT1* and *AcNAC1* (Fu et al. 2023). Considering their roles in citrate metabolism, these results suggest that the PbrNAC34a-PbrMYB3/65-*PbrACO2* cascade mediates citrate differences between pear fruit pericarp and cortex tissues (Fig. 1–7; Fig. S14-S23; Table S9-10 and S12). Similar regulatory cascades have been documented in banana ripening ethylene biosynthesis (Wei et al., 2023) and grape berry sugar accumulation (Li et al., 2022a). Additionally, experimental validation confirmed that nuclear Pbr009294.1 (designated as *PbrWRKY72* by Huang et al. (2015)), despite showing strong positive correlations with *PbrMYB3* and *PbrMYB65* mRNA levels during white pear maturation (Fig. S22), did not regulate their transcription or citrate metabolism (Fig. S24).

**Fig. 8:**
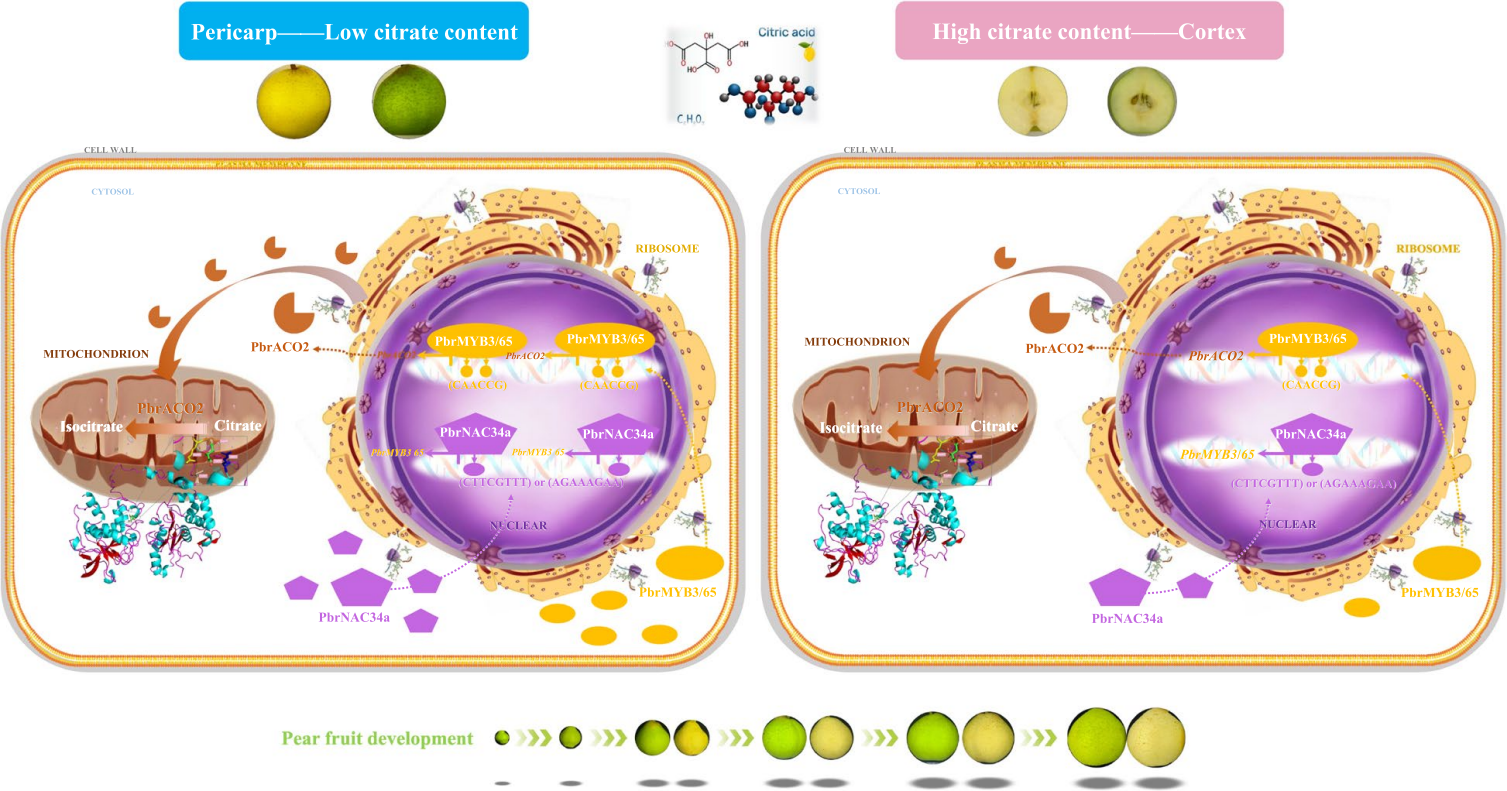
Schematic model on the PbrNAC34a-PbrMYB3/65-*PbrACO2* cascade regulating citrate difference between the pericarp and cortex tissues of the developing *P. bretschneideri* Rehd. fruit. During *P. bretschneideri* Rehd. fruit development, the nuclear PbrNAC34a binds to the promoters of *PbrMYB3* (binding element: CTTCGTTT) and *PbrMYB65* (binding element: AGAAAGAA), and then triggers their transcription. After translation in ribosome, these two MYB TFs are transported into the nucleus, where they interact with the same two MYB-binding sites (MYBCORE box motif, CAACCG) in *PbrACO2* promoter as monomer and subsequently activate its expression. After translation and then import into mitochondria, PbrACO2 isomerizes citrate into isocitrate, inhibiting citrate accumulation in pear. Due to the higher expression levels of *PbrACO2*, *PbrMYB3*, *PbrMYB65*, and *PbrNAC34a*, the pericarp tissue possessed lower citrate abundance than that in the cortex tissue

Based on the findings in this study, a schematic model has been proposed (Fig. 8). During *P. bretschneideri* Rehd. fruit development, nuclear PbrNAC34a binds to the promoters of *PbrMYB3* (binding element: CTTCGTTT) and *PbrMYB65* (binding element: AGAAAGAA), triggering their transcription. Following translation in the ribosome, these two MYB TFs are transported into the nucleus, where they interact with the same two MYB-binding sites (MYBCORE box motif, CAACCG) in the *PbrACO2* promoter as monomers and subsequently activate its expression. After translation and import into mitochondria, PbrACO2 isomerizes citrate into isocitrate, thereby inhibiting citrate accumulation in pear. Due to higher expression levels of *PbrACO2*, *PbrMYB3*, *PbrMYB65*, and *PbrNAC34a*, the pericarp tissue exhibits lower citrate abundance compared to the cortex tissue.

## Conclusion

This study demonstrates that PbrACO2, localized in the mitochondria, catalyzes citrate isomerization during *P. bretschneideri* Rehd. fruit development. Two MYB TFs, PbrMYB3 and PbrMYB65, bind, as monomers, to the same two MYB-binding sites in the *PbrACO2* promoter and initiate its expression, thereby reducing citrate levels in pear and tomato. Furthermore, PbrNAC34a functions as the upstream activator of the *PbrMYB3* and *PbrMYB65* genes. Based on their distinct expression profiles in two tissues, this study suggests that the PbrNAC34a-PbrMYB3/65-*PbrACO2* cascade regulates citrate differences between the pericarp and cortex tissues of pear.

## Materials and methods

### Bioinformatics analyses

ACOs from *Arabidopsis* were used as queries in the BLASTP against plant genome databases (https://phytozome-next.jgi.doe.gov/, https://plantgenie.org/, and http://peargenome.njau.edu.cn/) before confirmation of the conserved domain by the SMART database (http://smart.embl-heidelberg.de/) (Terol et al. 2010; Wang et al. 2016; Wang et al. 2021a, b). Physio-biochemical parameters were calculated using the ProtParam tool (https://web.expasy.org/protparam/) (Wang et al. 2018).

The timescale tree was constructed using TIMETREE (http://www.timetree.org/) (Kumar et al. 2017), and the phylogenetic tree was generated by MEGA7.0 software, employing the Neighbor-Joining (NJ) method with the poisson model (all other settings, including the number of bootstrap iterations (1000), remained default) (Zhang et al. 2019). Gene structure was visualized using the Gene Structure Display Server (http://gsds.cbi.pku.edu.cn/) (Hu et al., 2015); *cis*-acting elements were identified using the PlantCARE database (http://bioinformatics.psb.ugent.be/webtools/plantcare/html/) (Lescot et al. 2002); and motifs were characterized using the MEME Suite tool (https://meme-suite.org/meme/index.html) (Bailey et al. 2015). Divergence between upstream sequences of each paralogous gene pair was measured using the GATA program (Nix and Eisen 2005), with a window size set at seven and a lower cutoff score of 12 bits. The TF-binding sites in the gene promoter were predicted through the PlantRegMap database (http://plantregmap.gao-lab.org/) (Tian et al. 2020).

Chromosomal locations of plant *ACOs* were determined through genome annotation and visualized using Circos (Krzywinski et al. 2009). A methodology similar to that employed in the Plant Genome Duplication Database (PGDD) was utilized to analyze the syntenic relationship. The duplicated *ACOs* were classified into several categories: WGD/segmental, tandem, singleton, proximal, and dispersed (Zhang et al. 2019). MCScanX downstream analyses tools were employed to annotate the Ka and Ks substitution rates of the syntenic gene pair, while the KaKs Calculator 2.0 determined Ka and Ks using the Nei-Gojobori (NG) method (Zhang et al. 2019).

### Plant material and treatment

*P. bretschneideri* Rehd. cv. 'Yali' and 'Dangshansuli' trees with comparable vigor and load, cultivated in an experimental orchard in Jiangsu province, served as research material. 'Yali' pear fruits were enclosed in triple-layer paper bags (red paper exterior, black paper middle layer, and white adhesive-bonded fabric interior) at 34 days after full bloom (34 DAFB), while unbagged 'Yali' and 'Dangshansuli' fruits in corresponding positions were marked. 'Yali' and 'Dangshansuli' represent typical pear cultivars in China; the bagging treatment of 'Yali' fruit is extensively practiced in Hebei province, China's largest pear production region, to enhance fruit appearance quality and satisfy international export market requirements.

The (un)bagged fruits were harvested at six developmental stages: fruit-setting stage (15 DAFB), physiological fruit drop stage (34 DAFB), approximately one month after fruit enlargement stage (81 DAFB), pre-mature stage (110 DAFB), mature stage (145 DAFB), and fruit senescence stage (160 DAFB) (Wang et al. 2021a, b). Following transportation to the laboratory, the pericarp and cortex tissues of 'Yali' fruit and the cortex tissue of 'Dangshansuli' fruit were collected for further analysis. The inclusion of 'Dangshansuli' fruit cortex aims to investigate whether the candidate gene functions in citrate metabolism in another cultivar of *P. bretschneideri* Rehd. Each treatment and/or cultivar included three biological replicates, with 20 fruits sampled per replicate at each developmental stage.

### Quantification of organic acids

Plant tissues, including pear cortex, pericarp, and calli, as well as tomato fruit, underwent homogenization with ultrapure water, followed by filtration through a 0.22-μm Millipore filter for supernatant collection.

Analysis of organic acids in the supernatant, including citrate, oxalate, tartarate, quininate, malate, and shikimate, was conducted using a high-performance liquid chromatography (HPLC) system (Thermo Ultimate 3000, Dionex, Massachusetts, USA) equipped with a Waters Acquity UPLC® HSS T3 column and a photodiode array (PDA) detector (Wang et al. 2018). Individual acids were qualified and quantified according to the method described by Wang et al. (2018). Total acid content was calculated as the sum of five individual acids.

### Determination of cytACO and mitACO activities

Cytosol and mitochondria extraction from various plant tissues, including pear cortex and calli as well as tomato fruit, followed the protocol of Matamoros et al. (2013). Subsequently, ACO activity in cytosol (cytACO) and mitochondria (mitACO) was measured using the corresponding assay kit (ACO-1-Z, Suzhou Comin Biotechnology Co., Ltd., Suzhou, China).

Protein concentration in the crude enzyme extract was determined using the bicinchoninic acid protein assay kit (A045-4, Nanjing Jiancheng Bioengineering Institute, Nanjing, China).

### Transcriptome and RT-qPCR analyses

Transcriptome analysis was conducted following the method of Li et al. (2019). In brief, total RNA was extracted from various pear tissues (including 15-DAFB fruit, stem, leaf, stigma, petal, ovary, pericarp, and cortex) using the EASYspin plant RNA extraction kit (Biomed, China). DNase (Takara Biotechnology Co., Ltd., Dalian, China) was applied to eliminate residual DNA before evaluating RNA integrity, concentration, and purity. The RNA-seq library was then constructed and sequenced on the Illumina HiSeq 2500 platform (Biomarker Technologies Co, Ltd., Beijing, China) (Li et al. 2019). Following quality assessment and data filtering, clean reads were mapped to the *P. bretschneideri* Rehd. genome (Wu et al., 2013) using HISAT2 software. Fragments per Kilobase Million (FPKM) was utilized to calculate gene expression, and differentially expressed genes (DEGs) were identified using DESeq2_EBSeq software, according to the following criteria: fold change ≥ 2.0 and false discovery rate (FDR) < 0.01 (Jia et al. 2023).

RT-qPCR assay was performed as described by Wang et al. (2018). Gene-specific primers were designed using Primer Premier 6.0 (Table S1). Total RNA from different plant tissues, including pear cortex and calli as well as tomato fruit, was isolated using TRIzol Reagents (Invitrogen, USA) followed by RNase-free DNase treatment (Qiagen, USA). Following first-strand cDNA synthesis, RT-qPCR assay was conducted using TaKaRa One Step SYBR® PrimeScript™ RT-PCR Kit (Perfect Real Time) (Takara Biotechnology Co., Ltd., Dalian, China) (Wang et al. 2018). *P. Tubulin* (*PbrTub*) served as the internal reference gene for the gene-overexpressing/silenced pear fruit and calli as well as during pear fruit development (Wang et al. 2018), while *S. lycopersicum Actin-51* (*SlActin-51*) served as the housekeeping gene for the gene-overexpressing tomato fruit (Liu et al. 2020). The relative gene expression level was calculated using the 2^-ΔΔCT^ method (Wang et al. 2018).

### Alignment of the coding sequences (CDSs), protein sequences, and promoter sequences from 'Yali' and/or 'Dangshansuli' fruits

The CDSs of *PbrACO2*, *PbrMYB3*, *PbrMYB65*, and *PbrNAC34a* were amplified from 'Yali' and 'Dangshansuli' fruits. The corresponding protein sequences were then deduced from their CDSs using the Translate database (https://web.expasy.org/translate/) (Jia et al. 2023). Additionally, the promoter sequences of *PbrMYB3* and *PbrMYB65* were amplified from 'Yali' fruit. Sequence alignment was conducted using DNAMAN software (Lynnon Biosoft, San Ramon, California, USA).

### Subcellular localization analyses

The CDSs of *PbrACO2*, *PbrMYB3*, *PbrMYB65*, *PbrNAC34a*, and *PbrWRKY72* without stop codons were amplified from 'Yali' fruit (Table S1), inserted into the pBI221 vector with a GFP tag, and subsequently co-transformed with the corresponding marker into *Arabidopsis* protoplasts (Jia et al. 2023). MSTP-mcherry (Sun et al. 2022) and AtH2B-mcherry (Liu et al. 2007) served as the mitochondrial and nuclear markers, respectively. The fluorescence signal was detected using a Leica TCS SP8 confocal laser-scanning microscope (Leica, Wetzlar, Germany).

### Functional validation of PbrACO2 in vitro

*PbrACO2* CDS was amplified from 'Yali' fruit (Table S1), inserted into the pCold-TF vector, and transformed into *E. coli* BL21 (DE3) for the expression of the His-tagged recombinant protein (Jia et al. 2023). Following purification through a Ni-NTA His bind resin column (Sangong Bioengineering Co., Ltd., Shanghai, China), the recombinant protein was collected for PbrACO2 activity assay as described above.

### Gene function validation in vivo

#### Transient transformation of pear fruit (a)Transient gene overexpression

*PbrACO2*, *PbrMYB3*, *PbrMYB65*, *PbrNAC34a*, and *PbrWRKY72* CDSs were isolated from'Yali'fruit (Table S1) and subsequently inserted into the pCAMBIA1300 vector containing a GFP tag. These constructs were then transformed into *A. tumefaciens* strain GV3101 and cultured at 28 °C until OD_600_ = 1.0. The bacterial strain was resuspended in infiltration buffer (10 mM magnesium chloride (MgCl_2_), 10 mM 2-morpholinoethanesulphonic acid (MES), and 150 μM acetosyringone), and 20 μL of the solution was carefully injected into the cortex tissue of developing'Yali'fruit before incubation at 25 °C for 3 d (Jia et al. 2023). Fruit infiltrated with the empty pCAMBIA1300 vector containing a GFP tag served as the control. (b)Transient gene silence Approximately 200-bp fragments of *PbrACO2*, *PbrMYB3*, *PbrMYB65 PbrNAC34a*, and *PbrWRKY72* CDSs were inserted into the pTRV2 vector (Table S1). The constructed pTRV2 and pTRV1 plasmids were transformed into *A. tumefaciens* strain GV3101, respectively; and suspended in infiltration buffer until OD_600_ = 1.0. Equal volumes of the recombinant pTRV2 and pTRV1 buffer were combined and carefully injected into cortex tissue of developing 'Yali' fruit prior to incubation at 25 °C for 3 d (Zhang et al. 2019). Fruits co-injected with empty pTRV2 and pTRV1 vectors served as controls.

#### Transformation of pear calli

*PbrACO2*, *PbrMYB3*, *PbrMYB65*, and *PbrNAC34a* CDSs were amplified and inserted into the pCAMBIA1300 vector containing a GFP tag as described above, then transformed into fruit calli derived from *P. communis* cv. 'Clapp's Favorite' pear fruitlet (Jia et al. 2023). Following selection on hygromycin B-containing MS medium (carbon source: sucrose), positive lines were verified at both the DNA level by PCR and the RNA level by RT-qPCR. The confirmed transgenic lines were transferred to hygromycin B-containing MS medium with mixed sugar (sucrose/sorbitol (1:1), 15 g L^−1^) as the carbon source to simulate fruit growing conditions (Jia et al. 2023). Calli transformed with the empty vector served as the control.

#### Transformation of tomato fruit

Previously constructed *PbrACO2*-/*PbrMYB65*-overexpressing vectors were transformed into *S. lycopersicum* cv. 'MicroTom' following the protocol of Cheng et al. (2018). Positive transgenic lines were selected on 100 mg L^−1^ kanamycin-containing medium and verified at both DNA level by PCR and RNA level by RT-qPCR. Plants were maintained in greenhouse conditions (18 h light at 25 ℃ and 6 h dark at 18 ℃, 60% relative humidity). Tomato fruits at 45 DAFB from control (wild-type) and transgenic homozygous lines (T2 generation) were collected for subsequent analysis.

#### Protein and DNA interaction analyses

**LUC assay** The CDSs of *PbrMYB3*, *PbrMYB65*, *PbrNAC34a*, and *PbrWRKY72* were isolated from'Yali'fruit and subsequently inserted into the pSAK277 vector. Additionally, promoter fragments of *PbrACO2*, *PbrMYB3*, and *PbrMYB65* of varying lengths, containing different quantities of potential PbrMYB3-/PbrMYB65-binding sites (MYBCORE box motif, CAACCG; *PbrACO2pro*, *PbrACO2pro*^*frag1*^, and *PbrACO2pro*^*frag2*^) or potential PbrNAC34a-binding sites (*PbrMYB3* promoter fragments included *PbrMYB3pro*, *PbrMYB3pro*^*frag1*^, *PbrMYB3pro*^*frag2*^, and *PbrMYB3pro*^*frag3*^; *PbrMYB65* promoter fragments included *PbrMYB65pro*, *PbrMYB65pro*^*frag1*^, *PbrMYB65pro*^*frag2*^, *PbrMYB65pro*^*frag3*^, and *PbrMYB65pro*^*frag4*^) as well as the mutated binding sites (CAACCG → CTTCCG in *PbrACO2* promoter, *PbrACO2pro*^*mut*^; CTTCGTTT → TTTTTTTT in *PbrMYB3* promoter, *PbrMYB3pro*^*mut*^; AGAAAGAA → CCCCCCCC in *PbrMYB65* promoter, *PbrMYB65pro*^*mut*^), were inserted into the pGreen 0800-LUC vector to generate various reporter constructs (Table S1). Subsequently, a combination of *A. tumefaciens* containing pSAK277-*PbrMYB3* (or pSAK277-*PbrMYB65*, or pSAK277-*PbrNAC34a*, or pSAK277-*PbrWRKY72*) and each reporter was infiltrated into *N. benthamiana* leaf (Liu et al., 2022). LUC and REN activities were measured using a dual-LUC reporter assay system (Promega Corporation, Madison, Wisconsin, USA). Transformants containing the empty pSAK277 vector and each reporter served as the controls.

**Y1H determination** The CDSs of *PbrMYB3, PbrMYB65*, and *PbrNAC34a* were amplified from'Yali'fruit and inserted into the prey vector pGADT7 (Table S1). The promoter fragments of *PbrACO2*, *PbrMYB3*, and *PbrMYB65*, approximately 200-bp in length, containing the PbrMYB3/65-binding sites (MYBCORE box motif, CAACCG; *PbrACO2pro*^*S1*^ and *PbrACO2pro*^*S2*^) or the PbrNAC34a-binding sites (CTTCGTTT in *PbrMYB3* promoter, *PbrMYB3pro*^*S1*^; AGAAAGAA in *PbrMYB*65 promoter, *PbrMYB65pro*^*S4*^) along with their mutated binding sites (CAACCG → CTTCCG in *PbrACO2* promoter fragments, *PbrACO2pro*^*S1 mut*^ and *PbrACO2pro*^*S2 mut*^; CTTCGTTT → TTTTTTTT in *PbrMYB3* promoter fragment, *PbrMYB3pro*^*S1 mut*^; AGAAAGAA → CCCCCCCC in *PbrMYB65* promoter fragment, *PbrMYB65pro*^*S4 mut*^), were cloned into the bait vector pAbAi (Table S1). The Y1H assay was performed using the Matchmaker Gold Yeast One-Hybrid Library Screening System (Shanghai Weidi Industrial Co., Ltd., Shanghai, China) (Liu et al. 2019). Self-activation was assessed using SD/-Ura medium supplemented with varying concentrations of AbA for *PbrACO2pro*^*S1/S2*^, *PbrACO2pro*^*S1 mut/S2 mut*^, *PbrMYB3pro*^*S1*^, *PbrMYB3pro*^*S1 mut*^, *PbrMYB65pro*^*S4*^, and *PbrMYB65pro*^*S4 mut*^ to determine optimal AbA concentration. The positive control comprised yeast cells co-transformed with pGADT7-*p53* and *p53*-pAbAi, while negative controls consisted of yeast cells co-transformed with empty pGADT7 vector and each bait.

**ChIP-qPCR analyses***PbrMYB3-/PbrMYB65*-/*PbrNAC34a*-overexpressing calli and control (empty vector), generated according to previously described methods, underwent DNA–protein cross-linking in 1% (v/v) formaldehyde. Following homogenization and cell lysis, the extracted chromatin was sonicated to obtain soluble sheared chromatin with DNA fragments ranging from 200–500 bp. A portion of the sheared chromatin served as input DNA, while the remainder underwent immunoprecipitation using anti-GFP antibody (Ab290, Abcam) (Li et al. 2018). The enrichment of *PbrACO2*, *PbrMYB3*, and *PbrMYB65* promoter fragments was quantified via qPCR assay (Table S1).

**EMSA assay** The His-tagged recombinant PbrMYB65 protein was produced following previously described methods (Table S1). FAM luciferase-labelled DNA probes approximately 30-bp in length, containing either wild-type (MYBCORE box motif, CAACCG) or mutated (CAACCG → CTTCCG) PbrMYB65-binding sites, and unlabeled competitor probes were synthesized by Sangong Bioengineering (Shanghai) Co., Ltd. The EMSA was conducted according to the protocol provided in the LightShift™ Chemiluminescent EMSA Kit (Thermo Fisher Scientific, Inc. Sunnyvale, California, USA) (Zhang et al., 2024).

#### PbrMYB3 and PbrMYB65 interaction determination

**Y2H assay** The CDSs of *PbrMYB3* and *PbrMYB65* were isolated from'Yali'fruit and subsequently inserted into the pGADT7 (AD) and pGBKT7 (BD) vectors (Clontech) (Table S1). The AD-*PbrMYB3*/*65* and BD-*PbrMYB3*/*65* constructs were co-transformed into *S. cerevisiae* AH109 and cultured on synthetic dropout nutrient media, including SD/-Leu/-Trp and SD/-Leu/-Trp/-His/-Ade (Ma et al. 2020). Control transformants included AD-*T* & BD-*53*, AD-*T* & BD-*Lam*, AD & BD, AD & BD-*PbrMYB3/65*, and AD-*PbrMYB3/65* & BD.

**BiFC analyses** The mature protein-encoding CDSs of *PbrMYB3* and *PbrMYB65* were amplified from'Yali'fruit (Table S1) and integrated into 35S-pSPYNE-YFP^N^ and 35S-pSPYCE-YFP^C^ vectors. The resulting constructs, *PbrMYB3/65*-YFP^N^ and *PbrMYB3/65*-YFP^C^, were introduced into *A. tumefaciens* strain GV3101 prior to infiltration into N. benthamiana leaf epidermal cells (Ma et al. 2020). Control transformants comprised YFP^N^ & YFP^C^, YFP^N^ & *PbrMYB3/65*-YFP^C^, and *PbrMYB3/65*-YFP^N^ & YFP^C^.

#### Homology modeling and molecular docking

AlphaFold2 was utilized to predict the protein structure of PbrACO2, while citrate's structure was transformed into a 3-D configuration using Gaussview through geometry optimization (B3LYP with the def2-TZVPP) (Jia et al. 2023). Molecular docking of PbrACO2 and citrate was performed using MOE-Dock (Vilar et al. 2008). The configuration exhibiting the highest binding score was selected and visualized using PyMOL software (Zhang et al., 2021).

#### Data analyses

The data presented represent the mean value of three biological replicates, with the exception of gene expression profiles during'Dangshansuli'fruit development (one replicate). Statistical analyses were conducted using SAS version 9.3 (SAS Institute, Cary, NC), particularly the analyses of variance (PROC ANOVA) with multi-comparison correction. Duncan's multiple range test at the 0.05 level was employed for mean separation. The Spearman correlation coefficient between attributes was calculated using R package, where correlations of 0.8–1.0 (absolute value) were considered extremely strong, and 0.6–0.8 (absolute value) were considered strong (Long et al. 2014).

## Supplementary Information

Additional file 1. Fig. S1 The schematic model on citrate metabolism in horticultural fruit. The schematic model was drawn based on the results of previous reports (Etienne et al. 2013; Tahjib-Ul-Arif et al. 2021). Fig. S2 Evolution of ACOs from 18 plant species. (a) Timescale tree of plant species. (b) Phylogenetic tree of plant ACOs. Information on 66 ACOs from 18 plant species is summarized in Table S2. Phylogenetic tree was constructed by the MEGA7.0 software, using the NJ method with the poisson model (Zhang et al. 2019); and all other settings were left as default. Different background colors represent distinct subgroups, and ACOs from different species were marked with different color lines. Fig. S3 Characteristics of ACOs from five horticultural plant species. (A) Chromosomal localization. Chromosome numbers are indicated on the inner side of the circle, and different color lines represent distinct chromosomes. Genes underwent WGD/segmental duplications are connected by red lines. (B) Phylogenetic tree. Different background colors represent distinct subgroups. Phylogenetic tree was constructed by the MEGA7.0 software, using the NJ method (Zhang et al. 2019). (C) *Cis*-acting element distribution. Boxes with distinct colors represent different *cis*-acting elements. (D) Gene structure. Yellow box represents the exon, blue box indicates the UTR, while black line represents the intron. (E) Motif distribution. Boxes with different colors represent the distinct motifs. Motifs composed the conserved domain are connected by dotted lines. Physiol-biochemical parameters of 25 ACOs from five horticultural plant species, including *Pyrus bretschneideri* (Pbr), *Prunus persica* (Ppe), *Musa acuminata* (Ma), *Vitis vinifera* (Vv), and *Actinidia chinensis* (Ac), are summarized in Table S3. Fig. S4 Comparative analyses of the 1.5 kb upstream of paralogous gene pairs. Divergence between upstream sequences of each paralogous gene pair was measured by the GATA program (Nix and Eisen 2005), with window size set at seven and a lower cutoff score of 12 bit. Solid dark line connects the similar region, while red broken line connects the matched region in reversed orientation. Fig. S5 Expression profiles of *PbrACOs*, *PbrMYB3, PbrMYB65*, and *PbrNAC34a* in six different tissues of ‘Yali’ pear. Six different tissues of ‘Yali’ pear included 15-DAFB fruit, stem, leaf, ovary, petal, and stigma. Data, adapted from transcriptome assay, represents the mean value of three biological replicates. The color scale represents the normalized log2-transformed (mean FPKM + 1), where red, blue, and white colors indicate high, low, and medium expression levels, respectively. On the other hand, the expression of *PbrACO6* is marked as gray (no expression). Fig. S6 Alternation in the expression profiles of *PbrACOs*, *PbrMYB3*, *PbrMYB65*, and *PbrNAC34a* after light exposure and temperature treatments. (A) Light exposure. ‘Yali’ fruit at 160 DAFB were storage in the dark (control) or exposed to light for 6 d. (B) Temperature treatments. Fruit at 160 DAFB were immersed in 0 ℃, 25 ℃ (control), or 53 ℃ water for 15 min. Data, adapted from transcriptome assay, represents the mean value of three biological replicates. The color scale represents normalized log2-transformed (mean FPKM + 1), where red, blue, and white colors indicate high, low, and medium expression levels, respectively. On the other hand, the expression of *PbrACO6* is marked as gray (no expression). Fig. S7 Dynamic change of organic acid percentage during ‘Yali’ fruit development. ‘Yali’ pear were bagged with triple-layer paper bags at 34 DAFB, while the unbagged fruit at the same positions were labelled as well. Pericarp and cortex tissues were sampled at six developmental stages, including 15 DAFB, 34 DAFB, 81 DAFB, 110 DAFB, 145 DAFB, and 160 DAFB. The content of total organic acid is set as 1.0. Data represents mean value ± SD of three biological replicates, and vertical bars labelled with the same letter are not significantly different between samples (*p* < 0.05). Fig. S8 Dynamic change of organic acid ratios during ‘Yali’ fruit development. ‘Yali’ pear were bagged with triple-layer paper bags at 34 DAFB, while the unbagged fruit at the same positions were labelled as well. Pericarp and cortex tissues were sampled at six developmental stages, including 15 DAFB, 34 DAFB, 81 DAFB, 110 DAFB, 145 DAFB, and 160 DAFB. Data represents mean value ± SD of three biological replicates, and vertical bars labelled with the same letter are not significantly different between samples (*p* < 0.05). Fig. S9 Dynamic change of citrate metabolism during ‘Dangshansuli’ fruit development. (A) Organic acid content. Data represents mean value ± SD of three biological replicates, and vertical bars labelled with the same letter are not significantly different between samples (*p* < 0.05). (B) cytACO and mitACO activities. Data represents mean value ± SD of three biological replicates, and vertical bars labelled with the same letter are not significantly different between samples (*p* < 0.05). (C) *PbrACOs* expression profiles. Data, adapted from transcriptome assay, represents the value of one biological replicates. The color scale represents normalized log2-transformed (FPKM + 1), where red, blue, and white colors indicate high, low, and medium expression levels, respectively; on the other hand, the expression of *PbrACO6* is marked as gray (no expression). (D) Correlations among attributes. Spearman correlation between attributes is visualized as a heatmap, where red color demonstrates a positive association, while blue color indicates a negative correlation. Cortex tissue of ‘Dangshansuli’ fruit was sampled at six developmental stages, including 15 DAFB, 34 DAFB, 81 DAFB, 110 DAFB, 145 DAFB, and 160 DAFB. Fig. S10 RT-qPCR validation of *PbrACOs* expression profiles during *P. bretschneideri* Rehd. fruit development. (A) ‘Yali’ fruit. (B) ‘Dangshansuli’ fruit. Cortex tissues of the unbagged ‘Yali’ and ‘Dangshansuli’ fruit were sampled at six developmental stages, including 15 DAFB, 34 DAFB, 81 DAFB, 110 DAFB, 145 DAFB, and 160 DAFB. Data represents mean value ± SD of three biological replicates for RT-qPCR result; and the expression level of *PbrACO2* in 15-DAFB ‘Dangshansuli’ fruit is set as 1.0. The pink bar and blue line represent RT-qPCR and transcriptome outcomes, respectively. Fig. S11 Alignment of gene CDS sequences from ‘Yali’ and ‘Dangshansuli’ fruit. (A) *PbrACO2*. (B) *PbrMYB3*. (C) *PbrMYB65*. (D) *PbrNAC34a*. Sequence alignment was performed using the DNAMAN software (Lynnon Biosoft, San Ramon, California, USA). Fig. S12 Alignment of protein sequences from ‘Yali’ and ‘Dangshansuli’ fruit. (A) PbrACO2. (B) PbrMYB3. (C) PbrMYB65. (D) PbrNAC34a. Sequence alignment was performed using the DNAMAN software (Lynnon Biosoft, San Ramon, California, USA). Fig. S13 3-D structure of PbrACO2 and citrate. (A) PbrACO2. (B) Citrate. PbrACO2 protein structure was predicted by AlphaFold2, while citrate structure was converted into a 3-D configuration in Gaussview through geometry optimization (B3LYP with the def2-TZVPP). Fig. S14 Impact of transient transformation of pear fruit with *PbrACO2* gene on citrate metabolism. (A) Transient overexpression of *PbrACO2*. (A-i) *PbrACO2* expression level and mitACO activity. (A-ii) Citrate content. ‘Yali’ fruit transformed with the empty pCAMBIA1300 vector containing a GFP tag was used as the control for the *PbrACO2*-overexpressing fruit. (B) Transient silence of *PbrACO2*. (B-i) *PbrACO2* expression level and mitACO activity. (B-ii) Citrate content. Fruit co-transformed with the empty TRV2 and TRV1 vectors was used as the control for the *PbrACO2*-silenced fruit. The expression level of *PbrACO2* in the control fruit is set as 1.0 for RT-qPCR assay. Data represents mean value ± SD of three biological replicates, and vertical bars labelled with the same small letter are not significantly different between samples (*p* < 0.05). Fig. S15 Gene function validation in tomato fruit. (A) Function validation of *PbrACO2* gene. (A-i) Phenotypes of the control (wide-type) and transgenic fruit. (A-ii) *PbrACO2* expression level and miACO activity. The expression level of *PbrACO2* in the *SlOE5*-fruit is set as 1.0 for RT-qPCR assay. (A-iii) Citrate content. (B) Function validation of *PbrMYB65* gene. (B-i) Phenotypes of the control (wide-type) and transgenic fruit. (B-ii) *PbrMYB65* expression level and miACO activity. The expression level of *PbrMYB65* in the *SlOE6*-fruit is set as 1.0 for RT-qPCR assay. (B-iii) Citrate content. Tomato fruit at 45 DAFB was sampled from the control (wide-type) and transgenic homozygous lines (T2 generation). Data represents mean value ± SD of three biological replicates, and vertical bars labelled with the same small letter are not significantly different between samples (*p* < 0.05). Fig. S16 Characterization of PbrMYB3 and PbrMYB65 as the possible up-restream regulators of *PbrACO2*. (A) Number of the differentially expressed TFs during ‘Yali’ fruit development. The differentially expressed TFs, whose expression levels in the pericarp tissue were consistently higher or lower than those in the cortex tissue of the developing ‘Yali’ fruit, were identified by DESeq2_EBSeq software, in accordance with the following criteria: fold change ≥ 2.0 and FRD < 0.01. (B) Expression profiles of the differentially expressed TFs during *P. bretschneideri* Rehd. fruit development and their correlations with *PbrACO2* mRNA abundance. The color scale represents normalized log2-transformed (FPKM + 1), where red, blue, and white colors indicate high, low, and medium expression levels, respectively. Spearman correlation between different attributes is visualized in the heatmap, where red (or light red) lines demonstrate extremely strong (or strong) positive correlations, while green (or light green) lines indicate extremely strong (or strong) negative associations. *PbrMYB3* (*Pbr003370.1*) is marked in red box and *PbrMYB65* (*Pbr000749.2*) is marked in blue box. (C) Detailed information of two PbrMYB3/65-binding sites in *PbrACO2* promoter. The possible binding sites (pink ellipses) of PbrMYB3/65 in *PbrACO2* promoter were predicted by the PlantRegMap database (Tian et al. 2020). ‘Yali’ pear fruit were bagged with triple-layer paper bags at 34 DAFB, while the unbagged ‘Yali’ and ‘Dangshansuli’ fruit at the same positions were labelled as well. Pericarp and cortex tissues were sampled at six developmental stages, including 15 DAFB, 34 DAFB, 81 DAFB, 110 DAFB, 145 DAFB, and 160 DAFB. Data, adapted from transcriptome assay, represent the mean value of three biological replicates, except for gene expression profiles during ‘Dangshansuli’ fruit development (one replicate). Fig. S17 Impact of transient transformation of pear fruit with *PbrMYB65* gene on citrate metabolism. (A) Transient overexpression of *PbrMYB65*. (A-i) Expression levels of *PbrMYB65* and *PbrACO2* genes. (A-ii) Citrate content. ‘Yali’ fruit transformed with the empty pCAMBIA1300 vector containing a GFP tag was used as the control for the *PbrMYB65*-overexpressing fruit. (B) Transient silence of *PbrMYB65*. (B-i) Expression levels of *PbrMYB65* and *PbrACO2* genes. (B-ii) Citrate content. Fruit co-transformed with the empty TRV2 and TRV1 vectors was used as the control for the *PbrMYB65*-silenced fruit. The expression level of *PbrMYB65* in the control fruit is set as 1.0 for RT-qPCR assay. Data represents mean value ± SD of three biological replicates, and vertical bars labelled with the same small letter are not significantly different between samples (*p* < 0.05). Fig. S18 PbrMYB3 self-interaction determination. (A) Y2H assay. Transformants containing AD-*T* & BD-*53*, AD-*T* & BD-*Lam*, AD & BD, AD & BD-*PbrMYB3*, and AD-*PbrMYB3* & BD were used as the controls. (B) BiFC analyses. Transformants containing YFP^N^ & YFP^C^, YFP^N^ & *PbrMYB3*-YFP^C^, and *PbrMYB3*-YFP^N^ & YFP^C^ were used as the controls. Bar, 20 μm. Fig. S19 Impact of transient transformation of pear fruit with *PbrMYB3* on citrate metabolism. (A) Transient overexpression of *PbrMYB3*. (A-i) Expression levels of *PbrMYB3* and *PbrACO2* genes. (A-ii) Citrate content. ‘Yali’ fruit transformed with the empty pCAMBIA1300 vector containing a GFP tag was used as the control for the *PbrMYB3*-overexpressing fruit. (B) Transient silence of *PbrMYB3*. (B-i) Expression levels of *PbrMYB3* and *PbrACO2* genes. (B-ii) Citrate content. Fruit co-transformed with the empty TRV2 and TRV1 vectors was used as the control for the *PbrMYB3*-silenced fruit. The expression level of *PbrMYB3* in the control fruit is set as 1.0 for RT-qPCR assay. Data represents mean value ± SD of three biological replicates, and vertical bars labelled with the same small letter are not significantly different between samples (*p* < 0.05). Fig. S20 Alignment of protein sequences and promoter sequences from ‘Yali’ fruit. (A) PbrMYB3 and PbrMYB65 protein sequences. (B) PbrMYB3 and PbrMYB65 promoter sequences. Sequence alignment was performed using the DNAMAN software (Lynnon Biosoft, San Ramon, California, USA). Fig. S21 Analyses of the relationship between PbrMYB3 and PbrMYB65. (A) Dual-luciferase assay of (A-i) the activation of *PbrMYB65* expression by PbrMYB3 as well as (A-ii) the activation of *PbrMYB3* expression by PbrMYB65. *PbrMYB3* and *PbrMYB65* CDSs were introduced into the pSAK277 vector, while their promoters into the pGreen 0800-LUC vector. Transformants containing the empty pSAK277 vector and each reporter were used as the controls. Data represents mean value ± SD of three biological replicates, and vertical bars labelled with the same letter are not significantly different between samples (*p* < 0.05). (B) Determination of the interaction between PbrMYB3 and PbrMYB65. (B-i) Y2H assay. Transformants containing AD-*T* & BD-*53*, AD-*T* & BD-*Lam*, AD & BD, AD & BD-*PbrMYB3*, and AD-*PbrMYB65* & BD were used as the controls. (B-ii) BiFC analyses. Transformants containing YFP^N^ & YFP^C^, YFP^N^ & *PbrMYB3*-YFP^C^, and *PbrMYB65*-YFP^N^ & YFP^C^ were used as the controls. Bar, 20 μm. Fig. S22 Correlations between *PbrMYB3*/*65* and 48 other differential expressed TFs during *P. bretschneideri* Rehd. fruit development. Information on *PbrMYB3* and *PbrMYB65* as well as 48 other differential expressed TFs are summarized in Table S12. The color scale represents normalized log2-transformed (FPKM + 1), where red, blue, and white colors indicate high, low, and medium expression levels, respectively. Spearman correlation between different attributes is visualized in the heatmap, where red (or light red) lines demonstrate extremely strong (or strong) positive correlations, while green (or light green) lines indicate extremely strong (or strong) negative associations. Data, adapted from transcriptome assay, represent the mean value of three biological replicates, except for gene expression profiles during ‘Dangshansuli’ fruit development (one replicate). *PbrNAC34a* (*Pbr026635.1*) is marked in red box, while *PbrWRKY72* (*Pbr009294.1*) in blue box. Fig. S23 Detailed information on the possible PbrNAC34a-binding sites in *PbrMYB3* and *PbrMYB65* promoters. (A) *PbrMYB3* promoter. (B) *PbrMYB65* promoter. The possible PbrNAC34a-binding sites in *PbrMYB3* (pink ellipses) and *PbrMYB65* (blue ellipses) promoters were characterized with the aid of the PlantRegMap database (Tian et al. 2020) as well as previous reports (Bi et al. 2023; Li et al. 2023). Fig. S24 Analyses of PbrWRKY72’s role in citrate metabolism. (A) Subcellular localization of PbrWRKY72. AtH2B-mcherry was used as the nuclear marker (Liu et al. 2007). Bar, 10 μm. (B) Dual-luciferase assay of the activation of *PbrMYB3* (B-i) and *PbrMYB65* (B-ii) expression by PbrWRKY72. *PbrWRKY72* CDS was introduced into the pSAK277 vector, while *PbrMYB3* and *PbrMYB65* promoters into the pGreen 0800-LUC vector. Transformants containing the empty pSAK277 vector and each reporter were used as the controls. Data represents mean value ± SD of three biological replicates, and vertical bars labelled with the same letter are not significantly different between samples (*p* < 0.05). (C) Impact of transient transformation of pear fruit with *PbrWRKY72* on citrate metabolism. (C-i) Transient overexpression of *PbrWRKY72*. ‘Yali’ fruit transformed with the empty pCAMBIA1300 vector containing a GFP tag was used as the control for the *PbrWRKY72*-overexpressing fruit. (C-ii) Transient silence of *PbrWRKY72*. Fruit co-transformed with the empty TRV2 and TRV1 vectors was used as the control for the *PbrWRKY72*-silenced fruit. The expression level of *PbrWRKY72* in the control fruit is set as 1.0 for RT-qPCR assay. Data represents mean value ± SD of three biological replicates, and vertical bars labelled with the same letter are not significantly different between samples (*p* < 0.05).

Additional file 2. Table S1 Primers used in this study. Table S2 Information on ACOs from 18 plant species. ACOs from *Arabidopsis* were used as queries in the BLASTP against plant genome databases before confirmation of the conserved domain by the SMART database (Terol et al. 2010; Wang et al. 2016; Wang et al. 2021a, b). Table S3 Physiol-biochemical parameters of ACOs from five horticultural plant species. Physio-biochemical parameters were calculated by the ProtParam tool (Wang et al. 2018). Table S4 Duplication types of *ACOs* from five horticultural plant species. A method similar to that used for the PGDD was applied to analyze the syntenic relationship, and the duplicated *ACOs* were categorized into the following types: WGD/segmental, tandem, singleton, proximal, and dispersed (Zhang et al. 2019). Table S5 Motif sequences in ACOs from five horticultural plant species. Motifs were characterized by MEME Suite tool (Bailey et al. 2015). Table S6 Expression profiles of *PbrACOs*, *PbrMYB3*, *PbrMYB65*, and *PbrNAC34a* in different tissues of ‘Yali’ pear. Six different tissues of ‘Yali’ fruit included 15-DAFB fruit, stem, leaf, ovary, petal, and stigma. Data, adapted from transcriptome assay, represents the mean value of three biological replicates. Table S7 Alternation in the expression profiles of *PbrACOs*, *PbrMYB3, PbrMYB65*, and *PbrNAC34a* after light exposure. ‘Yali’ fruit at 160 DAFB were storage in the dark (control) or exposed to light for 6 d. Data, adapted from transcriptome assay, represents the mean value of three biological replicates. Table S8 Alternation in the expression profiles of *PbrACOs*, *PbrMYB3*, *PbrMYB65*, and *PbrNAC34a* after temperature treatments. ‘Yali’ fruit at 160 DAFB were immersed in 0 ℃, 25 ℃ (control), or 53 ℃ water for 15 min. Data, adapted from transcriptome assay, represents the mean value of three biological replicates. Table S9 Dynamic change of organic acid content during *P. bretschneideri* Rehd. fruit development. ‘Yali’ pear fruit were bagged with triple-layer paper bags at 34 DAFB, while the unbagged ‘Yali’ and ‘Dangshansuli’ fruit at the same positions were labelled as well. Pericarp and cortex tissues were sampled at six developmental stages, including 15 DAFB, 34 DAFB, 81 DAFB, 110 DAFB, 145 DAFB, and 160 DAFB. Data represents the mean value of three biological replicates. Table S10 Expression profiles of *PbrACOs* during *P. bretschneideri* Rehd. fruit development. ‘Yali’ pear fruit were bagged with triple-layer paper bags at 34 DAFB, while the unbagged ‘Yali’ and ‘Dangshansuli’ fruit at the same positions were labelled as well. Pericarp and cortex tissues were sampled at six developmental stages, including 15 DAFB, 34 DAFB, 81 DAFB, 110 DAFB, 145 DAFB, and 160 DAFB. Data, adapted from transcriptome assay, represents the mean value of three biological replicates, except for gene expression profiles during ‘Dangshansuli’ fruit development (one replicate). Table S11 The list of amino acid residues involved in the interaction between PbrACO2 and citrate. MOE-Dock was used for the molecular docking of PbrACO2 and citrate (Vilar et al. 2008), and the configuration with the highest binding score was selected and then visualized with PyMOL software (Zhang et al., 2021). Table S12 Expression profiles of the differentially expressed TFs during *P. bretschneideri* Rehd. fruit development. ‘Yali’ pear fruit were bagged with triple-layer paper bags at 34 DAFB, while the unbagged ‘Yali’ and ‘Dangshansuli’ fruit at the same positions were labelled as well. Pericarp and cortex tissues were sampled at six developmental stages, including 15 DAFB, 34 DAFB, 81 DAFB, 110 DAFB, 145 DAFB, and 160 DAFB. The differentially expressed TFs, whose expression levels in the pericarp tissue were consistently higher or lower than those in the cortex tissue of the developing ‘Yali’ fruit, were identified by DESeq2_EBSeq software, in accordance with the following criteria: fold change ≥ 2.0 and FRD < 0.01. Data, adapted from transcriptome assay, represents the mean value of three biological replicates, except for gene expression profiles during ‘Dangshansuli’ fruit development (one replicate).

## Data Availability

Transcriptome assay was conducted with the aid of Biomarker Technologies Co, Ltd. (Beijing, China). The raw sequence data reported in this paper have been deposited in the Genome Sequence Archive (GSA) in National Genomics Data Center (accession number: CRA011265 and CRA011138). All metabolome and transcriptome data, which were generated or analyzed during this study, were included in this published article and its supplementary information files (Table S6-S10 & S12). Moreover, all other data are available from the corresponding author upon reasonable request.

## Abbreviations

ACO: Aconitase
cytACO: Cytosolic ACO
mitACO: Mitochondrial ACO
ALMT: Aluminum-activated malate transporter
AbA: Aureobasidin A
BiFC: Bimolecular fluorescence complementation
ChIP-qPCR: Chromatin immunoprecipitation-quantitative PCR
CS: Citrate synthase
CDS: Coding sequence
CytACO: Cytosolic aconitase
DAFB: Days after full bloom
DEG: Differentially expressed gene
EMSA: Electrophoretic mobility shift assay
FDR: False discovery rate
FPKM: Fragments per Kilobase Million
HPLC: High-performance liquid chromatography
IDH: Isocitrate dehydrogenase
LUC: Luciferase
MgCl_2_: Magnesium chloride
mitACO: Mitochondrial aconitase
MES: 2-Morpholinoethanesulphonic acid
MS: Murashige and Skoog
NAD^+^-IDH: NAD^+^-dependent isocitrate dehydrogenase
NADP^+^-IDH: NADP^+^-dependent isocitrate dehydrogenase
NG: Nei-Gojobori: NG;
NJ: Neighbor-Joining
OAA: Oxaloacetate
AD: PGADT7
BD: PGBKT7
PEP: Phosphoenolpyruvate
PEPC: Phosphoenolpyruvate carboxylase
PDA: Photodiode array
PGDD: Plant Genome Duplication Database
RT-qPCR: Real-time quantitative polymerase chain reaction
REN: Renilla
SWEET: Sugars Will Eventually be Exported Transporter
TF: Transcription factor
TCA: Tricarboxylic acid
WGD: Whole genome duplication
Y1H: Yeast one-hybrid
Y2H: Yeast two-hybrid
