# Supplementary material for: PbrNAC34a- PbrMYB3/65- PbrACO2 cascade plays a role in citrate difference between the pericarp and cortex tissues of pear (P. bretschneideri Rehd.) fruit
Source: Mol Hortic. 2025 Oct 10;5:55. doi: 10.1186/s43897-025-00177-9 (PMC12512694; doi:10.1186/s43897-025-00177-9)
Supplement: Supplementary file 1 — Additional file 1. Fig. S1 The schematic model on citrate metabolism in horticultural fruit. The schematic model was drawn based on the results of previous reports (Etienne et al. 2013; Tahjib-Ul-Arif et al. 2021). Fig. S2 Evolution of ACOs from 18 plant species. (a) Timescale tree of plant species. (b) Phylogenetic tree of plant ACOs. Information on 66 ACOs from 18 plant species is summarized in Table S2. Phylogenetic tree was constructed by the MEGA7.0 software, using the NJ method with the poisson model (Zhang et al. 2019); and all other settings were left as default. Different background colors represent distinct subgroups, and ACOs from different species were marked with different color lines. Fig. S3 Characteristics of ACOs from five horticultural plant species. (A) Chromosomal localization. Chromosome numbers are indicated on the inner side of the circle, and different color lines represent distinct chromosomes. Genes underwent WGD/segmental duplications are connected by red lines. (B) Phylogenetic tree. Different background colors represent distinct subgroups. Phylogenetic tree was constructed by the MEGA7.0 software, using the NJ method (Zhang et al. 2019). (C) Cis-acting element distribution. Boxes with distinct colors represent different cis-acting elements. (D) Gene structure. Yellow box represents the exon, blue box indicates the UTR, while black line represents the intron. (E) Motif distribution. Boxes with different colors represent the distinct motifs. Motifs composed the conserved domain are connected by dotted lines. Physiol-biochemical parameters of 25 ACOs from five horticultural plant species, including Pyrus bretschneideri (Pbr), Prunus persica (Ppe), Musa acuminata (Ma), Vitis vinifera (Vv), and Actinidia chinensis (Ac), are summarized in Table S3. Fig. S4 Comparative analyses of the 1.5 kb upstream of paralogous gene pairs. Divergence between upstream sequences of each paralogous gene pair was measured by the GATA program (Nix and Eisen 2005) [file 43897_2025_177_MOESM1_ESM.docx]

**Supplemental Figures**


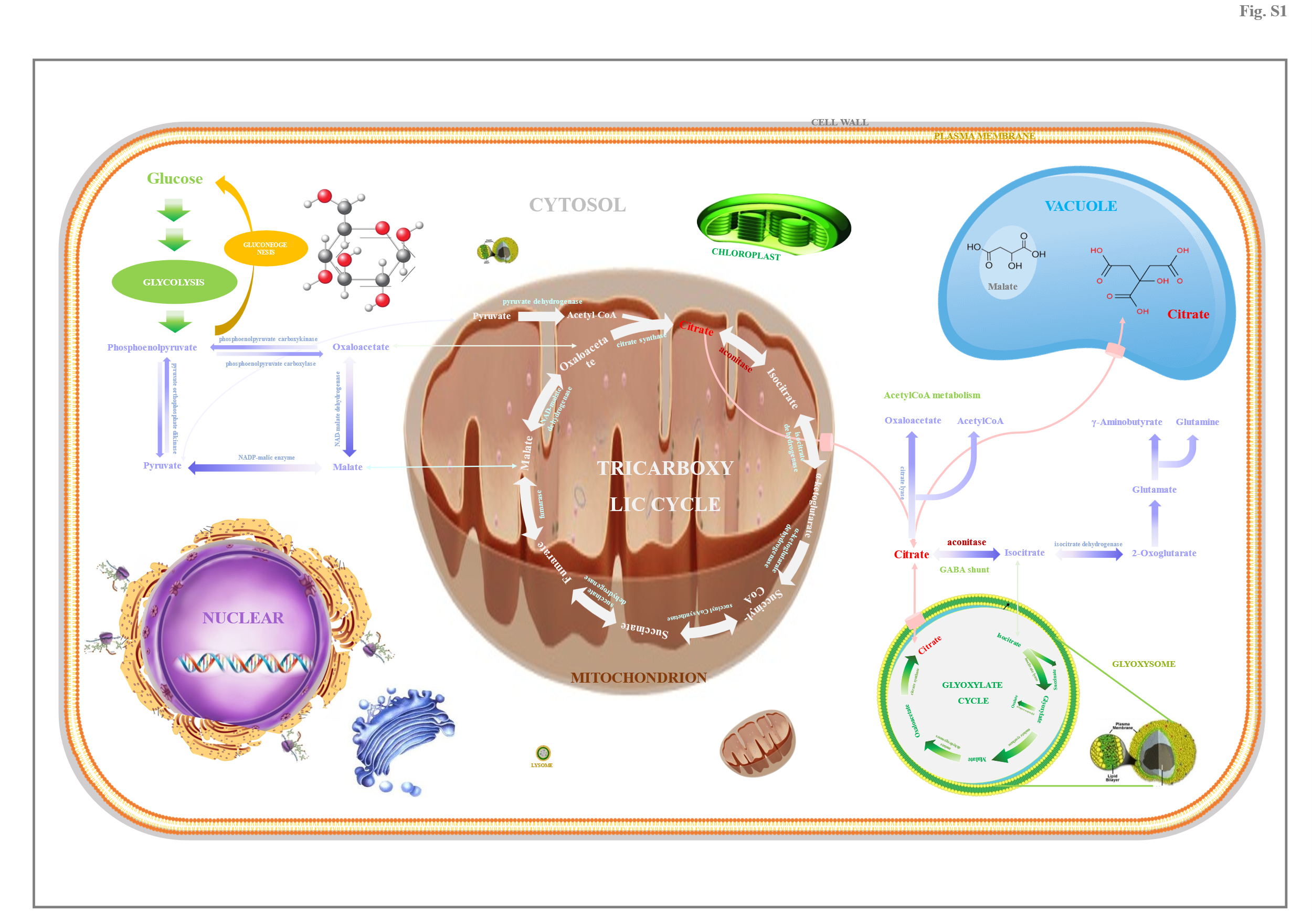


**Fig. S1. The schematic model on citrate metabolism in horticultural fruit.** The schematic model was drawn based on the results of previous reports (Etienne et al., 2013; Tahjib-Ul-Arif et al., 2021).


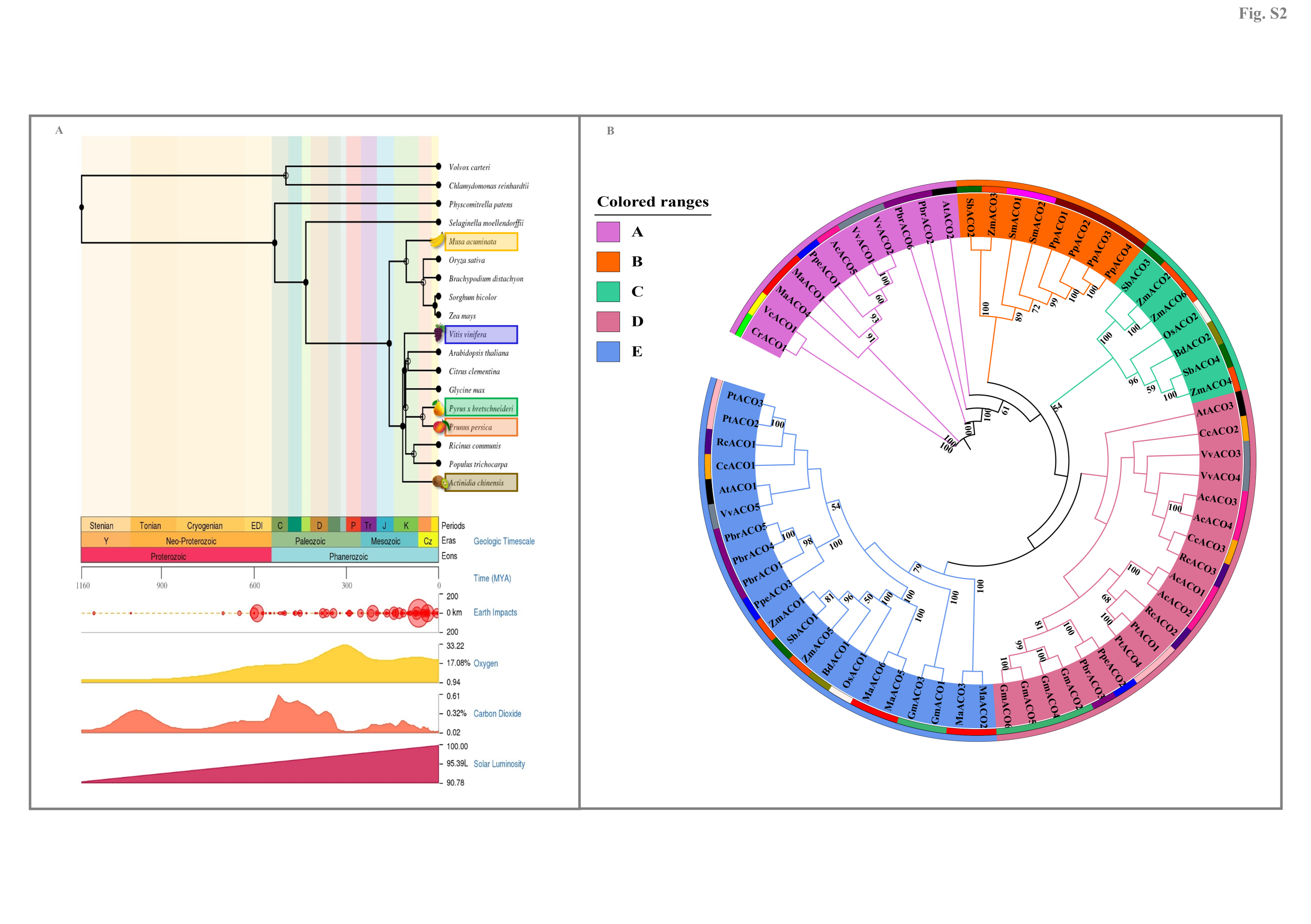


**Fig. S2. Evolution of ACOs from 18 plant species.** **(a) Timescale tree of plant species. (b) Phylogenetic tree of plant ACOs.** Information on 66 ACOs from 18 plant species is summarized in Table S2. Phylogenetic tree was constructed by the MEGA7.0 software, using the NJ method with the poisson model (Zhang et al., 2019); and all other settings were left as default. Different background colors represent distinct subgroups, and ACOs from different species were marked with different color lines.


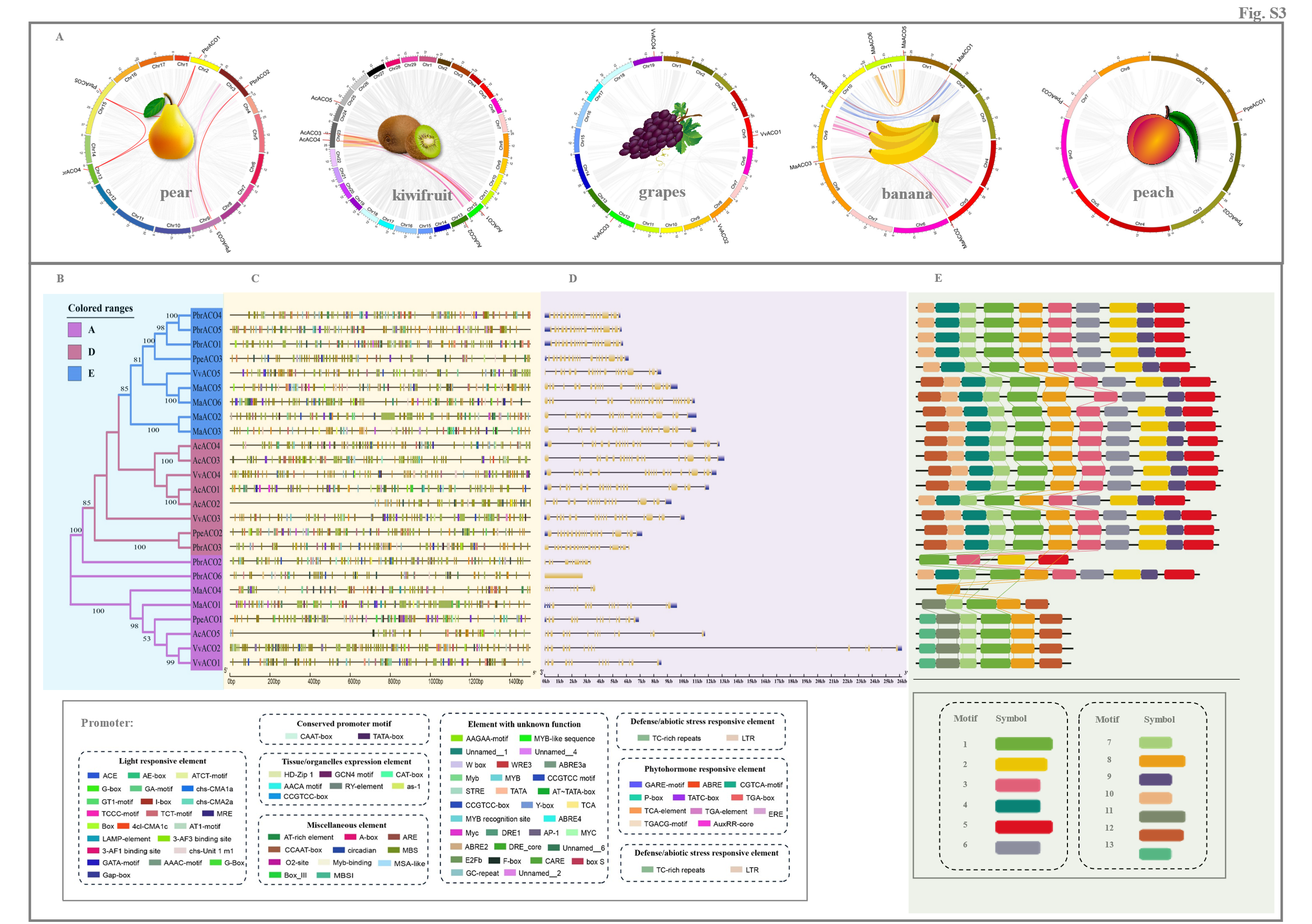


**Fig. S3. Characteristics of ACOs from five horticultural plant species. (A) Chromosomal localization.** Chromosome numbers are indicated on the inner side of the circle, and different color lines represent distinct chromosomes. Genes underwent WGD/segmental duplications are connected by red lines. **(B) Phylogenetic tree.** Different background colors represent distinct subgroups. Phylogenetic tree was constructed by the MEGA7.0 software, using the NJ method (Zhang et al., 2019). **(C) *Cis*-acting element distribution.** Boxes with distinct colors represent different *cis*-acting elements. **(D) Gene structure.** Yellow box represents the exon, blue box indicates the UTR, while black line represents the intron. **(E) Motif distribution.** Boxes with different colors represent the distinct motifs. Motifs composed the conserved domain are connected by dotted lines. Physiol-biochemical parameters of 25 ACOs from five horticultural plant species, including *Pyrus bretschneideri* (Pbr), *Prunus persica* (Ppe), *Musa acuminata* (Ma), *Vitis vinifera* (Vv), and *Actinidia chinensis* (Ac), are summarized in Table S3.


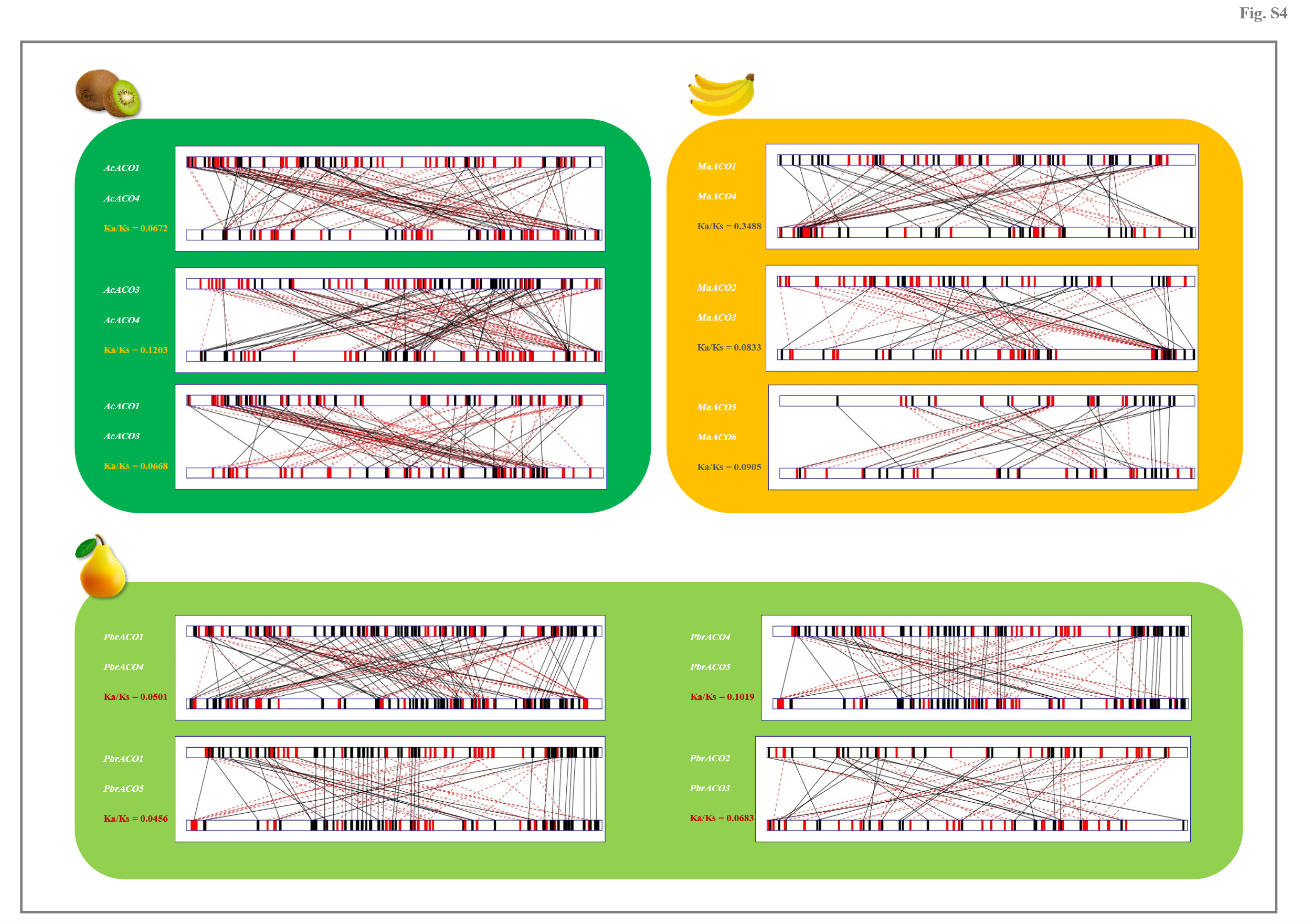


**Fig. S4. Comparative analyses of the 1.5 kb upstream of paralogous gene pairs.** Divergence between upstream sequences of each paralogous gene pair was measured by the GATA program (Nix and Eisen, 2005), with window size set at seven and a lower cutoff score of 12 bit. Solid dark line connects the similar region, while red broken line connects the matched region in reversed orientation.

**
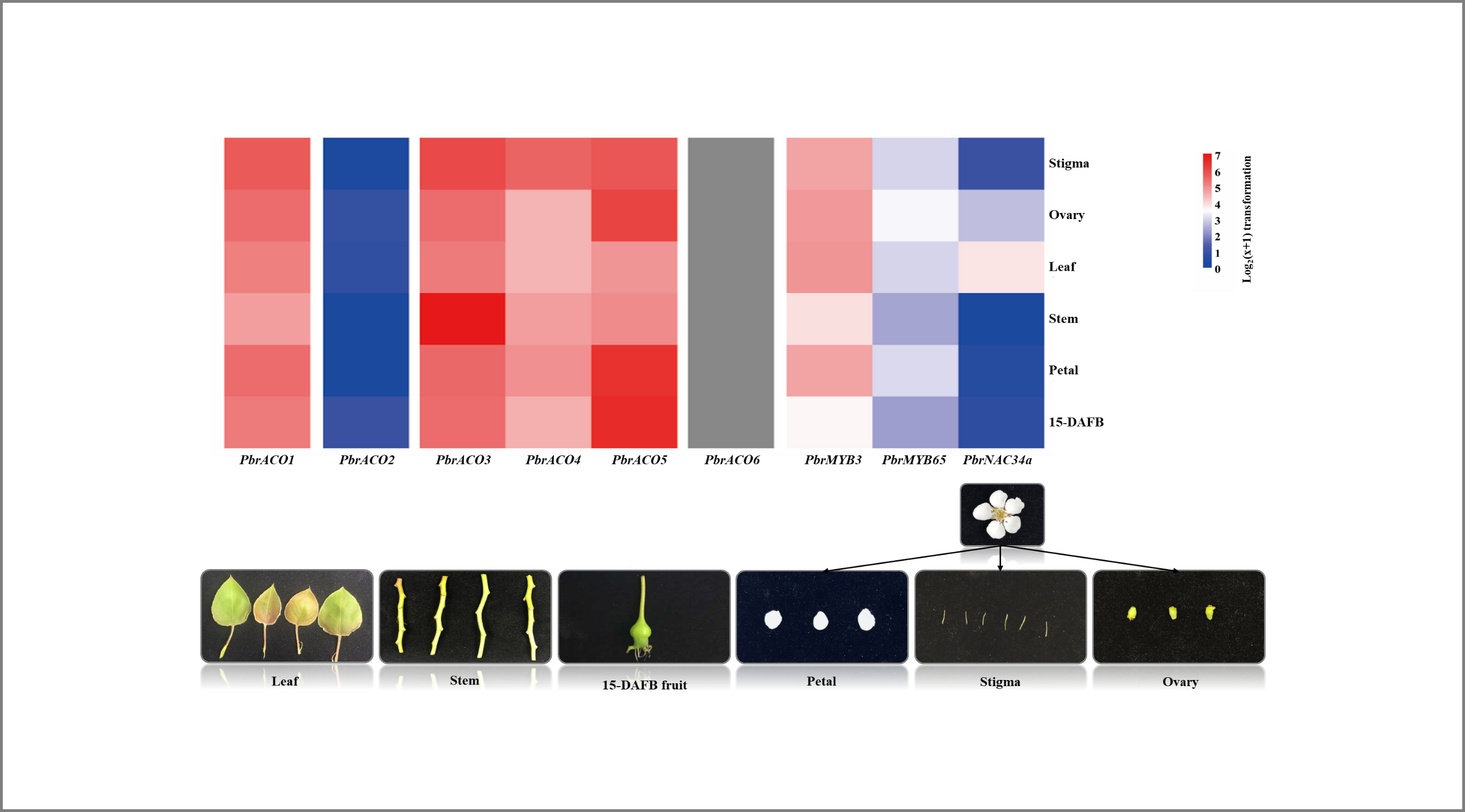
**

**Fig. S5. Expression profiles of *PbrACOs*, *PbrMYB3, PbrMYB65*, and *PbrNAC34a* in six different tissues of ‘Yali’ pear.** Six different tissues of ‘Yali’ pear included 15-DAFB fruit, stem, leaf, ovary, petal, and stigma. Data, adapted from transcriptome assay, represents the mean value of three biological replicates. The color scale represents the normalized log2-transformed (mean FPKM + 1), where red, blue, and white colors indicate high, low, and medium expression levels, respectively. On the other hand, the expression of *PbrACO6* is marked as gray (no expression).


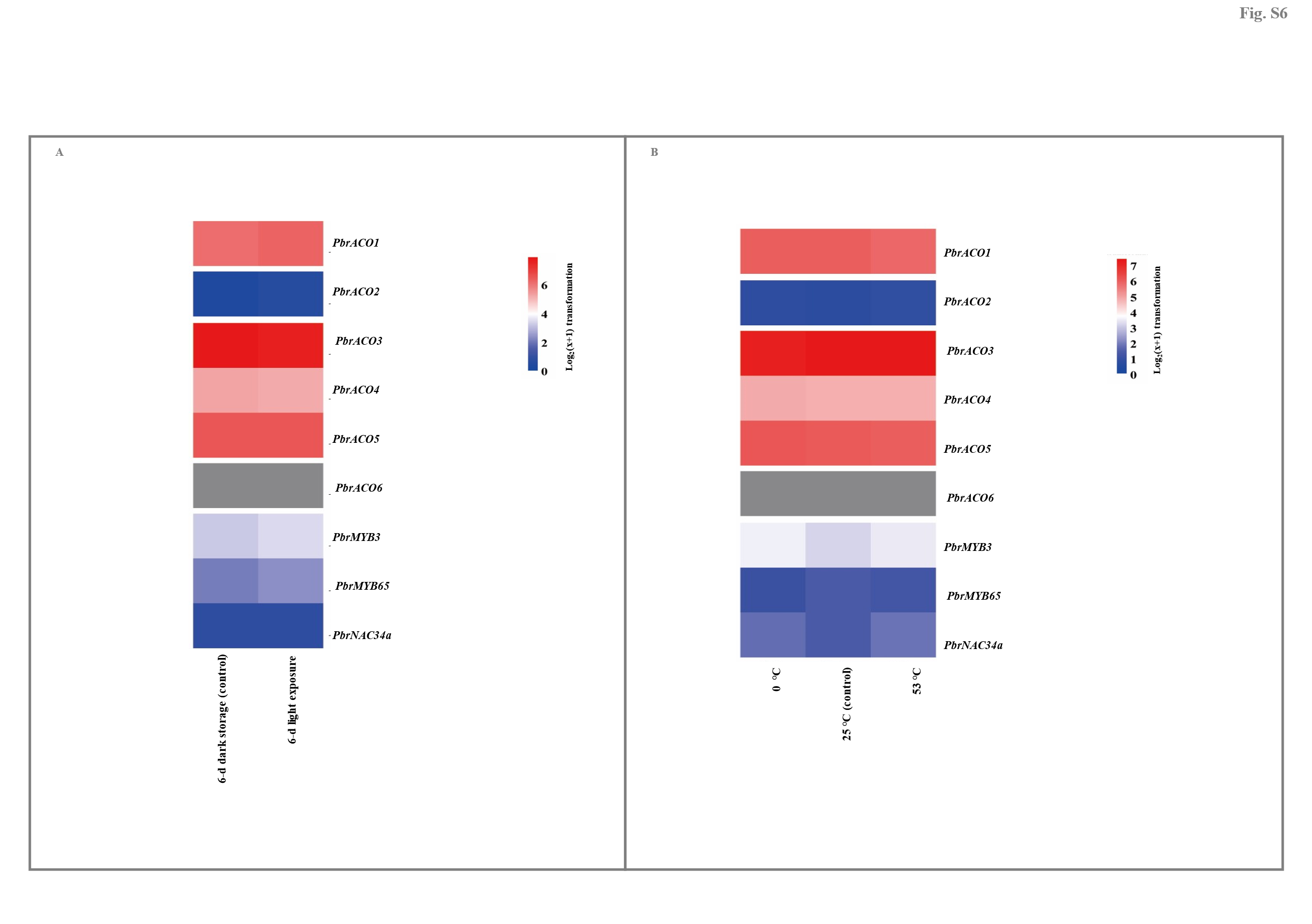


**Fig. S6. Alternation in the expression profiles of *PbrACOs*, *PbrMYB3*, *PbrMYB65*, and *PbrNAC34a* after light exposure and temperature treatments. (A) Light exposure.** ‘Yali’ fruit at 160 DAFB were storage in the dark (control) or exposed to light for 6 d. **(B) Temperature treatments.** Fruit at 160 DAFB were immersed in 0 ℃, 25 ℃ (control), or 53 ℃ water for 15 min. Data, adapted from transcriptome assay, represents the mean value of three biological replicates. The color scale represents normalized log2-transformed (mean FPKM + 1), where red, blue, and white colors indicate high, low, and medium expression levels, respectively. On the other hand, the expression of *PbrACO6* is marked as gray (no expression).


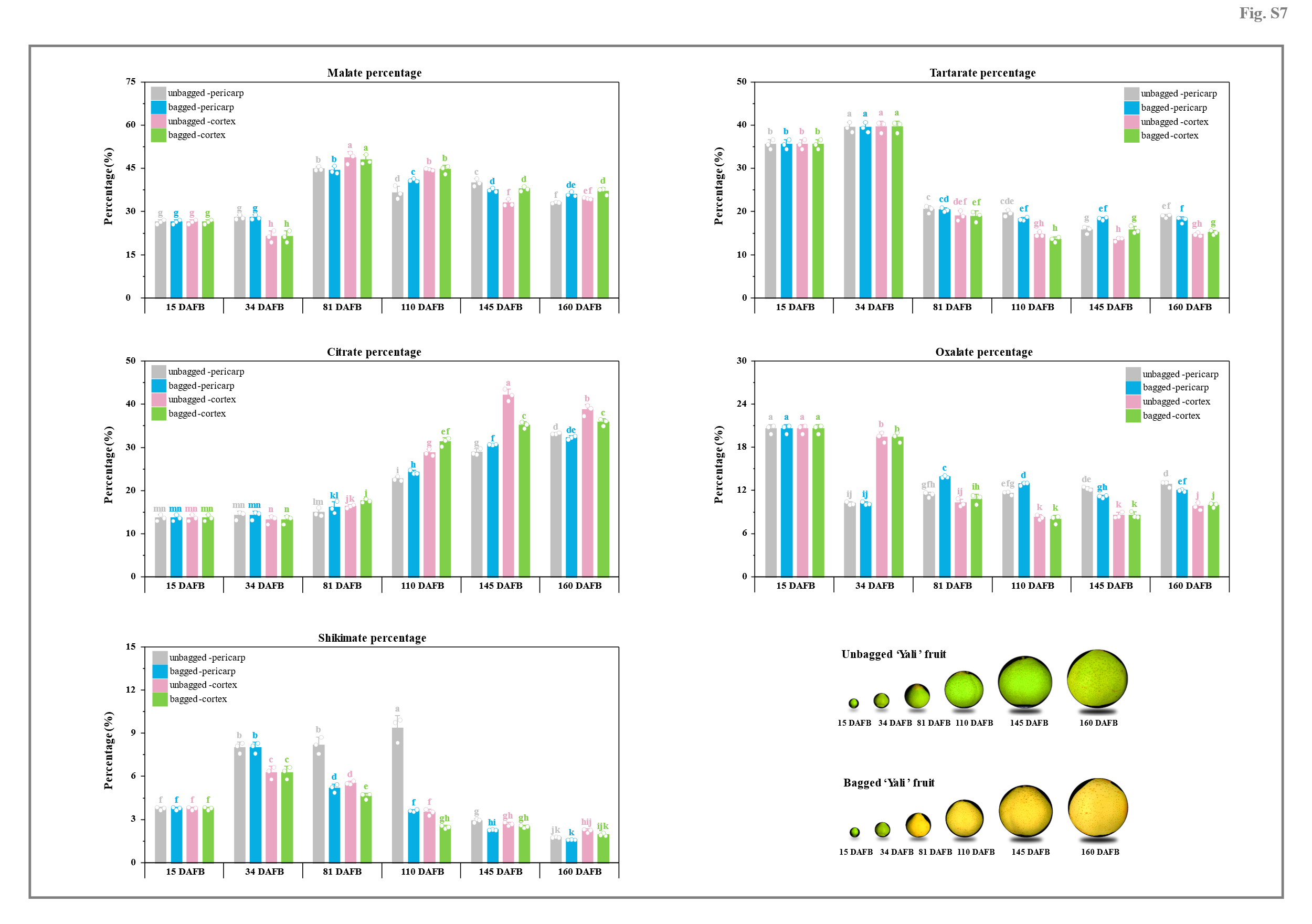


**Fig. S7. Dynamic change of organic acid percentage during ‘Yali’ fruit development.** ‘Yali’ pear were bagged with triple-layer paper bags at 34 DAFB, while the unbagged fruit at the same positions were labelled as well. Pericarp and cortex tissues were sampled at six developmental stages, including 15 DAFB, 34 DAFB, 81 DAFB, 110 DAFB, 145 DAFB, and 160 DAFB. The content of total organic acid is set as 1.0. Data represents mean value ± SD of three biological replicates, and vertical bars labelled with the same letter are not significantly different between samples (*p* < 0.05).


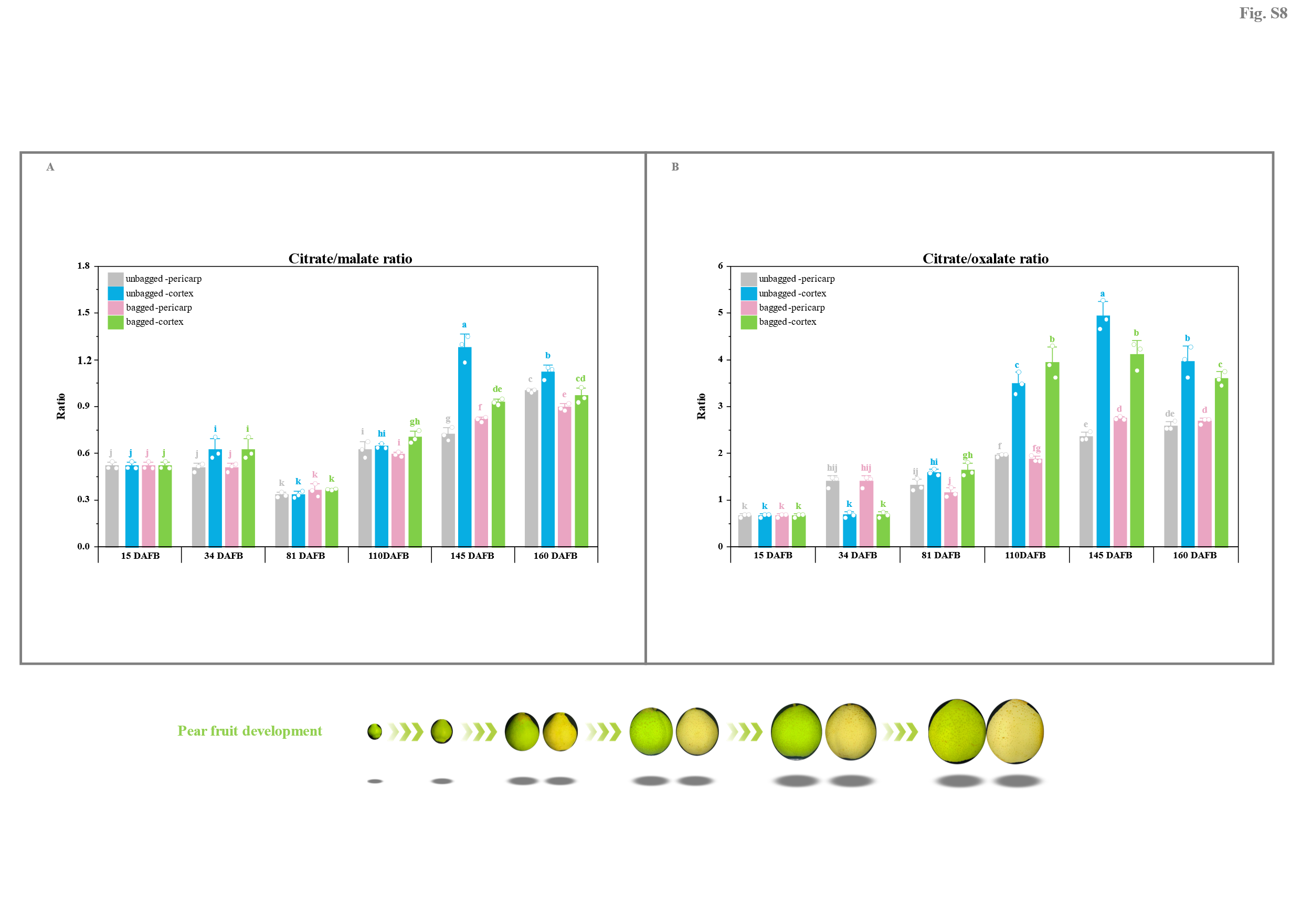


**Fig. S8. Dynamic change of organic acid ratios during ‘Yali’ fruit development.** ‘Yali’ pear were bagged with triple-layer paper bags at 34 DAFB, while the unbagged fruit at the same positions were labelled as well. Pericarp and cortex tissues were sampled at six developmental stages, including 15 DAFB, 34 DAFB, 81 DAFB, 110 DAFB, 145 DAFB, and 160 DAFB. Data represents mean value ± SD of three biological replicates, and vertical bars labelled with the same letter are not significantly different between samples (*p* < 0.05).


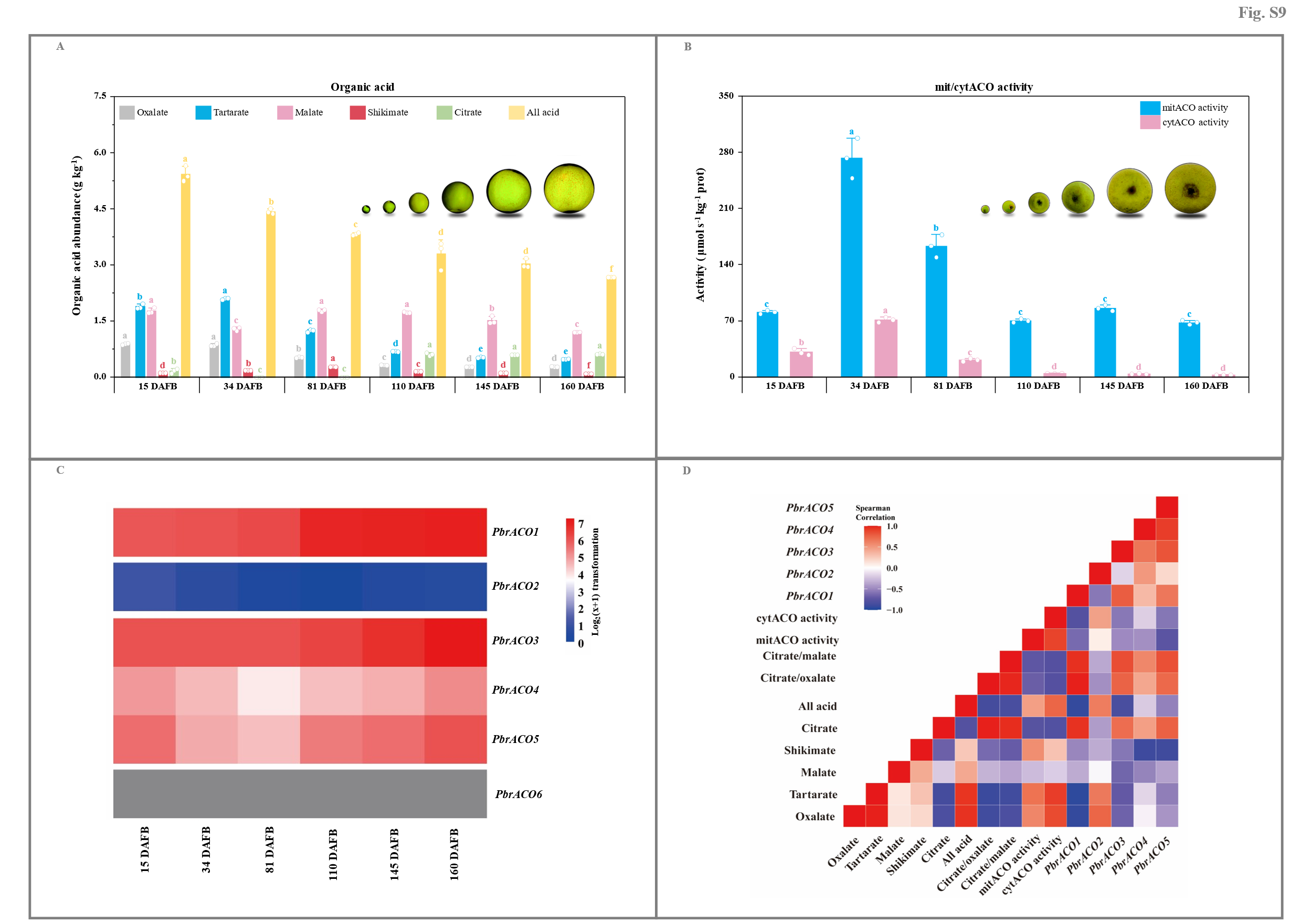


**Fig. S9. Dynamic change of citrate metabolism during ‘Dangshansuli’ fruit development. (A) Organic acid content.** Data represents mean value ± SD of three biological replicates, and vertical bars labelled with the same letter are not significantly different between samples (*p* < 0.05). **(B) cytACO and mitACO activities.** Data represents mean value ± SD of three biological replicates, and vertical bars labelled with the same letter are not significantly different between samples (*p* < 0.05). **(C) *PbrACOs* expression profiles.** Data, adapted from transcriptome assay, represents the value of one biological replicates. The color scale represents normalized log2-transformed (FPKM + 1), where red, blue, and white colors indicate high, low, and medium expression levels, respectively; on the other hand, the expression of *PbrACO6* is marked as gray (no expression). **(D) Correlations among attributes.** Spearman correlation between attributes is visualized as a heatmap, where red color demonstrates a positive association, while blue color indicates a negative correlation. Cortex tissue of ‘Dangshansuli’ fruit was sampled at six developmental stages, including 15 DAFB, 34 DAFB, 81 DAFB, 110 DAFB, 145 DAFB, and 160 DAFB.


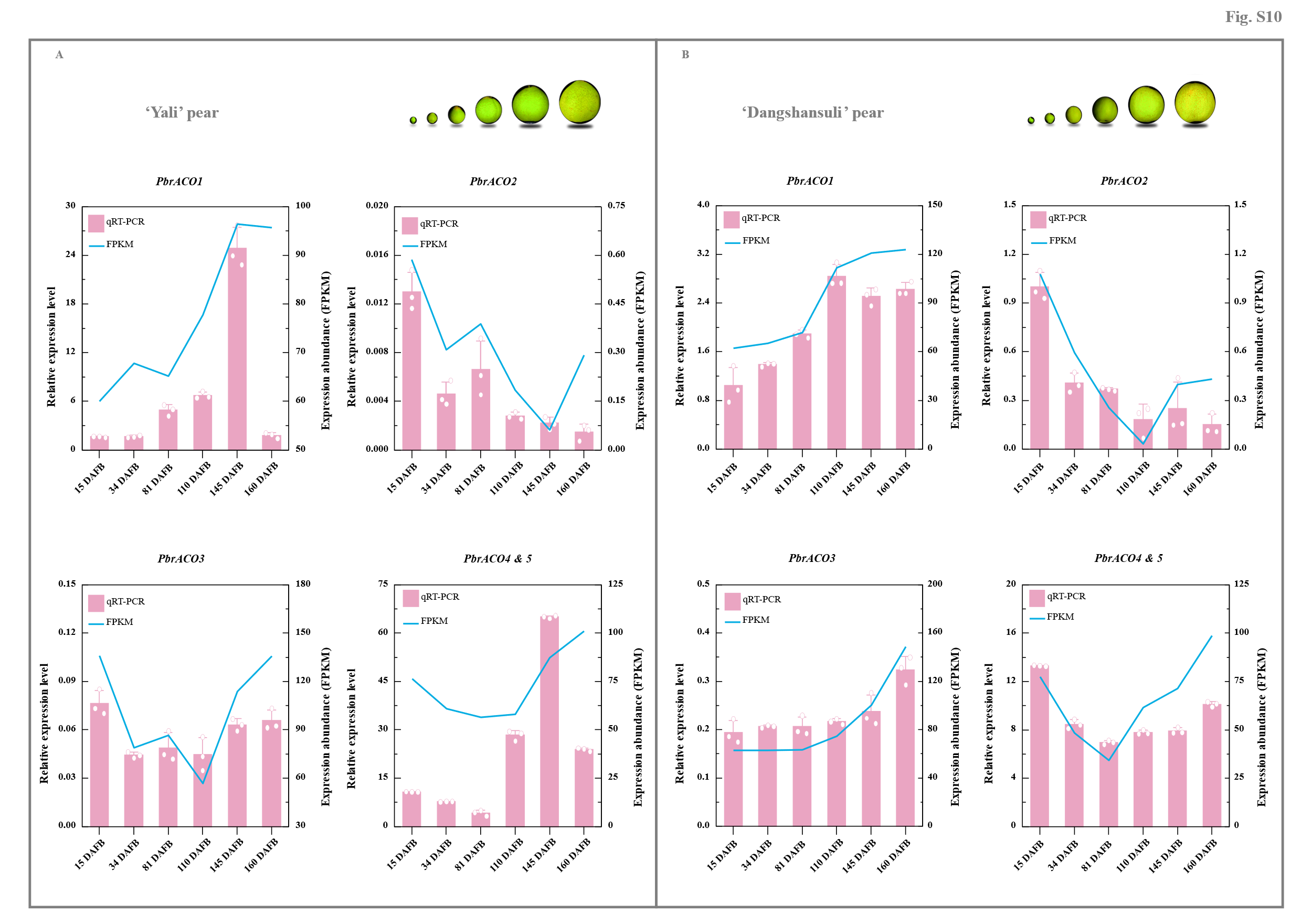


**Fig. S10. RT-qPCR validation of *PbrACOs* expression profiles during *P. bretschneideri* Rehd. fruit development. (A) ‘Yali’ fruit. (B) ‘Dangshansuli’ fruit.** Cortex tissues of the unbagged ‘Yali’ and ‘Dangshansuli’ fruit were sampled at six developmental stages, including 15 DAFB, 34 DAFB, 81 DAFB, 110 DAFB, 145 DAFB, and 160 DAFB. Data represents mean value ± SD of three biological replicates for RT-qPCR result; and the expression level of *PbrACO2* in 15-DAFB ‘Dangshansuli’ fruit is set as 1.0. The pink bar and blue line represent RT-qPCR and transcriptome outcomes, respectively.


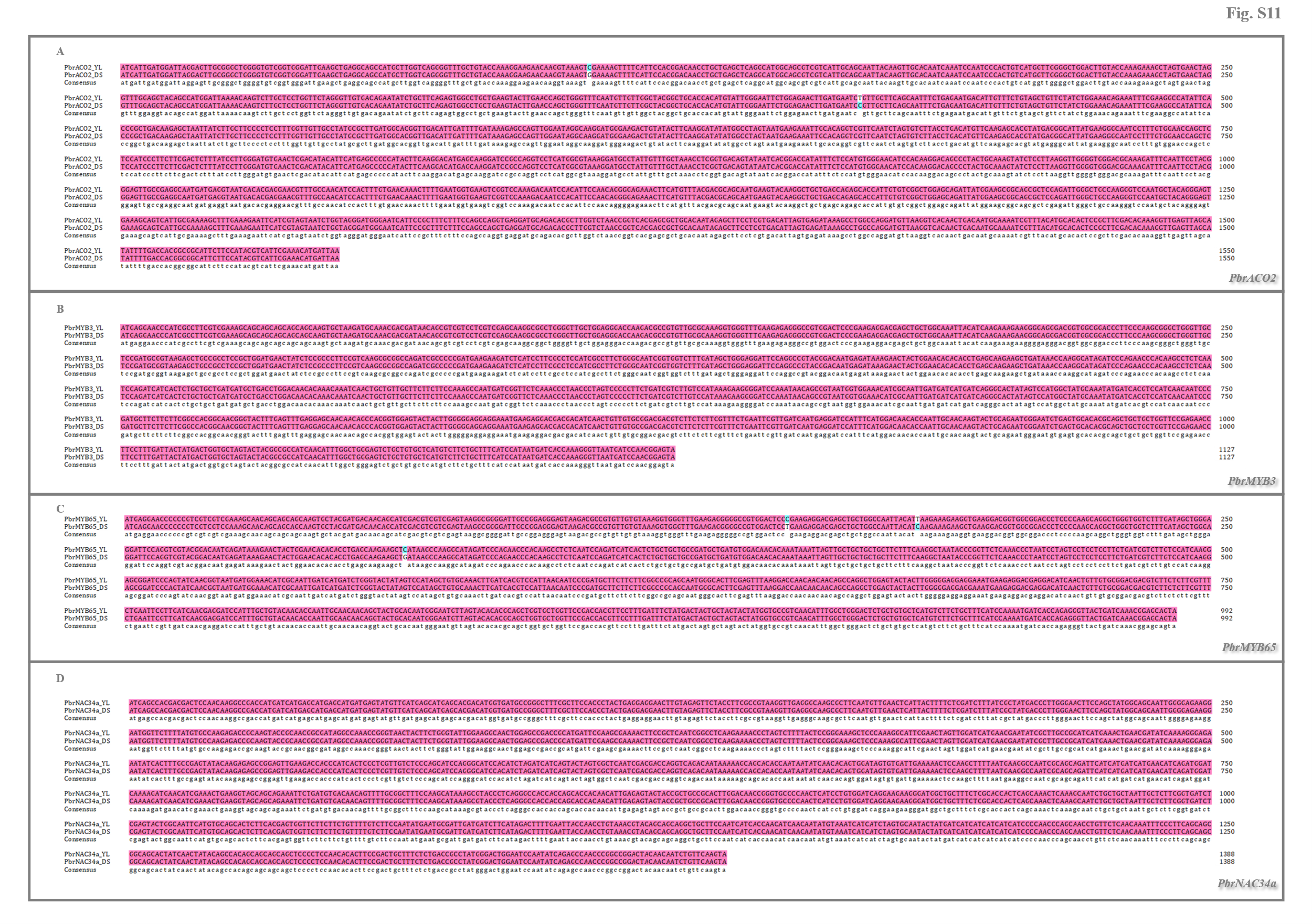


**Fig. S11. Alignment of gene CDS sequences from ‘Yali’ and ‘Dangshansuli’ fruit. (A) *PbrACO2*. (B) *PbrMYB3*. (C) *PbrMYB65*. (D) *PbrNAC34a*.** Sequence alignment was performed using the DNAMAN software (Lynnon Biosoft, San Ramon, California, USA).


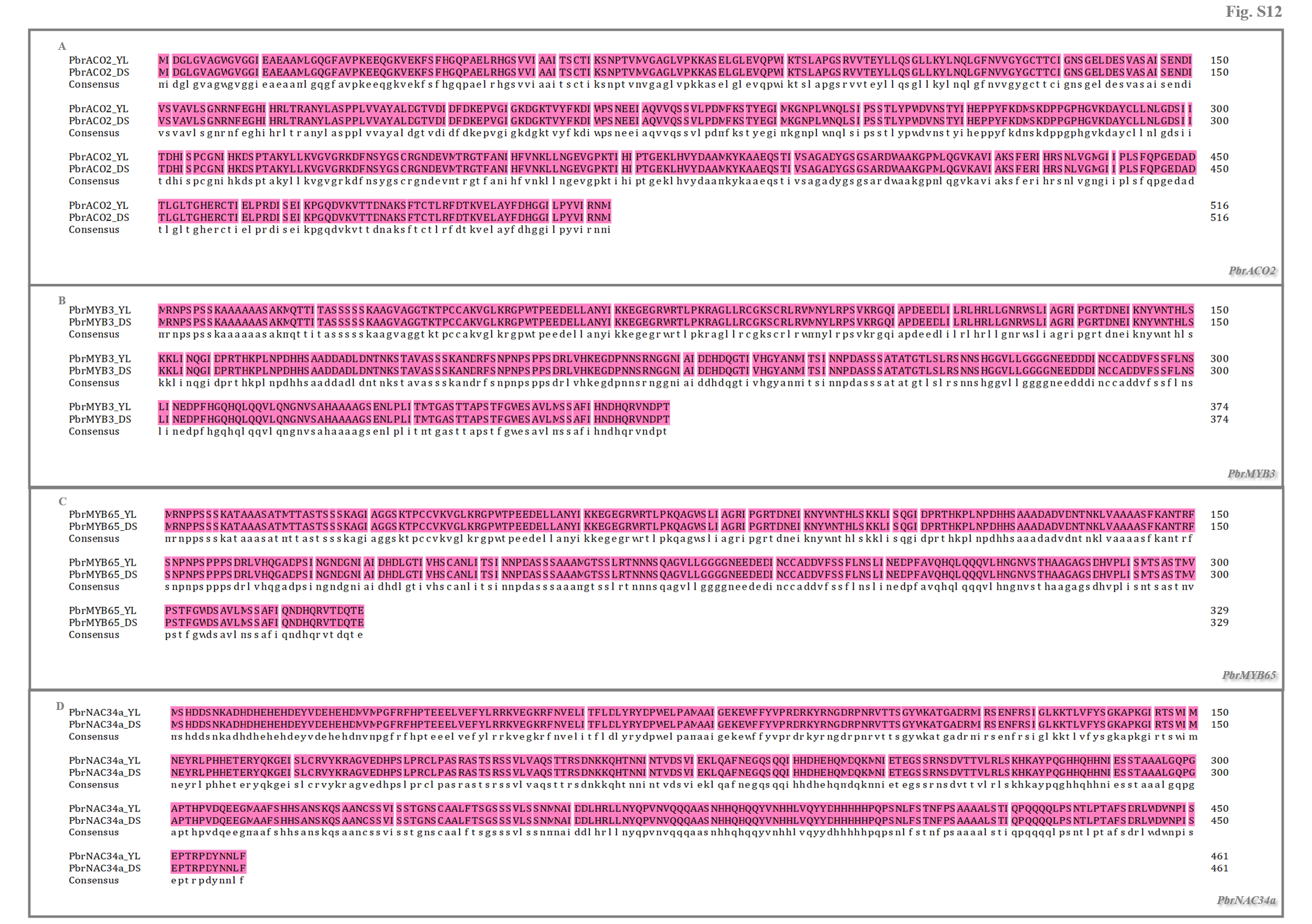


**Fig. S12. Alignment of protein sequences from ‘Yali’ and ‘Dangshansuli’ fruit. (A) PbrACO2. (B) PbrMYB3. (C) PbrMYB65. (D) PbrNAC34a.** Sequence alignment was performed using the DNAMAN software (Lynnon Biosoft, San Ramon, California, USA).


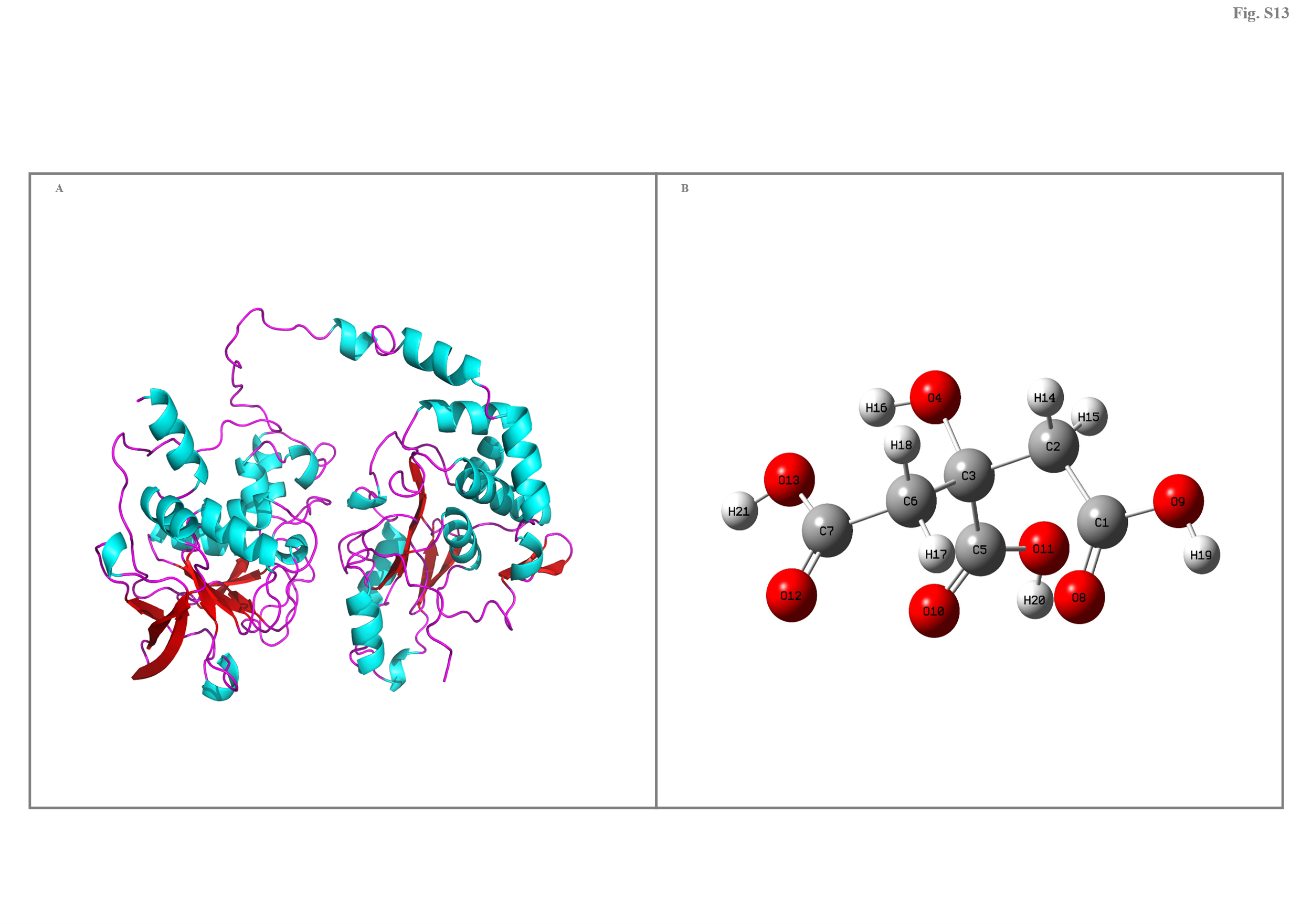


**Fig. S13. 3-D structure of PbrACO2 and citrate. (A) PbrACO2. (B) Citrate.** PbrACO2 protein structure was predicted by AlphaFold2, while citrate structure was converted into a 3-D configuration in Gaussview through geometry optimization (B3LYP with the def2-TZVPP).


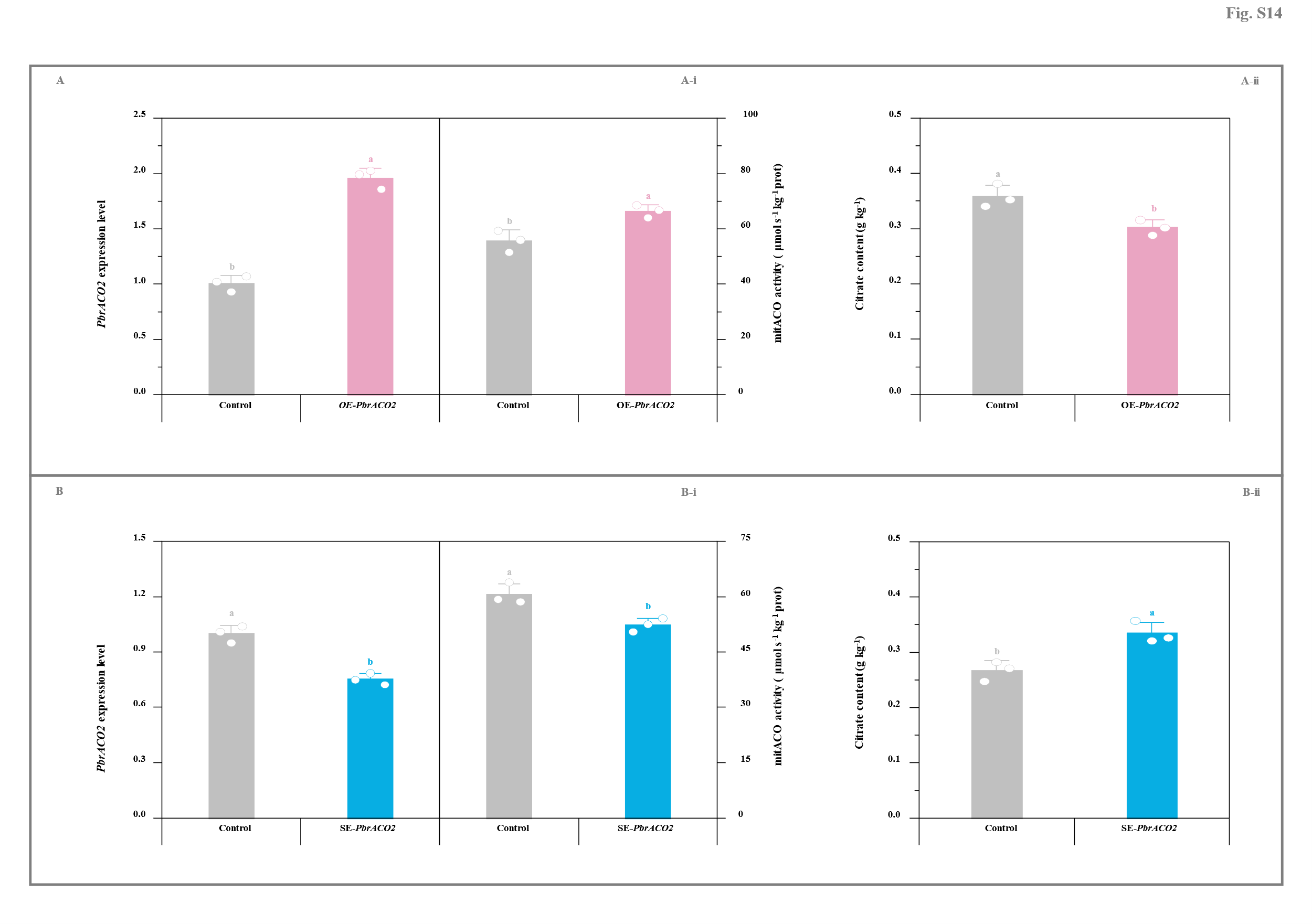


**Fig. S14. Impact of transient transformation of pear fruit with *PbrACO2* gene on citrate metabolism. (A) Transient overexpression of *PbrACO2*. (A-i) *PbrACO2* expression level and mitACO activity. (A-ii) Citrate content.** ‘Yali’ fruit transformed with the empty pCAMBIA1300 vector containing a GFP tag was used as the control for the *PbrACO2*-overexpressing fruit. **(B) Transient silence of *PbrACO2*. (B-i) *PbrACO2* expression level and mitACO activity. (B-ii) Citrate content.** Fruit co-transformed with the empty TRV2 and TRV1 vectors was used as the control for the *PbrACO2*-silenced fruit. The expression level of *PbrACO2* in the control fruit is set as 1.0 for RT-qPCR assay. Data represents mean value ± SD of three biological replicates, and vertical bars labelled with the same small letter are not significantly different between samples (*p* < 0.05).


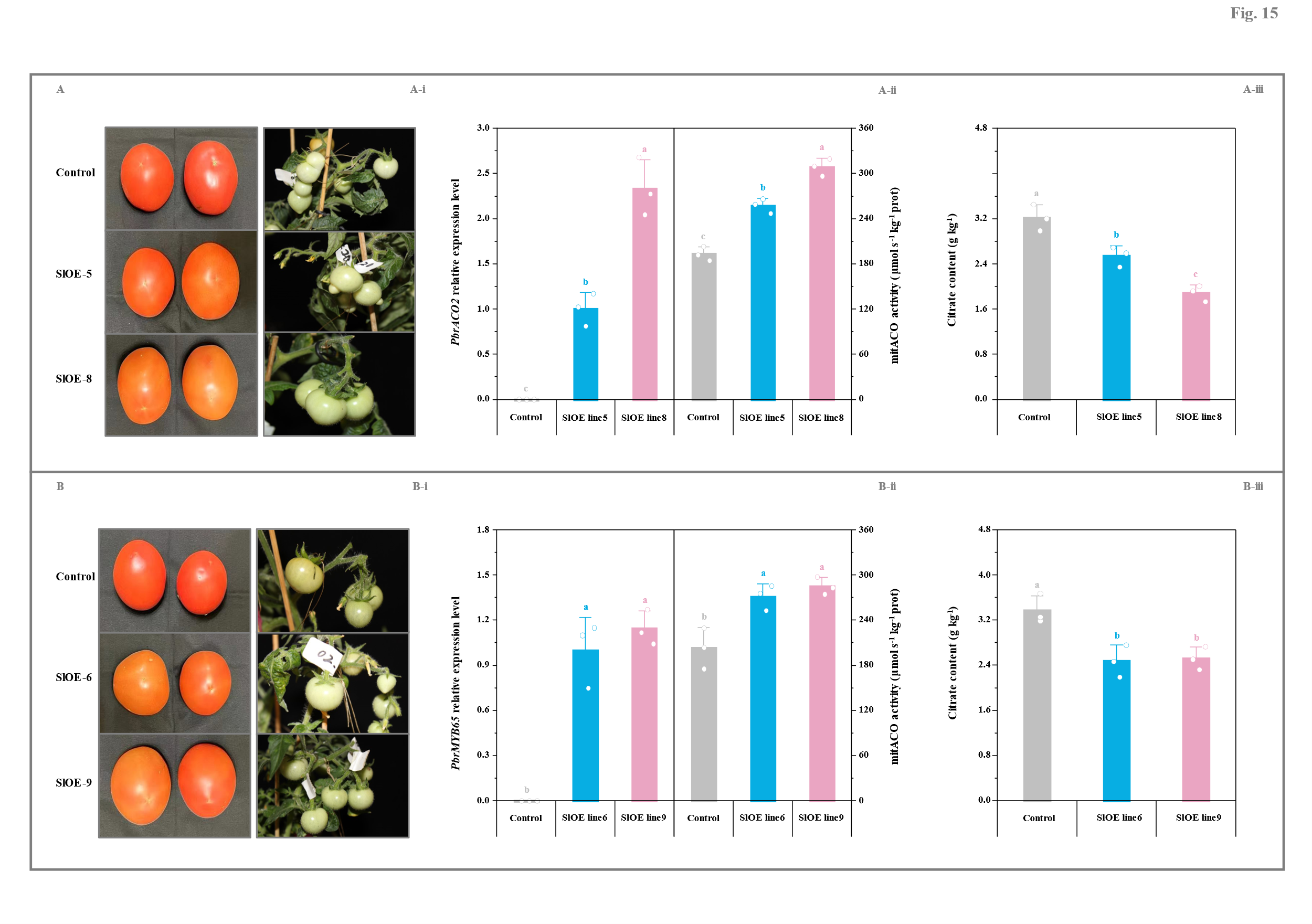


**Fig. S15. Gene function validation in tomato fruit. (A) Function validation of *PbrACO2* gene. (A-i) Phenotypes of the control (wide-type) and transgenic fruit. (A-ii) *PbrACO2* expression level and miACO activity.** The expression level of *PbrACO2* in the *SlOE5*-fruit is set as 1.0 for RT-qPCR assay. **(A-iii) Citrate content. (B) Function validation of *PbrMYB65* gene. (B-i) Phenotypes of the control (wide-type) and transgenic fruit. (B-ii) *PbrMYB65* expression level and miACO activity.** The expression level of *PbrMYB65* in the *SlOE6*-fruit is set as 1.0 for RT-qPCR assay. **(B-iii) Citrate content.** Tomato fruit at 45 DAFB was sampled from the control (wide-type) and transgenic homozygous lines (T2 generation). Data represents mean value ± SD of three biological replicates, and vertical bars labelled with the same small letter are not significantly different between samples (*p* < 0.05).

**
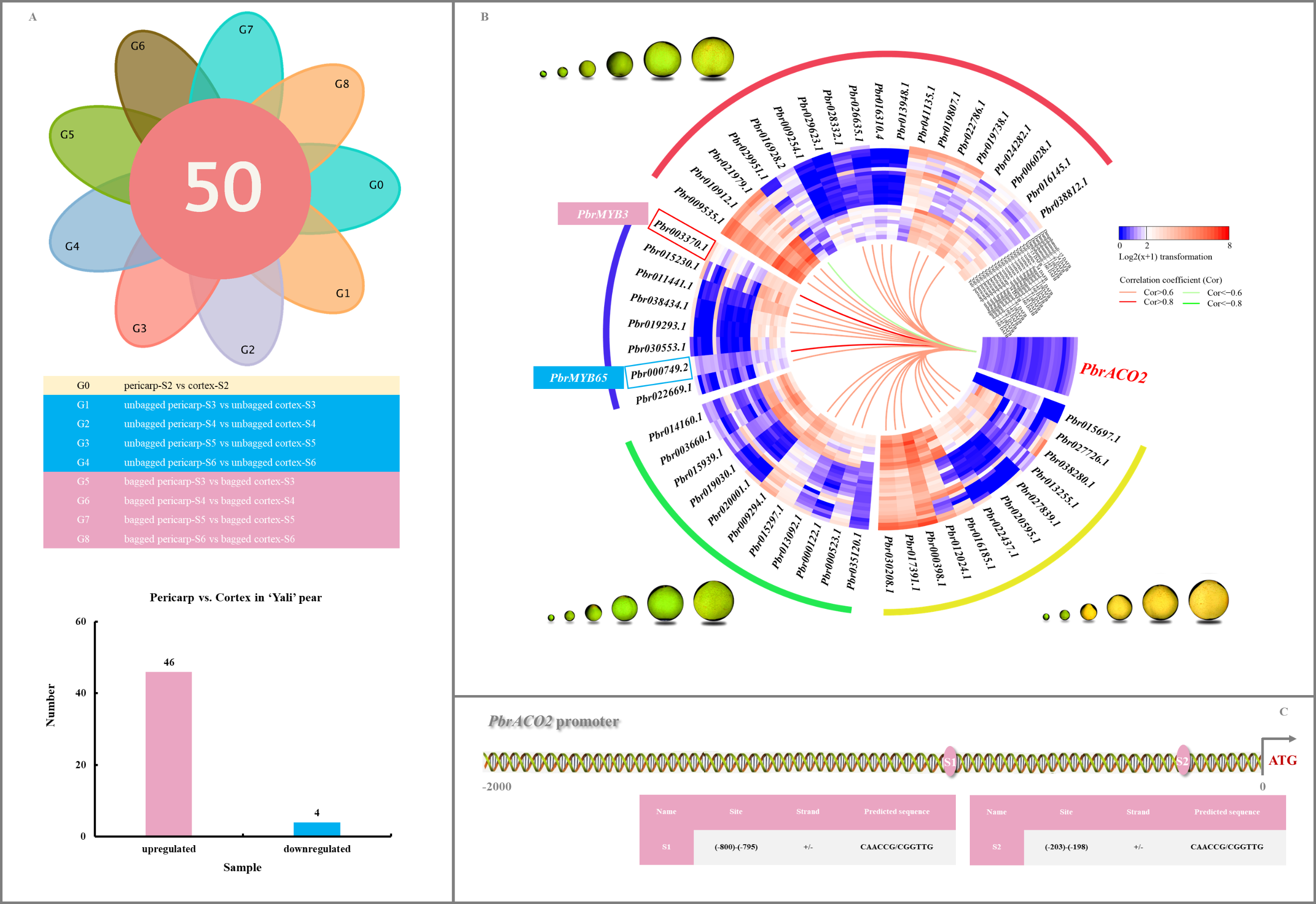
**

**Fig. S16. Characterization of PbrMYB3 and PbrMYB65 as the possible up-restream regulators of *PbrACO2*. (A) Number of the differentially expressed TFs during ‘Yali’ fruit development.** The differentially expressed TFs, whose expression levels in the pericarp tissue were consistently higher or lower than those in the cortex tissue of the developing ‘Yali’ fruit, were identified by DESeq2_EBSeq software, in accordance with the following criteria: fold change ≥ 2.0 and FRD < 0.01. **(B) Expression profiles of the differentially expressed TFs during *P. bretschneideri* Rehd. fruit development and their correlations with *PbrACO2* mRNA abundance.** The color scale represents normalized log2-transformed (FPKM + 1), where red, blue, and white colors indicate high, low, and medium expression levels, respectively. Spearman correlation between different attributes is visualized in the heatmap, where red (or light red) lines demonstrate extremely strong (or strong) positive correlations, while green (or light green) lines indicate extremely strong (or strong) negative associations. *PbrMYB3* (*Pbr003370.1*) is marked in red box and *PbrMYB65* (*Pbr000749.2*) is marked in blue box. **(C) Detailed information of two PbrMYB3/65-binding sites in *PbrACO2* promoter.** The possible binding sites (pink ellipses) of PbrMYB3/65 in *PbrACO2* promoter were predicted by the PlantRegMap database (Tian et al., 2020). ‘Yali’ pear fruit were bagged with triple-layer paper bags at 34 DAFB, while the unbagged ‘Yali’ and ‘Dangshansuli’ fruit at the same positions were labelled as well. Pericarp and cortex tissues were sampled at six developmental stages, including 15 DAFB, 34 DAFB, 81 DAFB, 110 DAFB, 145 DAFB, and 160 DAFB. Data, adapted from transcriptome assay, represent the mean value of three biological replicates, except for gene expression profiles during ‘Dangshansuli’ fruit development (one replicate).


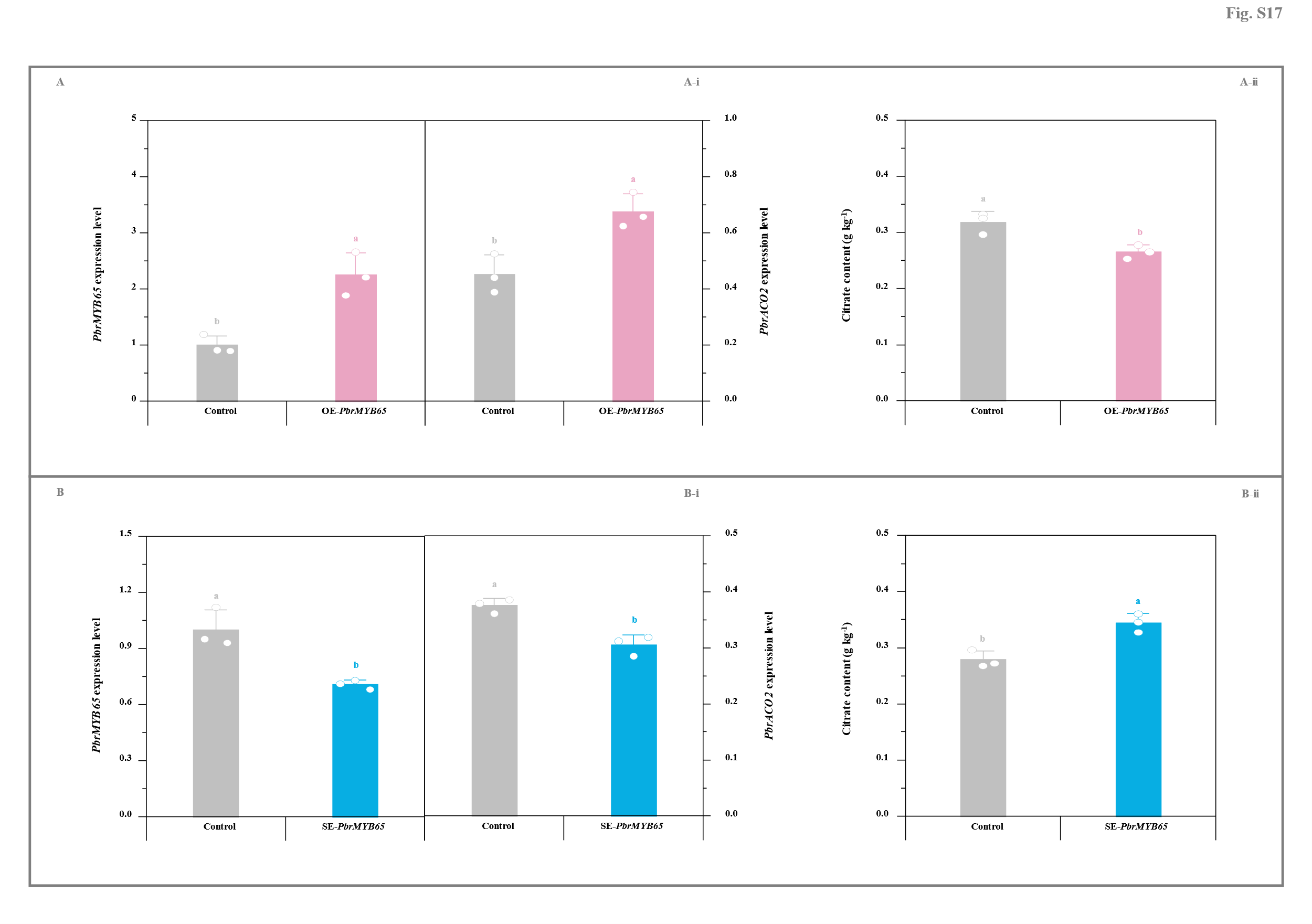


**Fig. S17. Impact of transient transformation of pear fruit with *PbrMYB65* gene on citrate metabolism. (A) Transient overexpression of *PbrMYB65*. (A-i) Expression levels of *PbrMYB65* and *PbrACO2* genes. (A-ii) Citrate content.** ‘Yali’ fruit transformed with the empty pCAMBIA1300 vector containing a GFP tag was used as the control for the *PbrMYB65*-overexpressing fruit. **(B) Transient silence of *PbrMYB65*. (B-i) Expression levels of *PbrMYB65* and *PbrACO2* genes. (B-ii) Citrate content.** Fruit co-transformed with the empty TRV2 and TRV1 vectors was used as the control for the *PbrMYB65*-silenced fruit. The expression level of *PbrMYB65* in the control fruit is set as 1.0 for RT-qPCR assay. Data represents mean value ± SD of three biological replicates, and vertical bars labelled with the same small letter are not significantly different between samples (*p* < 0.05).


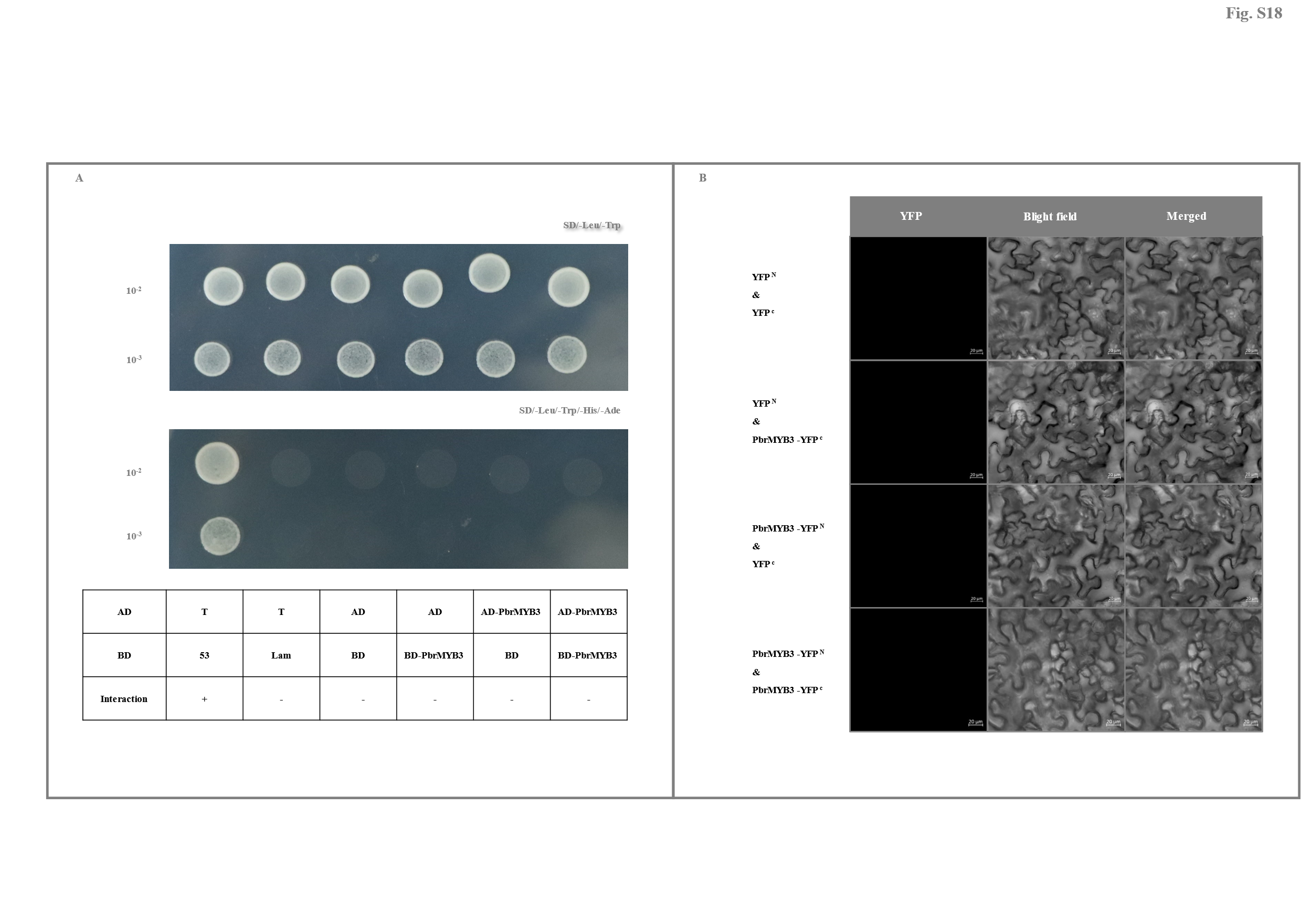


**Fig. S18. PbrMYB3 self-interaction determination. (A) Y2H assay.** Transformants containing AD-*T* & BD-*53*, AD-*T* & BD-*Lam*, AD & BD, AD & BD-*PbrMYB3*, and AD-*PbrMYB3* & BD were used as the controls. **(B) BiFC analyses.** Transformants containing YFP^N^ & YFP^C^, YFP^N^ & *PbrMYB3*-YFP^C^, and *PbrMYB3*-YFP^N^ & YFP^C^ were used as the controls. Bar, 20 μm.


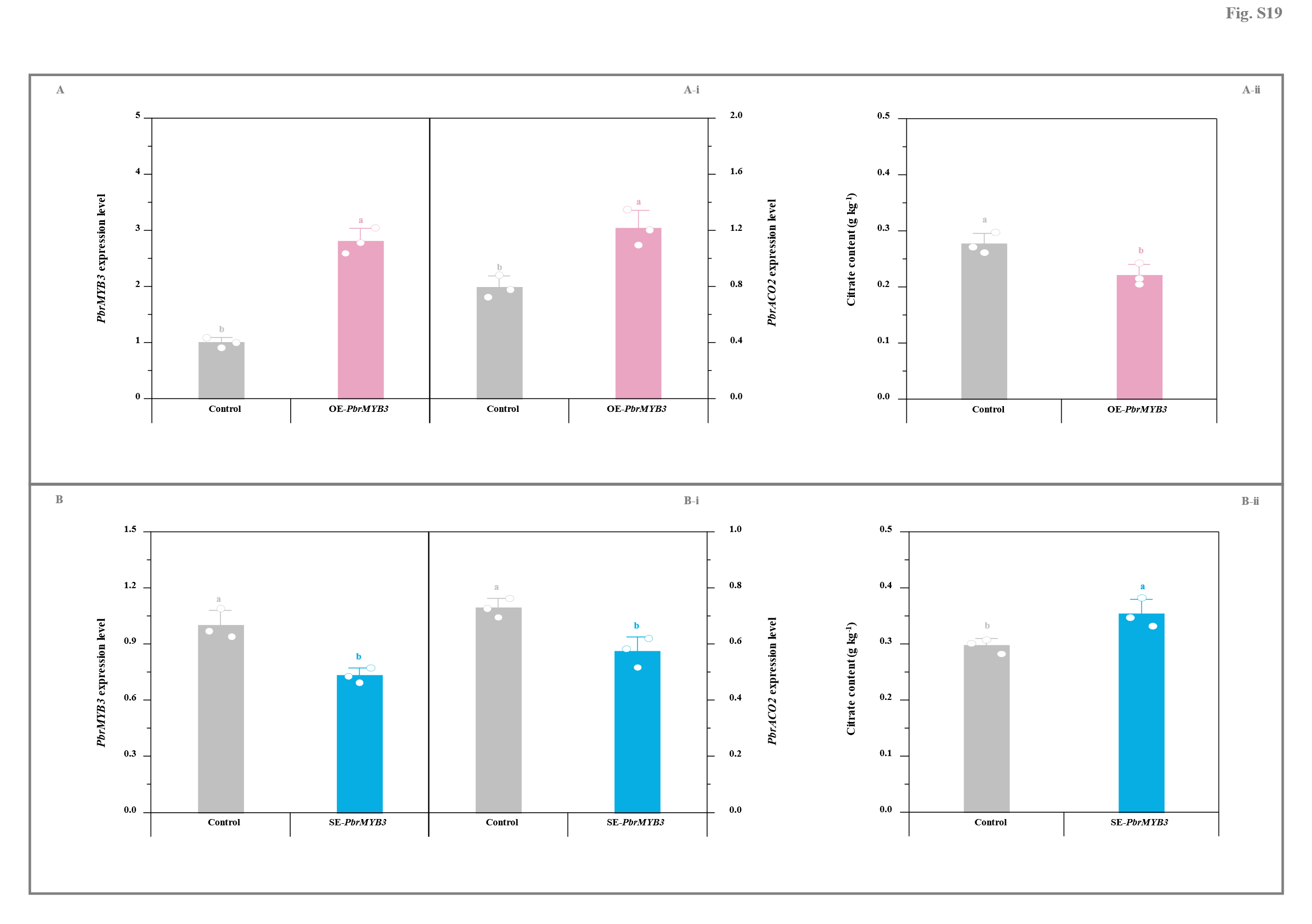


**Fig. S19. Impact of transient transformation of pear fruit with *PbrMYB3* on citrate metabolism. (A) Transient overexpression of *PbrMYB3*. (A-i) Expression levels of *PbrMYB3* and *PbrACO2* genes. (A-ii) Citrate content.** ‘Yali’ fruit transformed with the empty pCAMBIA1300 vector containing a GFP tag was used as the control for the *PbrMYB3*-overexpressing fruit. **(B) Transient silence of *PbrMYB3*. (B-i) Expression levels of *PbrMYB3* and *PbrACO2* genes. (B-ii) Citrate content.** Fruit co-transformed with the empty TRV2 and TRV1 vectors was used as the control for the *PbrMYB3*-silenced fruit. The expression level of *PbrMYB3* in the control fruit is set as 1.0 for RT-qPCR assay. Data represents mean value ± SD of three biological replicates, and vertical bars labelled with the same small letter are not significantly different between samples (*p* < 0.05).


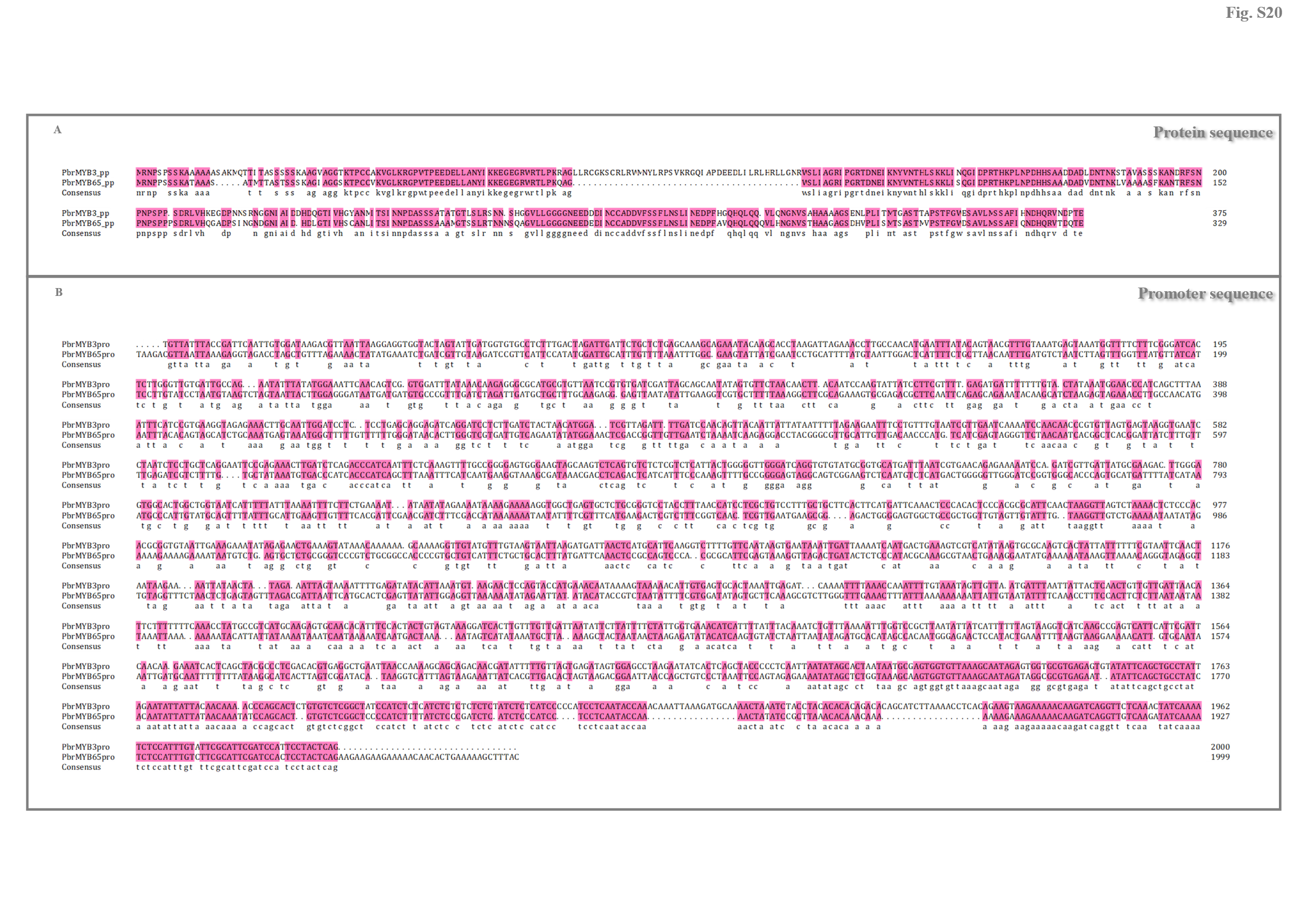


**Fig. S20. Alignment of protein sequences and promoter sequences from ‘Yali’ fruit. (A) PbrMYB3 and PbrMYB65 protein sequences. (B) PbrMYB3 and PbrMYB65 promoter sequences.** Sequence alignment was performed using the DNAMAN software (Lynnon Biosoft, San Ramon, California, USA).


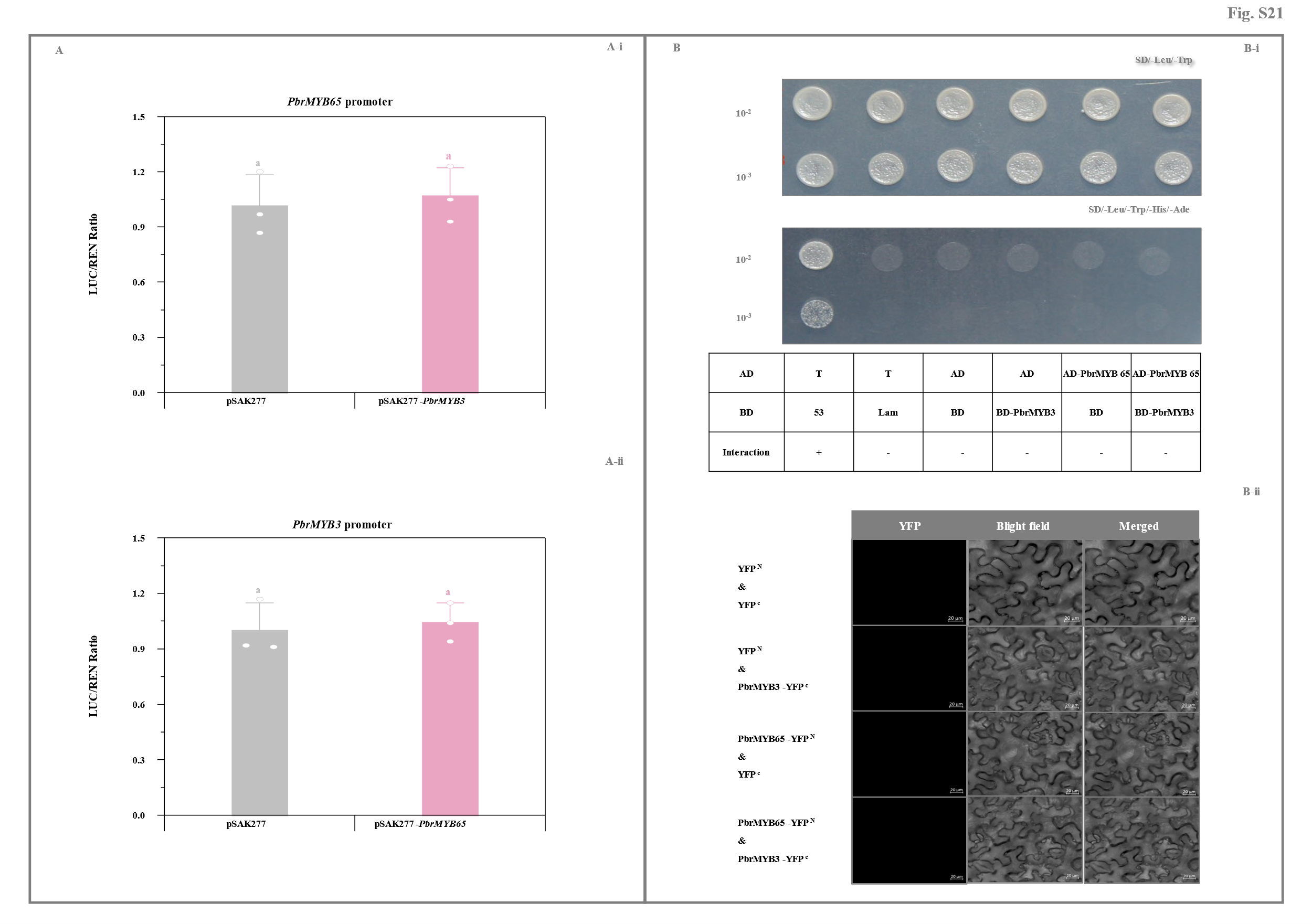


**Fig. S21. Analyses of the relationship between PbrMYB3 and PbrMYB65. (A) Dual-luciferase assay of (A-i) the activation of *PbrMYB65* expression by PbrMYB3 as well as (A-ii) the activation of *PbrMYB3* expression by PbrMYB65.** *PbrMYB3* and *PbrMYB65* CDSs were introduced into the pSAK277 vector, while their promoters into the pGreen 0800-LUC vector. Transformants containing the empty pSAK277 vector and each reporter were used as the controls. Data represents mean value ± SD of three biological replicates, and vertical bars labelled with the same letter are not significantly different between samples (*p* < 0.05). **(B) Determination of the interaction between PbrMYB3 and PbrMYB65. (B-i) Y2H assay.** Transformants containing AD-*T* & BD-*53*, AD-*T* & BD-*Lam*, AD & BD, AD & BD-*PbrMYB3*, and AD-*PbrMYB65* & BD were used as the controls. **(B-ii) BiFC analyses.** Transformants containing YFP^N^ & YFP^C^, YFP^N^ & *PbrMYB3*-YFP^C^, and *PbrMYB65*-YFP^N^ & YFP^C^ were used as the controls. Bar, 20 μm.


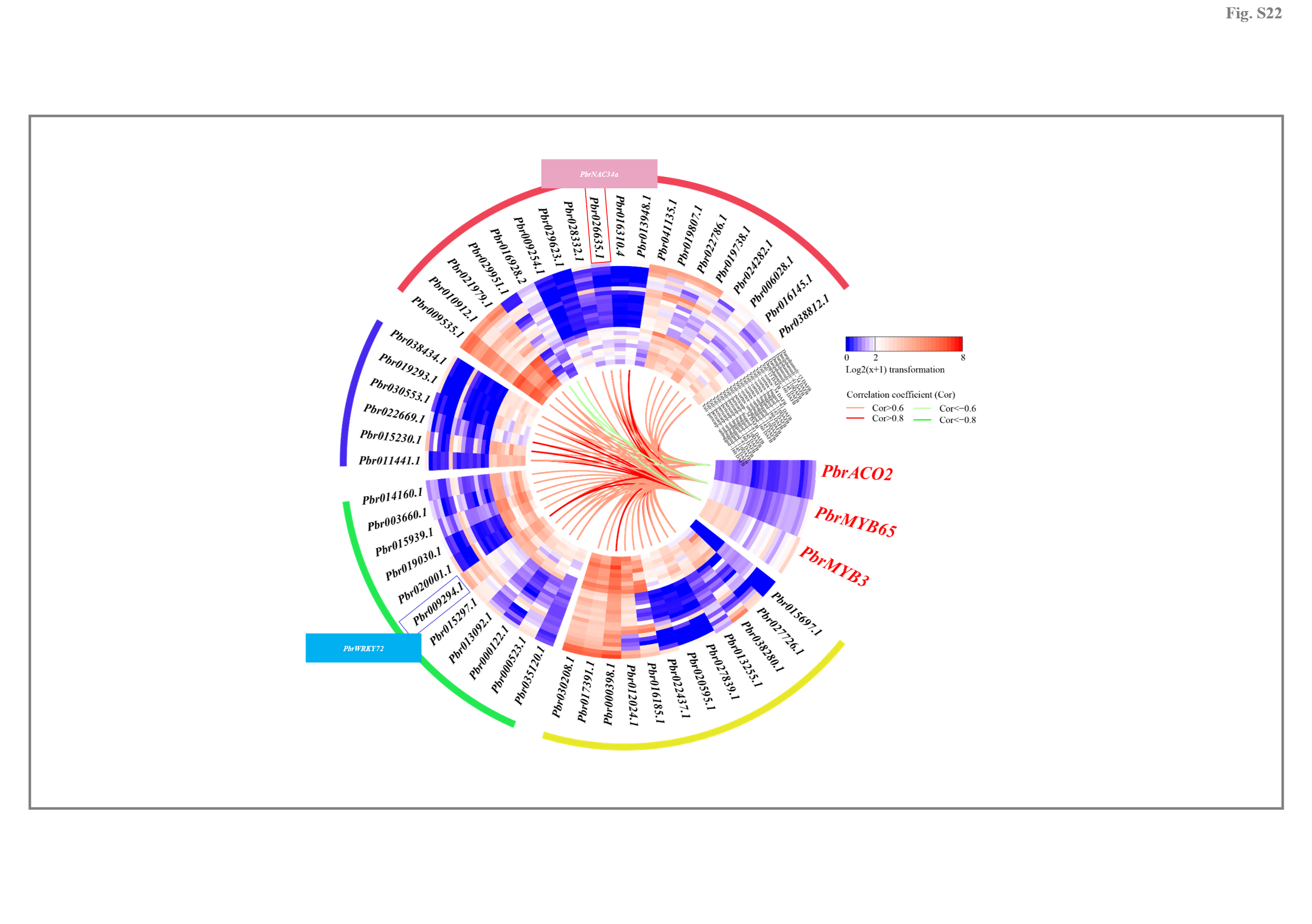


**Fig. S22. Correlations between *PbrMYB3*/*65* and 48 other differential expressed TFs during** ***P. bretschneideri* Rehd. fruit development.** Information on *PbrMYB3* and *PbrMYB65* as well as 48 other differential expressed TFs are summarized in Table S12. The color scale represents normalized log2-transformed (FPKM + 1), where red, blue, and white colors indicate high, low, and medium expression levels, respectively. Spearman correlation between different attributes is visualized in the heatmap, where red (or light red) lines demonstrate extremely strong (or strong) positive correlations, while green (or light green) lines indicate extremely strong (or strong) negative associations. Data, adapted from transcriptome assay, represent the mean value of three biological replicates, except for gene expression profiles during ‘Dangshansuli’ fruit development (one replicate). *PbrNAC34a* (*Pbr026635.1*) is marked in red box, while *PbrWRKY72* (*Pbr009294.1*) in blue box.


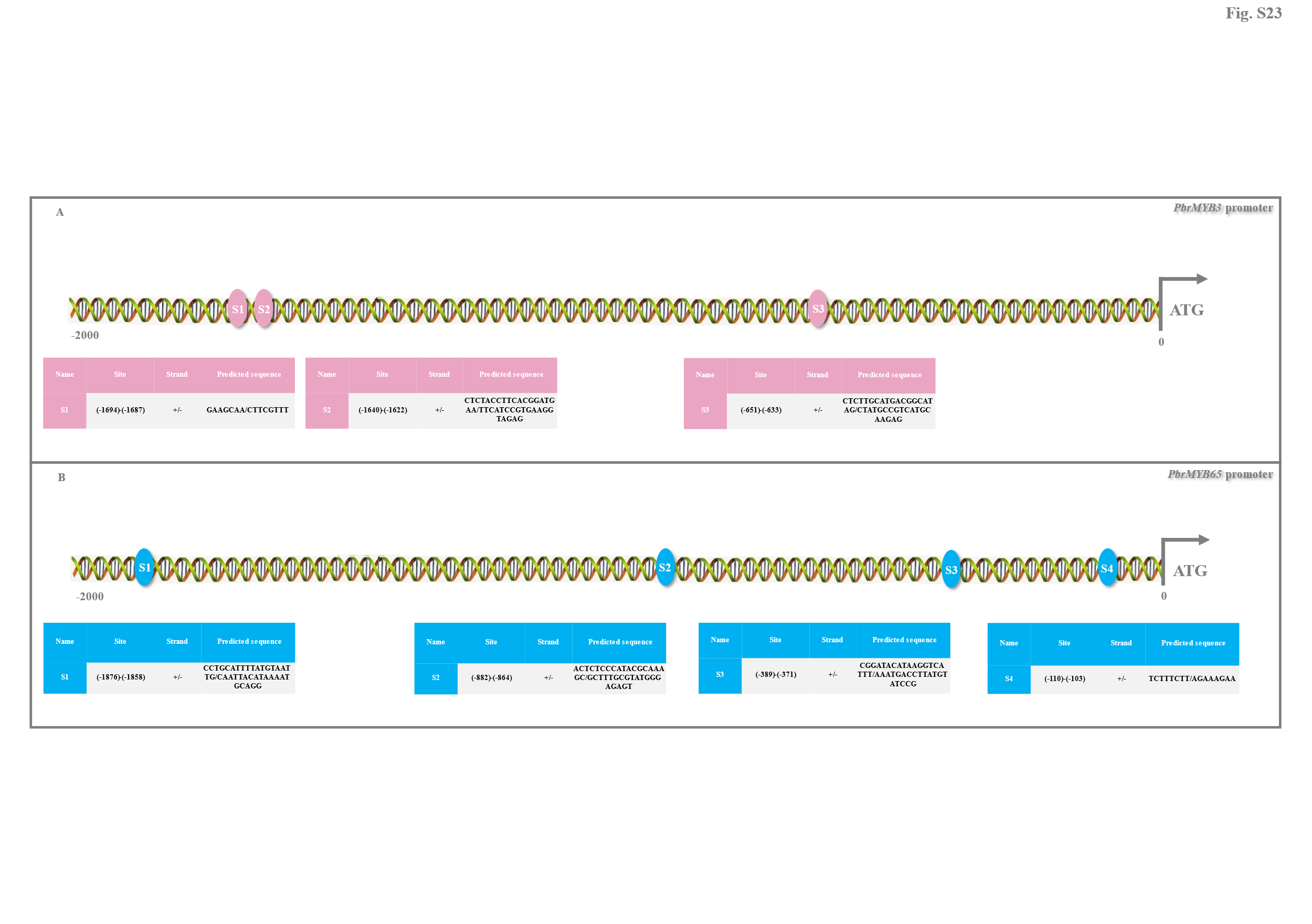


**Fig. S23. Detailed information on the possible PbrNAC34a-binding sites in *PbrMYB3* and *PbrMYB65* promoters. (A) *PbrMYB3* promoter. (B) *PbrMYB65* promoter.** The possible PbrNAC34a-binding sites in *PbrMYB3* (pink ellipses) and *PbrMYB65* (blue ellipses) promoters were characterized with the aid of the PlantRegMap database (Tian et al., 2020) as well as previous reports (Bi et al., 2023; Li et al., 2023).


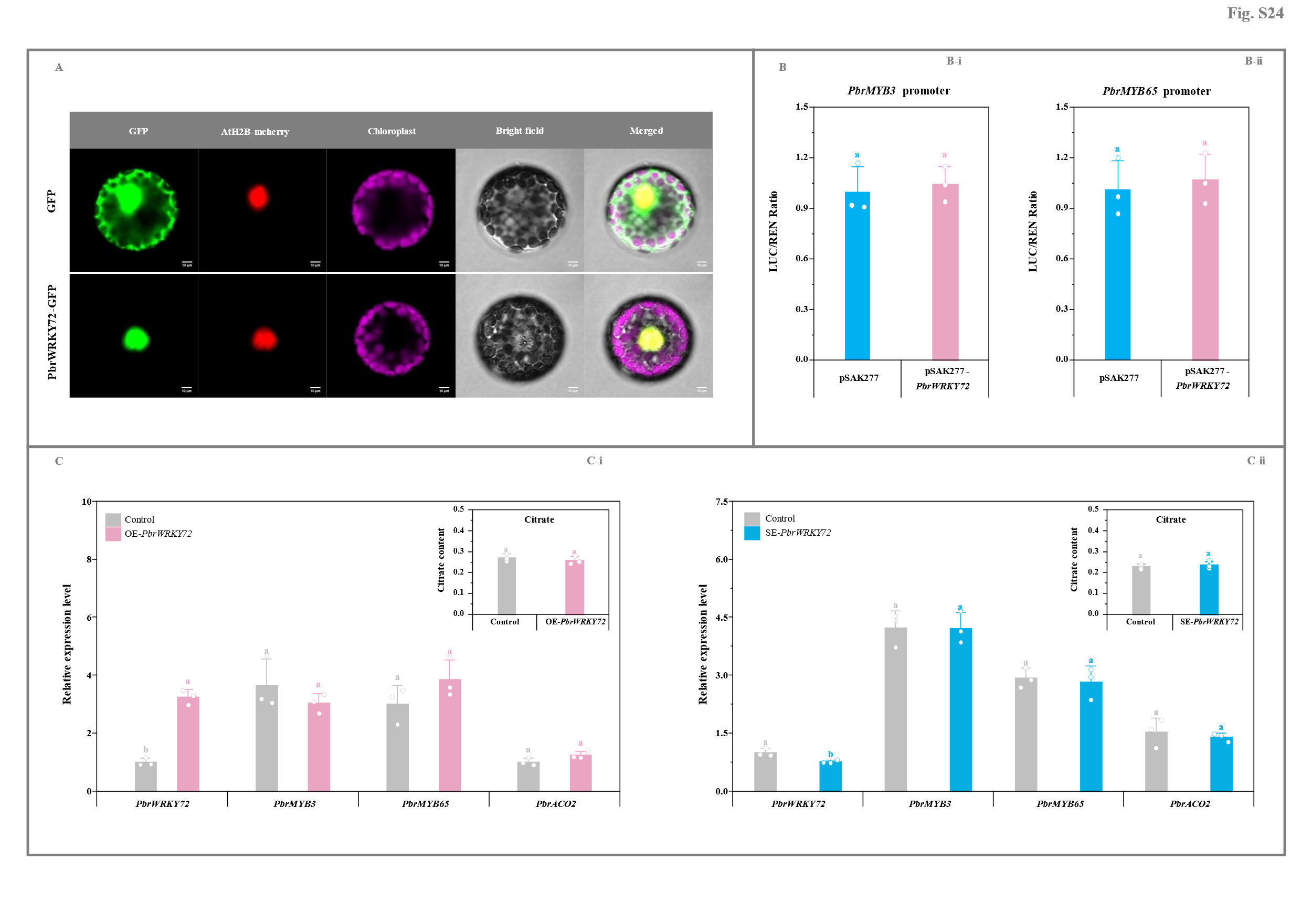


**Fig. S24. Analyses of PbrWRKY72’s role in citrate metabolism. (A) Subcellular localization of PbrWRKY72.** AtH2B-mcherry was used as the nuclear marker (Liu et al., 2007). Bar, 10 μm. **(B) Dual-luciferase assay of the activation of *PbrMYB3* (B-i) and *PbrMYB65* (B-ii) expression by PbrWRKY72.** *PbrWRKY72* CDS was introduced into the pSAK277 vector, while *PbrMYB3* and *PbrMYB65* promoters into the pGreen 0800-LUC vector. Transformants containing the empty pSAK277 vector and each reporter were used as the controls. Data represents mean value ± SD of three biological replicates, and vertical bars labelled with the same letter are not significantly different between samples (*p* < 0.05). **(C) Impact of transient transformation of pear fruit with *PbrWRKY72* on citrate metabolism. (C-i) Transient overexpression of *PbrWRKY72*.** ‘Yali’ fruit transformed with the empty pCAMBIA1300 vector containing a GFP tag was used as the control for the *PbrWRKY72*-overexpressing fruit. **(C-ii) Transient silence of *PbrWRKY72*.** Fruit co-transformed with the empty TRV2 and TRV1 vectors was used as the control for the *PbrWRKY72*-silenced fruit. The expression level of *PbrWRKY72* in the control fruit is set as 1.0 for RT-qPCR assay. Data represents mean value ± SD of three biological replicates, and vertical bars labelled with the same letter are not significantly different between samples (*p* < 0.05).
